# The Pivotal Role of GR‐CAR Pathway in Fetal Programming of Hepatic Cytochrome P450 3A Alteration in Adulthood

**DOI:** 10.1002/advs.202515583

**Published:** 2025-11-16

**Authors:** Xiaoxiang Sun, Jie Liu, E Xiang, Xia Li, Xuerong Yan, Yuxi Wang, Feng Li, Hao Kou, Hui Wang, Yu Guo

**Affiliations:** ^1^ Department of Pharmacology School of Basic Medical Sciences Wuhan University Wuhan 430071 China; ^2^ Hubei Provincial Key Laboratory of Developmentally Originated Disease Wuhan 430071 China; ^3^ Department of Pharmacy Union Hospital Tongji Medical College Huazhong University of Science and Technology Wuhan 430022 China; ^4^ School of Medicine Jingchu University of Technology Jingmen 448000 China; ^5^ Department of Medical Genetics School of Basic Medical Sciences Wuhan University Wuhan 430071 China; ^6^ Department of Pharmacy Zhongnan Hospital of Wuhan University Wuhan 430071 China

**Keywords:** constitutive androstane receptor, cytochrome P450, epigenetic modification, glucocorticoids, glucocorticoid receptor

## Abstract

Multiple prenatal adverse environmental factors alter hepatic cytochrome P450 (CYP) enzymes expression in offspring, with these changes persisting after birth. These factors induce fetal exposure to excessive maternal glucocorticoids (GCs), however, the mechanisms by which intrauterine GCs exposure programs offspring CYP expression remain unclear. Given that GCs are high‐affinity ligands for the glucocorticoid receptor (GR), this work employs dexamethasone (DEX), a GR agonist, to establish a prenatal dexamethasone exposure (PDE) model for investigating the role of GR activation in CYPs programming. The model is implemented to pregnant Wistar rats and heterozygous liver‐specific GR knockout mice. Results show that PDE consistently increases the expression of hepatic CYP3A1 and CYP2B1, thereby enhancing the metabolic enzyme efficiency in adult male offspring. In vitro experiment further validates that GC‐induced activation of GR increases the binding of P300/cAMP response element‐binding protein (CBP) to the promoter region of constitutive androstane receptor (CAR), which leads to sustained H3K9 and H3K27 acetylation at the CAR locus, indirectly promoting CYP expression in adult offspring. Conclusively, the GR‐CAR pathway may play a pivotal role in programming CYP3A alteration in offspring exposed to elevated intrauterine GCs. This study provides novel insights into individual variations of CYPs expression and metabolic patterns.

## Graphical abstract

Prenatal dexamethasone exposure (PDE) persistently upregulates hepatic CYP3A1/CYP2B1 in offspring via glucocorticoid receptor (GR) activation. In vivo and in vitro studies show GR promotes P300/CBP binding to the constitutive androstane receptor (CAR) promoter, sustaining histone acetylation (H3K9/K27ac) and enhancing CYP expression. The GR–CAR pathway programs metabolic changes, explaining individual variation in drug metabolism.

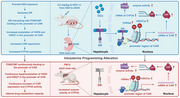

## 1 Introduction

The cytochrome P450 enzymes (CYPs) constitute a major hepatic superfamily essential for metabolizing both endogenous and exogenous compounds, primarily via the CYP1‐3 subfamilies.^[^ ^1^, ^2^ ^]^ The CYP1‐3 families are primarily responsible for the metabolism of a broad spectrum of xenobiotics, including the majority of clinically administered drugs, accounting for approximately 70‐80% of human drug metabolism.^[^ ^3^ ^]^ Among these, CYP3A is particularly important, comprising about 30% of total hepatic CYPs and metabolizing roughly 50% of commercially available pharmaceutical agents.^[^ ^4^ ^]^ Most cases of individual variability in drug metabolism among patients can be attributed to interindividual differences in the expression and activity of CYP1‐3.^[^ ^5^ ^]^ In addition to a wide range of clinical drugs and environmental toxicants, the substrates of CYP1‐3 also encompass various endogenous substances, including vitamins, bile acids, and steroid hormones.^[^ ^3^, ^6^, ^7^, ^8^ ^]^ Previous animal studies have shown that adverse environmental exposures in early life could persistently alter the CYP1‐3 expression in offspring before and after birth, for instance, protein restriction during lactation has been evidenced to upregulate both the expression and enzymatic activity of multiple CYP isoforms in rat offspring, including CYP1A1, 1A2, 2B1, 2C11, and 3A;^[^ ^9^, ^10^ ^]^ gestational exposure to procymidone results in sustained elevation of hepatic CYP3A expression in mice, persisting through postnatal week (PW) 7;^[^ ^11^ ^]^ prenatal ethanol exposure leads to long‐standing elevation of mRNA expression of multiple CYP isoforms in adult rats.^[^ ^12^ ^]^ Collectively, these studies suggested a developmental origin for CYP1‐3 expression characterized by maternal effects. Nevertheless, the mechanism by which adverse prenatal factors cause enduring changes in CYP expression remains ambiguous.

Numerous studies have demonstrated that fetal exposure to excessive maternal glucocorticoids (GCs) frequently occurs under compromised maternal environments, including maternal chronic stress, xenobiotic exposure, and nutritional deficiencies.^[^ ^13^, ^14^, ^15^, ^16^, ^17^ ^]^ Under these conditions, the maternal hypothalamic‐pituitary‐adrenocortical (HPA) axis could be activated, leading to increased synthesis and secretion of maternal GCs.^[^ ^18^ ^]^ Placental GC barrier is composed of GC‐inactivating enzyme (11β‐hydroxysteroid dehydrogenase type 2, 11β‐HSD2) and efflux transporter (P‐glycoprotein, P‐gp). It only partially restricts the transfer of maternal GCs to the fetus^[^ ^19^ ^]^ and might be further impaired,^[^ ^20^ ^]^ leading to fetal exposure to excessive maternal GCs. Physiological levels of endogenous GCs are essential for fetal development, particularly for organ maturation during late gestation. However, excessive maternal GC exposure, especially during early pregnancy, can disrupt fetal organ development trajectory and predispose offspring to physiological dysfunction in adulthood.^[^ ^21^ ^]^ Prenatal administration of synthetic GCs, such as dexamethasone (DEX) or betamethasone, has been shown to alter organ function in adult offspring,^[^ ^22^, ^23^, ^24^ ^]^ which supports the critical role of GCs in developmental programming. Our previous study demonstrated that prenatal ethanol exposure elevated fetal blood GCs levels^[^ ^25^ ^]^ and induced sustained upregulation of CYP2B1 and CYP3A1 mRNA expression in offspring.^[^ ^12^ ^]^ In vitro experiments revealed that GCs synergistically enhanced ethanol‐induced CYP expression, with GCs exhibiting a more pronounced effect than ethanol alone.^[^ ^12^ ^]^ These findings suggest maternal GCs may play a critical role in altering CYP expression in offspring following adverse prenatal exposures. As high‐affinity ligands for the glucocorticoid receptor (GR), GCs activate GR, leading to homodimer formation in cytoplasm. These homodimer complexes translocate to the nucleus, where they bind to glucocorticoid response elements (GREs) in the promoter regions of target genes, thereby modulating the transcription of downstream genes.^[^ ^26^, ^27^ ^]^ To sum up, these findings indicate that GR activation may play a critical role in the programming of CYPs expression in offspring exposed to adverse factors prenatally, warranting further investigation.

Studies have revealed that long‐lasting alterations of gene expression in offspring resulting from adverse prenatal factors are closely associated with epigenetic modifications.^[^ ^28^ ^]^ The alteration of epigenetic modification patterns and expression of essential genes induced by adverse maternal environments may be involved in the increased susceptibility to multiple chronic diseases (e.g., metabolic syndrome, cardiovascular disease, and behavioural disorders) in offspring.^[^ ^29^ ^]^ Unlike the stable conservation of DNA methylation, histone modifications exhibit greater activity and plasticity during development.^[^ ^30^ ^]^ Moreover, histone modifications are primarily involved in changes of CYP gene expression in offspring exposed to suboptimal developmental conditions. For instance, prenatal exposure to cypermethrin increases CYP1A and CYP2B expression in the brain and liver of adult offspring by enhancing acetylation of multiple lysine residues on histone 3 (H3) at the promoter regions of the CYP1A and CYP2B genes.^[^ ^31^, ^32^ ^]^ Furthermore, activation of constitutive androstane receptor (CAR) by 1,4‐bis[2‐(3,5‐dichloropyridyloxy)] benzene (TCPOBOP) in neonatal mice increases levels of H3K4 mono‐, di‐, and tri‐methylation and decreased H3K9 tri‐methylation at the *Cyp2b10* and *Cyp3c27* promoters in offspring, resulting in sustained upregulation these genes in adult offspring.^[^ ^33^ ^]^ However, we hypothesized that histone modifications may also be associated with persistent changes of CYP expression in offspring exposed to excessive maternal GC.

To investigate role of GR in fetal programming of CYP, we utilized GR agonists (e.g., DEX and prednisone) as positive controls in preliminary experiments. We observed a sustained increase in hepatic CYPs expression in rat offspring following maternal exposure to these agonists during pregnancy. DEX, a classical GR agonist, is extensively employed to study the effects of GR activation,^[^ ^34^, ^35^, ^36^ ^]^ and it is commonly used as a positive control reagent to examine the impact of high GC on fetal development,^[^ ^37^, ^38^, ^39^, ^40^, ^41^ ^]^ due to its ability to readily cross the placental barrier into fetal circulation.^[^ ^24^ ^]^ Consequently, this study aims to investigate the intrauterine programming mechanism underlying altered hepatic CYP3A expression, one of the predominant isoforms, in adult offspring exposed to excessive maternal GCs prenatally, using a well‐characterized model of prenatal DEX exposure (PDE).

## 2 Results

### 2.1 PDE Upregulated CYP3A Expression and Activity in Male Offspring

**Figure 1:**
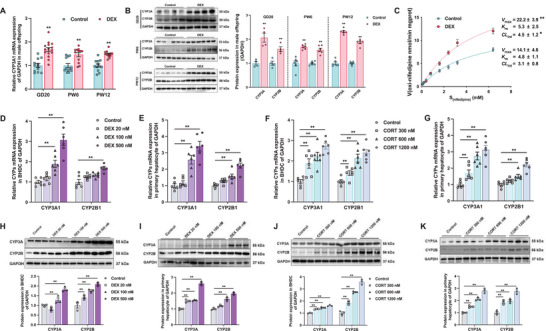
Prenatal dexamethasone exposure (PDE) upregulated CYP3A expression and activity in male offspring. A) The mRNA expression of CYP3A1 in male offspring on GD20, in postnatal week 6 (PW6) and PW12, *n* = 12, dexamethasone (DEX): 0.2 mg kg^−1^ d^−1^ dexamethasone in GD9‐20. B) The protein level of CYP3A1 and CYP2B1 in male offspring on gestational day 20 (GD20), in PW6 and PW12, *n* = 4–6. C) The plot of Michaelis–Menten curves showed the correlation of reaction velocities and various concentration of cytochrome P450 3A (CYP3A) substrate nifedipine and calculated kinetic parameters of intrinsic clearance (CL_int_), maximum velocity (*V*_max_), and Michaelis constant (*K*_m_) by liver microsomes of male offspring in PW12, *n* = 6; the mRNA expression of CYP3A1 and CYP2B1 in (D) bone marrow mesenchymal stem cells hepatoid differentiated cell (BHDC) and (E) primary hepatocyte after treatments of 0, 20 × 10 ^−9^, 100 × 10 ^−9^, and 500 × 10 ^−9^ m DEX, *n* = 6; F,G) the mRNA expression of CYP3A1 and CYP2B1 in F) BHDC and G) primary hepatocyte after treatments of 0, 300 × 10 ^−9^, 600 × 10 ^−9^, and 1200 × 10 ^−9^ m corticosterone (CORT), *n* = 6; the protein expression of CYP3A1 and CYP2B1 in H) BHDC and I) primary hepatocyte after treatments of 0, 20 × 10 ^−9^, 100 × 10 ^−9^, and 500 × 10 ^−9^ m DEX, *n* = 3; the protein expression of CYP3A and CYP2B in J) BHDC and K) primary hepatocyte after treatments of 0, 300 × 10 ^−9^, 600 × 10 ^−9^, and 1200 × 10 ^−9^ m CORT, *n* = 3. The data are presented as mean ± S.E.M., *p < 0.05, **p < 0.01 versus control.

The expression of CYP1‐3 isoform was assessed in both male and female offspring at multiple time points. Results showed that the expression of hepatic CYP3A1 in male PDE offspring was consistently and significantly increased on GD20, in PW6, and PW12 (*p* < 0.05, *p* < 0.01, **Figure** **1**A,B). In enzyme kinetic assay, the intrinsic clearance (CL_int_) and the maximum velocity (*V*_max_) for the CYP3A substrate nifedipine, catalyzed by liver microsomes from PW12 male offspring, were higher in PDE group than in the control group (*p* < 0.05, *p* < 0.01, Figure 1C), while *K*_m_ value remained unchanged. These alterations indicated an increased metabolic rate for nifedipine of male PDE offspring. In vivo pharmacokinetic study revealed a notably lower plasma concentration‐time curve in PDE male offspring (Figure S1A, Supporting Information). Key pharmacokinetic parameters, including the area under the plasma concentration‐time curve (AUC), mean residence time (MRT), time to reach maximum concentration (*T*_max_), maximum concentration (*C*_max_), elimination half‐time (*t*_1/2β_), and elimination rate constant (*K*), were significantly reduced in PDE offspring, suggesting enhanced first pass‐elimination effect and accelerated metabolism of nifedipine in the PDE offspring (*p* < 0.05, Table S6, Supporting Information). Similarly, hepatic CYP2B1 expression in male PDE offspring was induced and sustained from GD20 to PW12 (*p* < 0.05, *p* < 0.01, Figure 1B, Figure S1C,E, Supporting Information). However, no persistent alterations were observed in the expression of other CYPs. In contrast, female offspring showed no consistent changes in CYP1‐3 expression (Figure S1B,D,F, Supporting Information). A sustained increase in the expression of CYP3A1 and CYP2B1 was also observed in GD20 and PW12 male offspring exposed to prednisone prenatally, exhibiting a dose‐dependent effect (*p* < 0.05, *p* < 0.01, Figure S1G–H, Supporting Information). Subsequently, BHDC and rat fetal primary hepatocytes were treated with varying concentrations of DEX or corticosterone (CORT). Results showed that both two GCs significantly induced CYP3A and CYP2B expression at both the gene and protein levels, in a concentration‐dependent manner (*p* < 0.05, *p* < 0.01, Figure 1D–K). Previous studies indicated that changes in CYP3A expression in offspring due to adverse prenatal factors are often accompanied by concordant changes in CYP2B expression.^[^ ^32^, ^42^, ^43^ ^]^ suggesting that a common regulatory mechanism may underlie the programming changes in these two CYP subtypes. Additionally, considering that CYP expression in adult female rat is highly influenced by the estrous cycle and growth hormone secretion patterns,^[^ ^44^, ^45^ ^]^ and based on sex difference in progeny's CYPs expression patterns in this study, subsequent experiments will focus exclusively on elucidating the mechanism of CYP expression programming in male offspring rats.

### 2.2 GR Mediated CYP3A Induction in PDE Male Offspring

**Figure 2:**
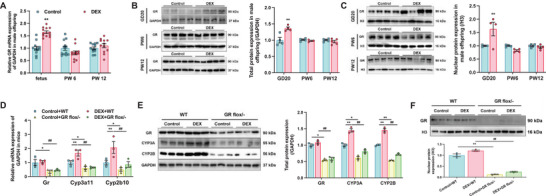
Glucocorticoid receptor (GR) mediated the induction of cytochrome P450 3A (CYP3A) expression after prenatal dexamethasone exposure (PDE) in male offspring. A) The mRNA expression of GR in male offspring on gestational day 20 (GD20), at postnatal week 6 (PW6) and PW12, *n* = 10–12, dexamethasone (DEX): 0.2 mg kg^−1^ d^−1^ dexamethasone in GD9‐20; the B) total and C) nuclear protein content of hepatic GR in male offspring on GD20 and in PW6 and PW12, *n* = 4–5; the D) Gr and Cyp3a11, Cyp2b10 mRNA, E) total protein expression of GR, CYP3A, and CYP2B and F) nuclear protein expression of GR in GR^flox/−^ mice in PW12, *n* = 3–6, DEX: 0.1 mg kg^−1^ d^−1^ dexamethasone in GD9‐18. The data are presented as mean ± S.E.M., *p < 0.05, **p < 0.01 versus control, ^#^ *p* < 0.05, ^##^ *p* < 0.01 versus negative control.

GCs function as the ligand to activate GR and promote its expression.^[^ ^46^ ^]^ Our findings demonstrated that mRNA, total and nuclear protein levels of GR were significantly elevated in the male offspring of the PDE group compared to the control group on GD20, however, no remarkable changes were observed in PW6 and PW12 (*p* < 0.05, *p* < 0.01, **Figure** **2**A–C). To validate GR activation, we analyzed the expression of FK506 binding protein 5 (FKBP5), a canonical GR‐target gene as positive control.^[^ ^47^ ^]^ Notably, significant transcriptional changes in FKBP5 were also observed exclusively on GD20 (*p* < 0.05, *p* < 0.01, Figure S2B, Supporting Information). Given the critical role of GR in regulating genes associated with development and survival in vertebrates,^[^ ^48^, ^49^ ^]^ we utilized heterozygous mice with liver‐specific GR knockout to investigate the impact of partial GR loss on CYP3A and CYP2B expression during mid‐to‐late pregnancy (GD9‐18) (the map of final targeting vector of mice Nr3c1 and the site design of Nr3c1 knockout are shown in Figure S2C,D, Supporting Information). Hepatic GR protein levels were reduced in GR^flox/− Alb‐Cre/Alb‐Cre^ mice, with no significant changes observed in other organs (Figure S2E, Supporting Information). Furthermore, assessment of various liver functional gene expressions confirmed that liver‐specific GR knockdown did not affect these genes in mice (Figure S2F, Supporting Information). PDE significantly increased gene and protein expression of hepatic CYP3A11 and CYP2B10 (which were the homologous proteins of rat CYP3A1 and CYP2B1 in male mice^[^ ^50^ ^]^) in WT male offspring, but unaffected expression levels of the two isoforms in GR^flox/− Alb‐Cre/Alb‐Cre^ mice. In adult offspring mice, hepatic GR expression remained unchanged after PDE in both WT and GR^flox/−^ groups (Figure 2D–F).

**Figure 3:**
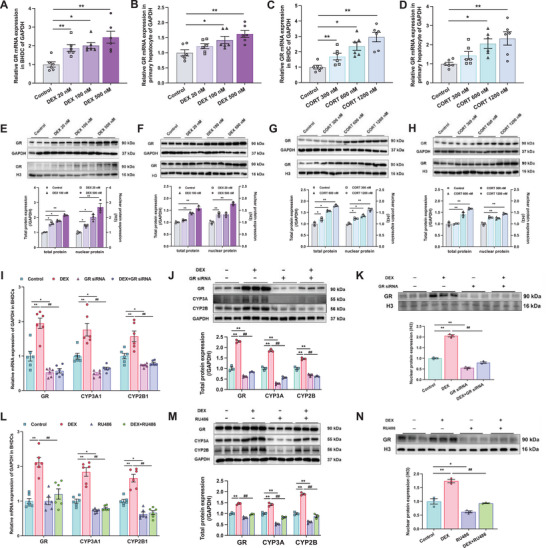
Glucocorticoid receptor (GR) played a key role in the regulation of cytochrome P450 3A (CYP3A) expression in bone marrow mesenchymal stem cells hepatoid differentiated cell (BHDC). The mRNA expression of GR in A) BHDC and B) primary hepatocyte after treatments of 0, 20 × 10^−9^, 100 × 10^−9^, and 500 × 10^−9^ m dexamethasone (DEX), *n* = 6; the mRNA expression of GR in C) BHDC and D) primary hepatocyte after treatments of 0, 300 × 10^−9^, 600 × 10^−9^, and 1200 × 10^−9^ m CORT, *n* = 6; the total and nuclear protein expression of GR after treatments of E) DEX and F) corticosterone (CORT), *n* = 3; the total and nuclear protein expression of GR after treatments of G) DEX and H) CORT in primary hepatocyte, *n* = 6; the I) mRNA of GR, CYP3A1, CYP2B1 and J) total protein expression of GR, CYP3A, CYP2B, and K) nuclear protein expression of GR in BHDC after treatments of 500 × 10^−9^ m DEX or/and 100 × 10^−9^ m GR small interference RNA (siRNA), *n* = 3–6; the L) mRNA of GR, CYP3A1, CYP2B1, and M) total protein expression of GR, CYP3A, CYP2B, and N) nuclear protein expression of GR in BHDC after treatments of 500 × 10^−9^ m DEX or/and 10 × 10^−6^ m mifepristone (RU486), *n* = 3–6. The data are presented as mean ± S.E.M., *p < 0.05, **p < 0.01 versus control, ^#^ *p* < 0.05, ^##^ *p* < 0.01 versus negative control.

In both primary hepatocytes and BHDC, GCs treatment induced GR mRNA level and nuclear translocation in a concentration‐dependent manner (*p* < 0.05, *p* < 0.01, **Figure** **3**A–H). Treatment with GR siRNA significantly attenuated the DEX‐induced upregulation of CYP3A1, CYP2B1, and GR in BHDC (*p* < 0.05, *p* < 0.01, Figure 3I–K). RU486, a competitive GR antagonist,^[^ ^51^ ^]^ significantly inhibited protein nuclear translocation of GR and expression of CYPs induced by DEX treatment (*p* < 0.05, *p* < 0.01, Figure 3L–N). These results confirmed crucial role of GR in modulating CYP3A and CYP2B exposed to GCs, and suggested that GR regulates the physiological expression of these enzymes.

### 2.3 GR‐CAR Pathway Mediated GC‐Induced CYP3A Upregulation

**Figure 4:**
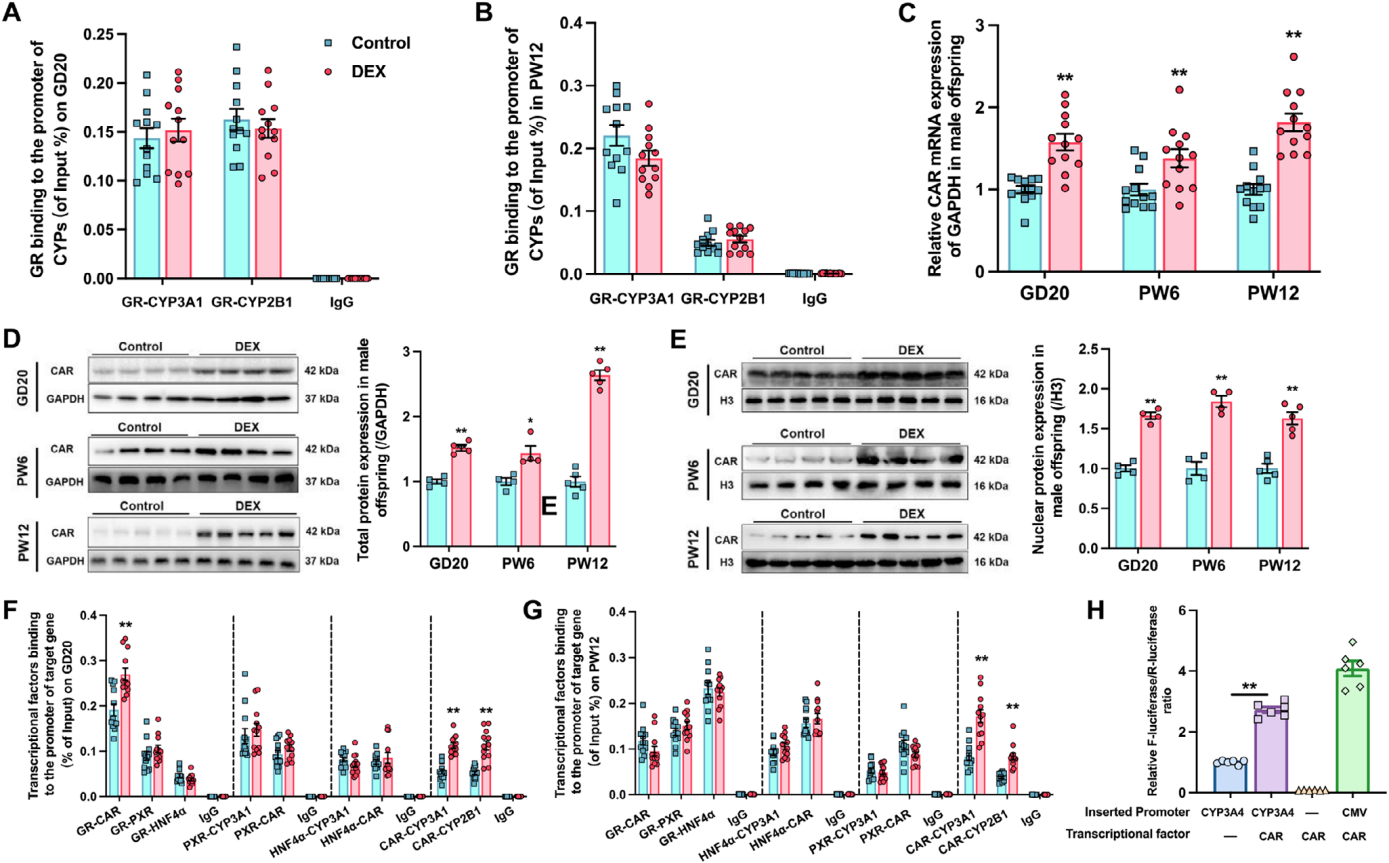
Glucocorticoid receptor (GR) indirectly mediated the intrauterine programming of cytochrome P450 3A (CYP3A) expression via constitutive androstane receptor (CAR) after prenatal dexamethasone exposure (PDE) in male offspring. ChIP‐PCR results of GR binding to the promoter of CYP3A1 and CYP2B1 in male offspring A) on gestational day (GD20) and B) in postnatal week 12 (PW12) by ChIP‐PCR, *n* = 10–12, DEX: 0.2 mg kg^−1^ d^−1^ dexamethasone in GD9‐20; C) the mRNA expression of CAR in male offspring on GD20, in PW6 and PW12, *n* = 10–12; the D) total and E) nuclear protein expression of hepatic GR in male offspring on GD20 and in PW6 and PW12, *n* = 4–5; chromatin immunoprecipitation assay with qPCR (ChIP‐PCR) results of transcription factors binding to the promoter of target gene of male offspring (F) on GD20 and (G) in PW12 by ChIP‐PCR, *n* = 10–12; H) direct binding of CAR protein to CYP3A4 promoter regions by dual‐luciferase assay. The data are presented as mean ± S.E.M., *p < 0.05, **p < 0.01 versus control.

Previous study has identified a GR response element (GRE) within the CYP3A promoter region,^[^ ^52^ ^]^ but in this study, ChIP‐PCR results didn't show the increasing binding of GR to the promoter regions of CYP3A1 and CYP2B1 (**Figure** **4**A,B), suggesting that GR activation by GC might indirectly regulate CYP transcription. Nuclear factor κB 1 (NFκB1) and AP‐1 transcription factor subunit (AP1) were used as negative control,^[^ ^53^, ^54^ ^]^ and FKBP5 was used as positive control to verify ChIP‐PCR of GR binding to GRE (Figure S3A,B, Supporting Information). We further examined the expression of other transcription factors associated with CYP3A and CYP2B expression.^[^ ^55^, ^56^, ^57^ ^]^ Results indicated a continuous increase in the nuclear receptor CAR expression from GD20 to PW12 in male offspring, whereas other transcription factors did not show persistent changes (*p* < 0.05, *p* < 0.01, Figure 4C and Figure S3C, Supporting Information). Total and nuclear protein levels of CAR were also consistently elevated in PDE male offspring (*p* < 0.05, *p* < 0.01, Figure 4D,E). Similarly, gene expression of CAR was sustained from GD20 to PW12 in male offspring in prenatal prednisolone exposure rats, while GR was induced only on GD20 (*p* < 0.05, *p* < 0.01, Figure S3D, Supporting Information). Gene expression of GR and CAR demonstrated a positive correlation on GD20 in the control and PDE groups, but not in PW12 (*p* < 0.05, *p* < 0.01, Figure S3E, Supporting Information). Additionally, we also examined the expression of multiple transcription factors and nuclear receptors in female offspring, and the results showed no significant change (Figure S3F, Supporting Information).

ChIP‐PCR results further confirmed that GR could bind to the promoter of CAR in PDE male fetal liver, but did not persist to PW12, and no binding was detected between other transcription factors and CYP3A (*p* < 0.01, Figure 4F,G). Dual‐luciferase assay confirmed the transcriptional function of GR on CAR with or without DEX treatment, and RU486 blocked transcriptional activity of GR induced by DEX (*p* < 0.01, Figure S3G,H, Supporting Information). GR overexpression enhanced CAR and CYP3A4 mRNA expression, providing further evidence for CAR and CYP3A as downstream genes of GR regulation (*p* < 0.05, *p* < 0.01, Figure S3I, Supporting Information). Furthermore, mutations of 458 in the DNA bind domain and 628 in the ligand binding domain of GR amino acid sequences could disrupt the structure of the GR dimer, resulting in the formation of a monomer.^[^ ^58^, ^59^ ^]^ We performed a dual luciferase reporter gene assay after mutating GR to a monomer and confirmed that GR dimer is the functional form for CAR regulation (*p* < 0.01, Figure S3J, Supporting Information).

ChIP‐PCR results showed that PDE promoted the binding of CAR to CYP3A1 and CYP2B1 promoter regions of in both fetal and adult male offspring (*p* < 0.01, Figure 4F,G). Dual luciferase assays also showed the transcriptional regulation of CAR on CYP3A4 (human homologous protein of CYP3A1^[^ ^60^ ^]^) in HepG2 cells (*p* < 0.01, Figure 4H). These results suggested that CAR activation directly control the transcription of CYPs. CAR needs to form a heterodimer with retinoid X receptor (RXR) and translocate into the nucleus for its transcriptional regulatory function.^[^ ^61^ ^]^ Although RXR protein expression and binding to CYP promoters was not significantly changed by PDE (Figure S3K,M, Supporting Information), the binding of CAR and RXR was increased in PDE group, which may be due to the enhanced protein level of CAR (Figure S3L, Supporting Information). These results ruled out the predominant role of RXR in PDE‐induced CYPs upregulation. Hepatic expression of phosphoenolpyruvate carboxykinase (PEPCK) and glucose‐6‐phosphatase (G6Pase), the two downstream genes negatively regulated by CAR,^[^ ^62^ ^]^ were significantly reduced from GD20 to PW12 in PDE offspring (*p* < 0.05, *p* < 0.01, Figure S3N,O, Supporting Information), supporting the inference that consistent activation of CAR was induced by PDE.

**Figure 5:**
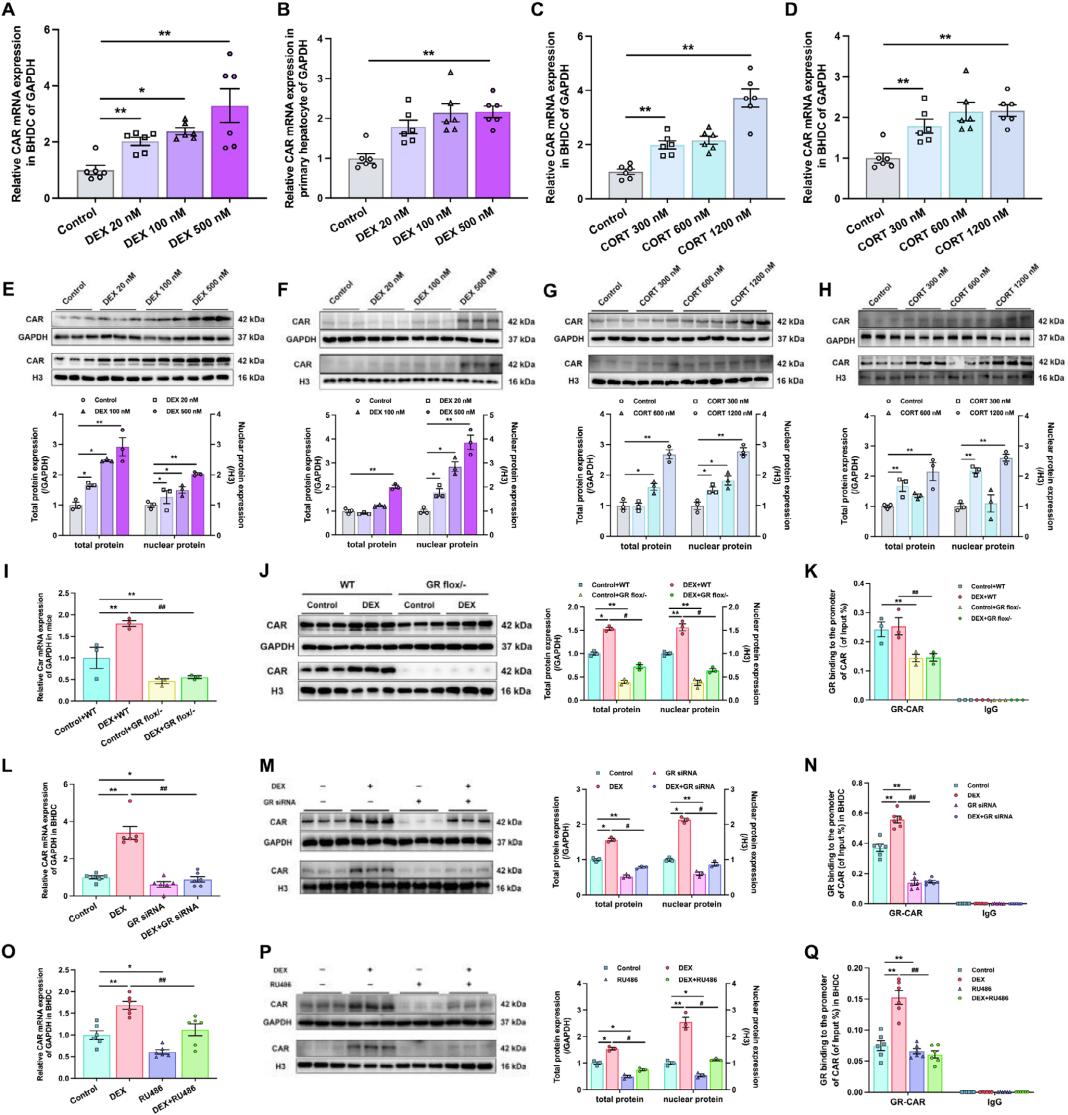
Glucocorticoid (GC) could also activate constitutive androstane receptor (CAR) in bone marrow mesenchymal stem cells hepatoid differentiated cell (BHDC). The mRNA expression of CAR in A) BHDC and B) primary hepatocyte after treatments of 0, 20 × 10^−9^, 100 × 10^−^× 10^−9^, and 500 × 10^−9^ m dexamethasone (DEX), *n* = 6; the mRNA expression of CAR in C) BHDC and D) primary hepatocyte after treatments of 0, 300 × 10^−9^, 600 × 10^−9^, and 1200 × 10^−9^ m corticosterone (CORT), *n* = 6; the total and nuclear protein expression of CAR after treatments of E) DEX and F) CORT, *n* = 3; the total and nuclear protein expression of CAR after treatments of G) DEX and H) CORT in primary hepatocyte, *n* = 6; the I) mRNA and J) total and nuclear protein expression of CAR in GR^flox/−^ (GR is glucocorticoid receptor) mice in postnatal week 12 (PW12), *n* = 3, DEX: 0.1 mg (kg^−1^ d^−1^ dexamethasone in GD9‐18; K) GR binding to the promoter of CAR in GR^flox/−^ mice in PW12 detected by ChIP‐PCR, *n* = 3; the L) mRNA and M) total and nuclear protein expression of CAR in BHDC after treatments of 500 × 10^−9^ m DEX or/and 100 × 10^−9^ m GR siRNA, *n* = 3‐6; (N) GR binding to the promoter of CAR in BHDC after treatments of 500 × 10^−9^ m DEX or/and 100 × 10^−9^ m GR siRNA detected by ChIP‐PCR, *n* = 6; the O) mRNA and P) total and nuclear protein expression of CAR in BHDC after treatments of 500 × 10^−9^ m DEX or/and 10 × 10^−6^ m RU486, *n* = 3–6; Q) GR binding to the promoter of CAR in BHDC after treatments of 500 × 10^−9^ m DEX or/and 10 × 10^−6^ m RU486 detected by ChIP‐PCR, *n* = 6. The data are presented as mean ± S.E.M., *p < 0.05, **p < 0.01 versus control, ^#^ *p* < 0.05, ^##^ *p* < 0.01 versus negative control.

Similar to GR, gene expression and nuclear translocation of CAR in both primary hepatocytes and BHDC were inducible after treatment with varying concentrations of GC in a concentration‐dependent manner (*p* < 0.05, *p* < 0.01, **Figure** **5**A–H). CAR expression in GR^flox/− Alb‐Cre/Alb‐Cre^ mice clarified the relationship between GR and CAR. PDE increased CAR expression in WT adult offspring, but regardless of DEX exposure during pregnancy, CAR expression and the binding of GR to its promoter region were decreased in the GR knockdown group (*p* < 0.05, *p* < 0.01, Figure 5I–K), indicating GR's crucial regulatory role in both constitutive and GC‐induced CAR expression. GR siRNA or RU486 treatment suppressed, CAR induction by DEX in BHDC (*p* < 0.05, *p* < 0.01, Figure 5L–Q) and other cell lines (HepG2 and LS174T), which endogenously expresses nuclear receptors and CYPs,^[^ ^63^ ^]^ to confirm the consistent activating effect of GR on CAR and downstream CYPs expression across different species and cell types (*p* < 0.05, *p* < 0.01, Figure S4A–L, Supporting Information). In summary, PDE may induce sustained high CAR expression by activating GR in utero, indirectly regulating the expression of CYP3A and CYP2B.

### 2.4 PDE Increased Acetylation Levels of H3K9 and H3K27 in CAR Promoter Region via P300/CBP

**Figure 6:**
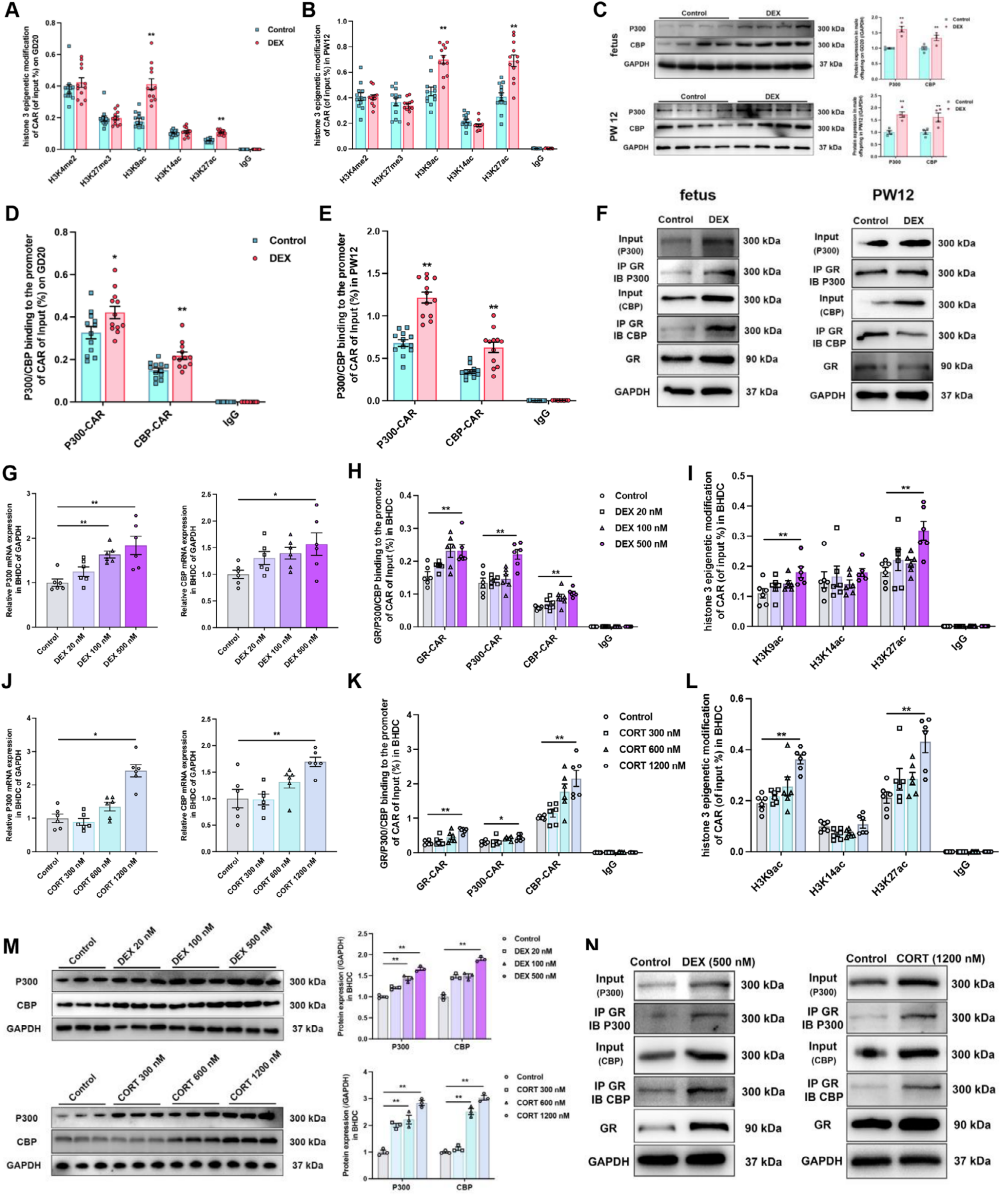
P300/CBP (cAMP response element‐binding protein binding protein) mediated the level of H3K9, and H3K27 acetylation in the constitutive androstane receptor (CAR) promoter region caused by prenatal dexamethasone exposure (PDE). Epigenetic modification in the promoter of CAR in male offspring on A) gestational day 20 (GD20) and in B) postnatal week 12 (PW12) detected by detected by ChIP‐PCR, *n* = 12; C) the protein expression and gray density value of P300 and CBP on GD20 and in PW12, *n* = 4; P300 and CBP binding to the promoter of CAR (D) on GD20 and (E) in PW12 detected by ChIP‐PCR, *n* = 12. F) Co‐immunoprecipitation of glucocorticoid receptor (GR) and P300/CBP on GD20 and in PW12, DEX: 0.2 mg kg^−1^ d^−1^ dexamethasone in GD9‐20; the G) mRNA expression of P300/CBP, H) GR, P300/CBP binding to the promoter of CAR, and I) epigenetic modification of CAR after treatments of 0, 20 × 10^−9^, 100 × 10^−9^, and 500 × 10^−9^ m DEX in bone marrow mesenchymal stem cells hepatoid differentiated cell (BHDC) detected by ChIP‐PCR, *n* = 6; the J) mRNA expression of P300/CBP, K) GR, P300/CBP binding to the promoter of CAR, and L) epigenetic modification of CAR after treatments of 0, 300 × 10^−9^, 600 × 10^−9^, and 1200 × 10^−9^ m CORT in BHDC detected by ChIP‐PCR, *n* = 6; M) protein expression of P300/CBP after treatments of 0, 20 × 10^−9^, 100 × 10^−9^, and 500 × 10^−9^ m DEX or 0, 300, 600, and 1200 × 10^−9^ m CORT in BHDC; N) detection of co‐immunoprecipitation of GR and P300/CBP after treatments of 500 × 10^−9^ m DEX or 1200 × 10^−9^ m CORT in BHDC. The data are presented as mean ± S.E.M., *p < 0.05, **p < 0.01 versus control.

Considering the programmed alterations in the expression of liver CAR, CYP3A, and CYP2B in PDE offspring and the importance of histone modifications during development,^[^ ^64^, ^65^ ^]^ we investigated altered histone epigenetic modifications on GD20 and in PW12 based on previous studies.^[^ ^66^, ^67^ ^]^ ChIP‐PCR results indicated no consistent changes in histone modification at the promoter regions of CYP3A1 and CYP2B1 (*p* < 0.05, *p* < 0.01, Figure S5A,B, Supporting Information). However, acetylation levels of H3K9 and H3K27 in the CAR promoter region consistently increased on GD20 and in PW12 (*p* < 0.05, *p* < 0.01, **Figure** **6**A,B). We further examined three acetylation sites in −2000 to 2000 bp region near the transcriptional start site (TSS) of the CAR promoter, and identified that H3K9 and H3K27 acetylation levels increased at multiple sites (*p* < 0.05, *p <* 0.01, Figure S5C,D, Supporting Information).

To identify the acetylases involved in epigenetic modification of CAR, we screened expression of various histone deacetylases (HDACs) and histone acetyltransferases (HATs) in the male offspring liver. Results showed that only the gene and protein expression of P300/CBP increased consistently, whereas other HDACs and HATs showed no consistent change in mRNA or protein expression (*p* < 0.05, *p* < 0.01, Figure S6A–C, Supporting Information, Figure 6C). Subsequent ChIP‐PCR analysis revealed the increased binding of P300/CBP to the CAR promoter region on GD20 and in PW12 (*p* < 0.05, *p* < 0.01, Figure 6D,E). Protein‐protein interaction predictions using STRING suggested that GR can form complexes with P300/CBP (Figure S6D, Supporting Information). CoIP results demonstrated a significant enhancement in the interaction between GR and P300/CBP on GD20, but with no noticeable change in PW12 (Figure 6F). These results suggested that GR may recruit P300/CBP for acetylation modification on the histones of the CAR promoter region on GD20, and the epigenetic modification potentially persisted until PW12.

DEX or CORT treatment in BHDC significantly induced gene and protein expression of P300/CBP, the binding of GR‐P300/CBP to CAR promoter, and the acetylation levels of H3K9 and H3K27, in a dose‐dependent manner (*p* < 0.05, *p* < 0.01, Figure 6G–N). Additionally, the interaction between GR and P300/CBP was significantly enhanced by treatment with 500 × 10^−9^ m DEX or 1200 × 10^−9^ m CORT. Similar results were obtained in primary rat hepatocytes (*p* < 0.05, *p* < 0.01, Figure S7, Supporting Information).

**Figure 7:**
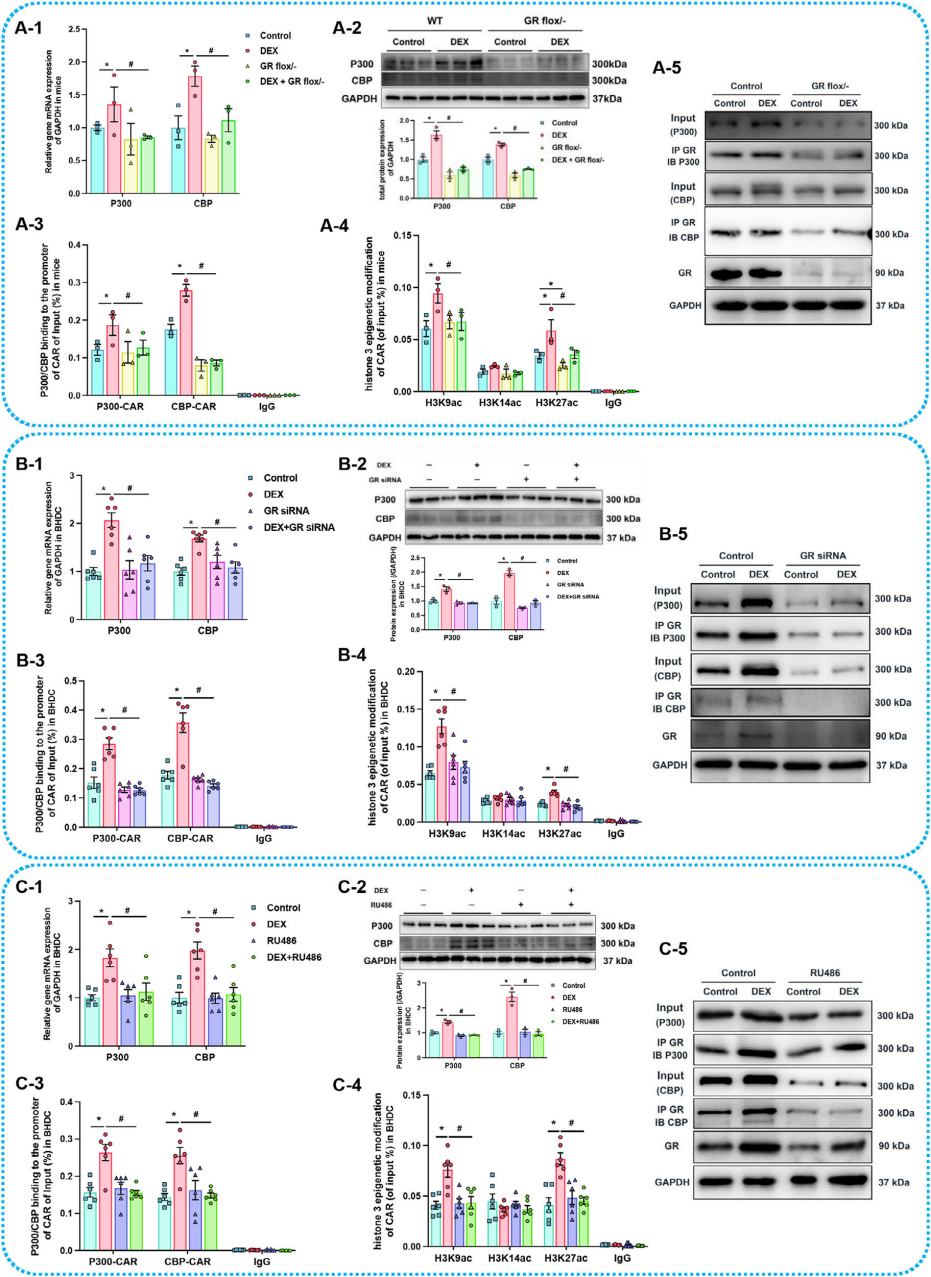
Glucocorticoid regulates epigenetic modification of constitutive androstane receptor (CAR) through glucocorticoid receptor (GR). A) The (A‐1) mRNA, (A‐2) protein expression of P300/CBP (cAMP response element‐binding protein binding protein), (A‐3) P300/CBP binding to the promoter of CAR detected by chromatin immunoprecipitation assay with qPCR (ChIP‐PCR), (A‐4) epigenetic modification of CAR, and (A‐5) detection of co‐immunoprecipitation of GR and P300/CBP in GR^flox/−^ mice in PW12, *n* = 3, DEX: 0.1 mg kg^−1^ d^−1^ dexamethasone in GD9‐18; B) the (B‐1) mRNA, (B‐2) protein expression of P300/CBP, (B‐3) P300/CBP binding to the promoter of CAR, (B‐4) epigenetic modification of CAR detected by ChIP‐PCR and (B‐5) detection of CO‐IP of GR and P300/CBP in bone marrow mesenchymal stem cells hepatoid differentiated cell (BHDC) after treatments of 500 × 10^−9^ m DEX or/and 100 × 10^−9^ m GR small interference RNA (siRNA) detected by ChIP‐PCR, *n* = 3‐6; C) the (C‐1) mRNA, (C‐2) protein expression of P300/CBP, (C‐3) P300/CBP binding to the promoter of CAR, (C‐4) epigenetic modification of CAR detected by ChIP‐PCR and (C‐5) detection of CO‐IP of GR and P300/CBP in BHDC after treatments of 500 × 10^−9^ m DEX or/and 10 × 10^−6^ m mifepristone (RU486), *n* = 3–6. The data are presented as mean ± S.E.M., *p < 0.05, **p < 0.01 versus control, ^#^ *p* < 0.05, ^##^ *p* < 0.01 versus negative control.

In WT male offspring, gene and protein expression of P300/CBP was induced by PDE, while this induction disappeared in the GR^flox/−^ group due to the GR deficiency (*p* < 0.05, *p* < 0.01, **Figure** **7**A1‐2). Furthermore, with physiological GR expression, PDE enhanced the binding of P300/CBP to the CAR promoter region and the acetylation levels of H3K9 and H3K27 in the CAR promoter region. Conversely, GR knockdown significantly reversed those effects, and the interaction between GR and P300/CBP was also diminished (*p* < 0.05, *p* < 0.01, Figure 7A3‐5). Additionally, GR siRNA and RU486 treatment in BHDC reversed the induction of P300/CBP expression, the binding of P300/CBP to the CAR promoter, and the acetylation levels of H3K9 and H3K27 by DEX (*p* < 0.05, *p* < 0.01, Figure 7B1‐5,C1‐5). Similar outcomes were observed in HepG2 and LS174T treated with GR siRNA or RU486 (*p* < 0.05, *p* < 0.01, Figure S8, Supporting Information). These results indicated that intervening in GR can reverse the epigenetic modification of CAR by PDE.

In WT male offspring, gene and protein expression of P300/CBP was induced by PDE, while this induction disappeared in the GR^flox/−^ group due to the GR deficiency (*p* < 0.05, *p* < 0.01, **Figure** **7**A1‐2). Furthermore, with physiological GR expression, PDE enhanced the binding of P300/CBP to the CAR promoter region and the acetylation levels of H3K9 and H3K27 in the CAR promoter region. Conversely, GR knockdown significantly reversed those effects, and the interaction between GR and P300/CBP was also diminished (*p* < 0.05, *p* < 0.01, Figure 7A3‐5). Additionally, GR siRNA and RU486 treatment in BHDC reversed the induction of P300/CBP expression, the binding of P300/CBP to the CAR promoter, and the acetylation levels of H3K9 and H3K27 by DEX (*p* < 0.05, *p* < 0.01, Figure 7B1‐5,C1‐5). Similar outcomes were observed in HepG2 and LS174T treated with GR siRNA or RU486 (*p* < 0.05, *p* < 0.01, Figure S8, Supporting Information). These results indicated that intervening in GR can reverse the epigenetic modification of CAR by PDE.

### 2.5 P300 and Nuclear Translocation of CAR Involved in CYP3A Induction by GCs

**Figure 8:**
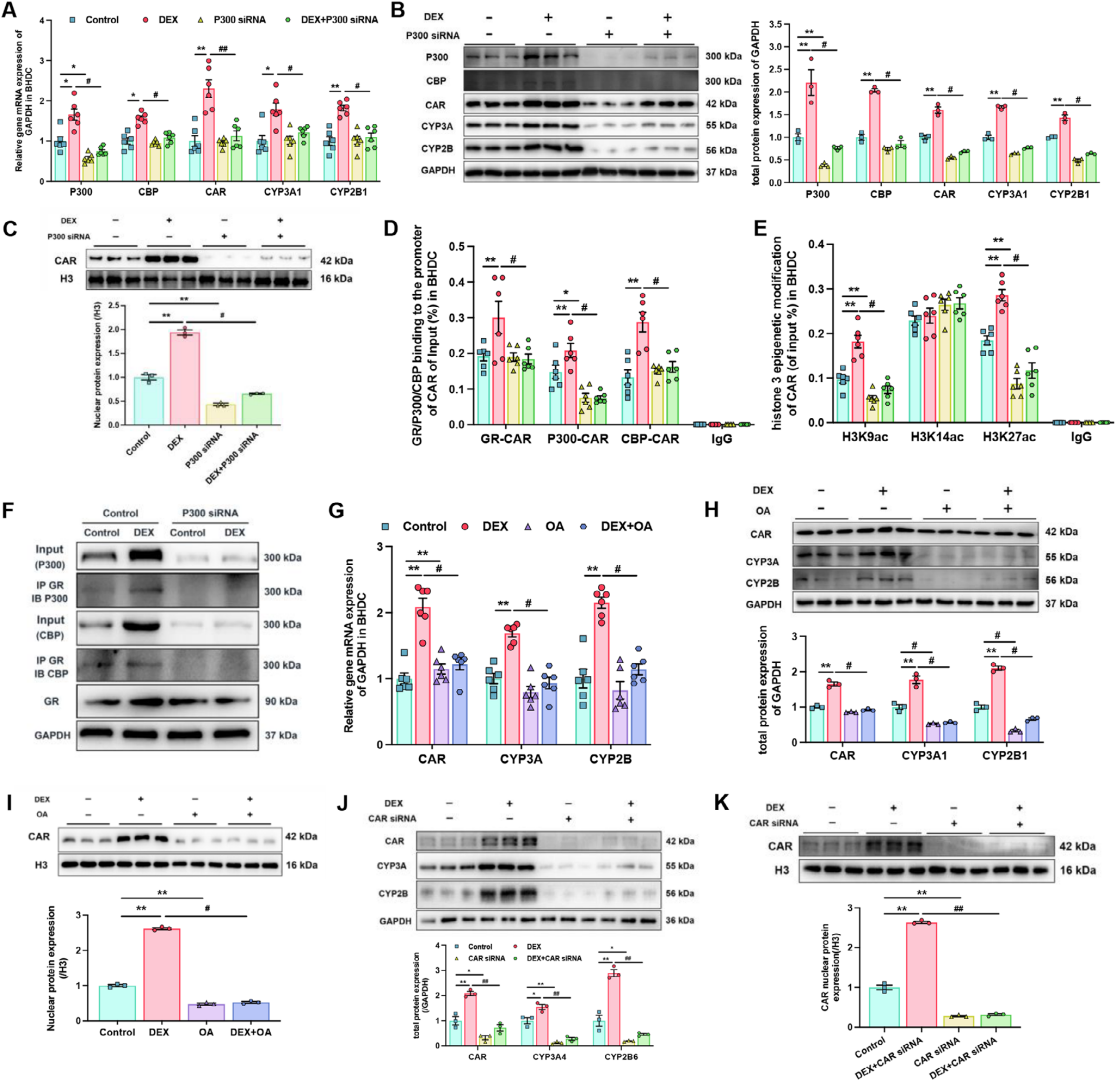
Acetylation modification and nuclear translocation of constitutive androstane receptor (CAR) are also involved in the glucocorticoid programming mechanism of cytochrome P450 3A (CYP3A). The A) mRNA of P300/CBP (cAMP response element‐binding protein binding protein), CYP3A1, CYP2B1, CAR, and B) protein expression of P300/CBP, CYP3A, CYP2B, and CAR, *n* = 3–6, C) nuclear protein expression of CAR, *n* = 3, D) glucocorticoid receptor (GR) and P300/CBP binding to the promoter of CAR detected by chromatin immunoprecipitation assay with qPCR (ChIP‐PCR), *n* = 6, E) epigenetic modification of CAR, *n* = 6, F) detection of co‐immunoprecipitation of GR and P300/CBP in bone marrow mesenchymal stem cells hepatoid differentiated cell (BHDC) after treatments of 500 × 10^−9^ m DEX or/and 100 × 10^−9^ m P300, small interference RNA (siRNA) detected by ChIP‐PCR; the G) mRNA of CYP3A1, CYP2B1, CAR, and H) total protein expression of CYP3A, CYP2B, CAR, and I) nuclear protein expression of CAR, *n* = 3–6; the J) total protein expression of CAR, CYP3A, and CYP2B, and the K) nuclear protein expression of CAR after treated with DEX or/and CAR siRNA, *n* = 3. The data are presented as mean ± S.E.M., *p < 0.05, **p < 0.01 versus control, ^#^ *p* < 0.05, ^##^ *p* < 0.01 versus negative control.

P300/CBP typically function in a dimeric form to exert acetylation modifications and are both essential.^[^ ^68^ ^]^ In this study, intervention was limited to P300 to observe the changes in downstream indicators. P300 siRNA infection reversed the induction effects of DEX on gene expression, protein binding to CAR promoter, and histone site acetylation in BHDC (*p* < 0.05, *p* < 0.01, **Figure** **8**A–F). Transcriptional activity of CAR depends on its nuclear translocation efficiency,^[^ ^69^ ^]^ while the translocation process can be inhibited by the OA through protein phosphatase 2A and activated protein kinase C1 to mediate phosphorylation of CAR protein.^[^ ^70^ ^]^ OA treatment significantly reduced the expression of downstream CYP3A and CYP2B with or without DEX incubation in BHDC (*p* < 0.05, *p* < 0.01, Figure 8G). DEX‐enhanced total and nuclear protein levels of CAR were also reversed by OA (*p* < 0.05, *p* < 0.01, Figure 8H,I). Furthermore, HepG2 transfected with CAR siRNA showed the similar results to cells treated with OA (*p* < 0.05*, p* < 0.01, Figure 8J,K).

HepG2 and LS174T were also treated with P300 siRNA and OA, combined with DEX, and similar results were obtained as in BHDC (*p* < 0.05, *p* < 0.01, Figures S9 and S10, Supporting Information). Following treatments in BDHC and primary hepatocytes, HepG2 and LS174T were treated with human endogenous CORT (hydrocortisone) to determine whether the results were consistent with DEX treatment. Results indicated that CORT could significantly induce the expression of downstream genes in a concentration‐dependent manner, consistent with DEX treatment (*p* < 0.05, *p* < 0.01, Figure S11A1,B1, Supporting Information). When treated with 1200 × 10^−9^ m hydrocortisone, related indicators and the nuclear translocation of GR and CAR significantly increased (*p* < 0.05, *p* < 0.01, Figure S11A2‐3, B2‐3, Supporting Information). Additionally, proteins binding to the CAR promoter, certain histone site acetylation, and interaction between GR and P300/CBP were significantly increased, consistent with the animal experiment results (*p* < 0.05, *p* < 0.01, Figure S11A6,B6, Supporting Information). These results suggested that GR activation by endogenous GC affect CAR and CYPs expression in the same sort of manner as DEX.

## 3 Discussion

### 3.1 GR‐Mediated Intrauterine Programming Effects of Prenatal Exposure to Excessive GC on Offspring Hepatic CYPs

GC homeostasis is critical during pregnancy for fetal development. Typically, GCs concentrations remain low but surge in the final weeks of gestation to stimulate organ differentiation and maturation, preparing the fetus for the extrauterine environment. In contrast, excessive GC signaling or prolonged synthetic GCs administration disturbs fetal development and programs functional alterations or even diseases in adult life.^[^ ^71^ ^]^ Given that various adverse prenatal factors commonly induce excessive maternal GC exposure, it is plausible that aberrant activation of GC signaling underlies the programming of hepatic CYP alterations in offspring. GCs act as a ligand to activate GR, promoting its homodimerization and translocation from the cytoplasm to the nucleus. Subsequently, GR binds to GRE within the promoter regions of target genes,^[^ ^72^ ^]^ including those encoding drug metabolism enzymes,^[^ ^73^ ^]^ thereby regulating transcription of downstream genes. Previous studies have reported that elevated intrauterine GC levels, resulting from adverse environments during pregnancy, may contribute to the CYPs induction in offspring.^[^ ^12^, ^74^, ^75^ ^]^ However, the underlying intrauterine programming mechanism remained unexplored.

In this study, PDE persistently induced the expression of hepatic CYP3A1 and CYP2B1 in male offspring rats, indicating an intrauterine programming effect. Simultaneously, GC treatment activated GR in both in vivo and in vitro experiments, as evidenced by increased GR expression level and nuclear translocation (Figure 2). However, the induction of CYP3A1 and CYP2B1 by PDE was abolished in liver‐specific GR^flox/−^ mice, a similar inhibitory effect was also observed in cell treated with GR siRNA or RU486 (Figures 2 and 3). These results demonstrated that GR plays a crucial role in GC‐induced expression of CYP3A and CYP2B. However, these results were not observed in female offspring rats. In mothers received antenatal betamethasone, placental 11β‐HSD2 activity specifically increased in female fetuses.^[^ ^76^ ^]^ Since 11β‐HSD2 is responsible for converting active cortisol into inactive cortisone, suggesting a protective effect of placenta against excess GC exposure in the female fetuses. This sexually dimorphic difference in placental 11β‐HSD2 activity may explain the observed disparity of hepatic CYP3A alteration between female and male fetuses in our study. After birth, physiological CYP3A expression in female rats is regulated by estrogen and the secretion pattern of growth hormone. CYP3A mRNA and protein expression can be suppressed by estrogen and intermittent (pulsatile) growth hormone in female rodents.^[^ ^44^, ^45^ ^]^ To avoid confounding effects of hormone‐related factors on CYP3A alteration in female offspring, mechanistic studies were conducted exclusively in males.

### 3.2 PDE Induces Continuous CYP Expression Via the GR‐CAR Pathway

Previous studies have demonstrated that GR activation not only induces CYP3A expression but also activates transcription factors associated with CYP3A regulation, including PXR, CAR, CAAT/enhancer binding protein α (C/EBPα), RXRα, and HNF4α.^[^ ^77^, ^78^, ^79^, ^80^ ^]^ Those transcription factors can even interact as a complex regulatory network,^[^ ^81^, ^82^ ^]^ which complicates the exploration of the intrauterine programming mechanisms governing offspring CYP3A expression. We screened and validated multiple candidate genes implicated in CYP3A regulation (Figure 4). Results showed that only the binding of GR to the CAR promoter region was significantly increased on GD20, consistent with previous report that the CAR promoter region contains a GRE. Moreover, DEX can increase CAR expression in a GR‐dependent manner.^[^ ^83^ ^]^ CAR activation and changes in its target genes were consistent from GD20 to PW12. However, when GR was knocked down or antagonized, DEX‐induced CAR activation and the subsequent induction of downstream CYPs were suppressed (Figure 3). CAR activation can occur through both ligand‐dependent and ligand‐independent mechanisms.^[^ ^84^ ^]^ In this study, PDE consistently induced the expression and activation of CAR in a ligand‐independent manner by continuously elevating the acetylation levels of H3K9 and H3K27 in the CAR promoter region. Additionally, inhibition of CAR nuclear translocation or expression in cell experiments further verified that CAR activation mediated GC‐induced CYP3A expression. Based on the above screening and validation results, we identified GR‐CAR pathway as the key regulatory mechanism through which intrauterine GC exposure induced hepatic CYP3A and CYP2B expression in offspring.

### 3.3 Histone Acetylation Modification in the Developmental Programming of CYPs

Exposure to adverse factors during pregnancy can alter fetal epigenetic patterns, thereby impacting developmental outcomes.^[^ ^85^ ^]^ The offspring's genome exhibited heightened sensitivity to environmental influences, with epigenetic plasticity in characteristic of early development leading to persistent alterations in gene expression profiles.^[^ ^86^ ^]^ This study found that persistent histone acetylation occurred only on CAR but not on CYP3A. PDE increased the acetylation levels of H3K9 and H3K27 within the CAR promoter region in offspring, affecting the “remodeling” of the epigenetic form of CAR histones and subsequently inducing the continuous expression of CYP3A. P300/CBP specifically catalyzes the acetylation of H3K9 and H3K27.^[^ ^87^ ^]^ In the liver of PDE offspring, enhanced interactions between GR and P300/CBP were observed exclusively on GD20. Concurrently, the binding of P300/CBP to the CAR promoter region was consistently increased both on GD20 and in PW12 (Figure 6). In vitro experiments showed that interfering with GR or P300 reversed the upregulation of CAR and CYPs induced by GC treatment (Figures 3 and 5). These results suggested that the GR and P300/CBP complex mediates alteration in histone acetylation of CAR, thereby inducing the expression of CYP3A and 2B.

### 3.4 Significance of Altered CYP3A Expression in PDE Offspring

Unlike the transient induction of CYPs by xenobiotics in adults, PDE leads to persistent alteration of hepatic CYP3A expression in offspring. Since nearly half of clinically used drugs are substrates of CYP3A, this alteration may result in accelerated drug metabolism, thereby affecting the therapeutic efficacy. Epidemiological surveys and animal experiments indicated that adverse environmental factors during pregnancy (e.g., stress, nutritional deficiency, and xenobiotic exposure) not only affect the CYP expression of offspring but also increase their susceptibility to metabolic syndromes, including fatty liver, diabetes, and hypertension.^[^ ^88^, ^89^, ^90^ ^]^ The individuals predisposed to these diseases are more likely to require medication, many of which are CYP3A substrates (e.g., nifedipine, simvastatin, troglitazone, and pioglitazone). Therefore, accelerated drug metabolism may lead to therapeutic failure or adverse drug reaction (particularly if metabolites possess greater pharmacological activity). Furthermore, we found a “memory effect” regarding the induction of CYP3A and CYP2B by DEX, although the precise mechanisms warrant further investigation. Induction of CAR, CYP3A1, and CYP2B1 in the PDE offspring was retriggered by DEX treatment in PW12, exceeding levels in the control group (*p* < 0.05, *p* < 0.01, Figure S12, Supporting Information). It could be assumed that CYP3A or CYP2B levels may significantly increase in offspring with intrauterine GR activation after GC treatment in adulthood, leading to substantial pharmacodynamic changes or drug‐drug interactions. Therefore, therapeutic drug monitoring and personalized medication are necessary for this special population, especially when administering CYP3A substrates characterized by a narrow therapeutic index, slow onset of action, or metabolic activation.

Moreover, CYP3A is involved in the biotransformation of endogenous substances. It converts cholesterol to 4β‐hydroxycholesterol in cholesterol metabolism, which is vital for maintaining physiological cholesterol homeostasis.^[^ ^91^ ^]^ In bile acid transformation, CYP3A synthesizes primary bile acids and aids in the elimination of secondary metabolites.^[^ ^92^, ^93^ ^]^ In steroid hormone metabolism, CYP3A is involved in the metabolism of testosterone, estradiol, and progesterone, producing hydroxylated products.^[^ ^8^, ^94^, ^95^ ^]^ Hepatic CYP3A4 also plays a role in the biosynthesis of arachidonic acid, an intermediate metabolite of unsaturated long‐chain fatty acids, which serves as a precursor to various bioactive molecules in the body.^[^ ^96^ ^]^ Additionally, CYP3A4 and CYP3A5 in liver catalyze the conversion of vitamin D into active hydroxylated products, which promote the absorption of calcium and phosphorus to facilitate bone formation.^[^ ^97^ ^]^ The impact of enhanced hepatic CYP3A expression in offspring on the homeostasis of endogenous substances warrants further investigation.

## 4 Conclusion

This study demonstrated that male fetuses exposed to elevated levels of GCs can activate hepatic GR. This activation facilitates the binding of P300/CBP to the CAR promoter region, resulting in enhanced acetylation at H3K9 and H3K27 sites. Consequently, transcriptional upregulation of CAR occurs and persists into adulthood. Functioning as a transcriptional regulator, activated CAR upregulates the expression of downstream CYP3A and CYP2B. These findings suggest that the GR‐CAR pathway may be a potential mechanism for programming CYP3A expression in offspring exposed to excessive intrauterine GC prenatally. This research underscores the impact of prenatal GC exposure on hepatic drug‐metabolizing enzymes in male offspring, providing experimental data and theory basis for individualized medication strategies for adults with histories of adverse intrauterine environments.

## 5 Experimental Section

### Reagents

DEX and prednisone were obtained from Qianjiang Pharmaceutical (Qianjiang, China). CYP3A (D‐2) was sourced from Santa Cruz Biotechnology (TX, USA). CYP2B, retinol x receptor α (RXRα), sirtuins 1 (Sirt1), CAR, pregnane x receptor (PXR), hepatocyte nuclear factor 4 α (HNF4α), P300, and P300/cAMP response element‐binding protein‐binding protein (CBP) antibodies, as well as okadaic acid (OA), were acquired from Abcam plc. (Cambridge, Cambridgeshire, UK). Glyceraldehyde phosphate dehydrogenase (GAPDH), CAR (for immunoprecipitation), H3K9ac, H3K14ac, H3K27ac, and lgG antibodies were purchased from Abclonal Technology Co., Ltd. (Wuhan, China). H3K4me2 and H3K27me3 antibodies were obtained from Cell Signaling Technology Co., Ltd. (Boston, MA, USA). H3 antibody was sourced from Service Biotechnology Co., Ltd. (Wuhan, China). Goat anti‐mouse IgG and goat anti‐rabbit IgG were procured from Bainiu Technology Co., Ltd. (Wuhan, China). BCA protein assay kits were obtained from EpiZyme Biotechnology Co., Ltd. (Shanghai, China). Fetal bovine serum, Dulbecco's Modified Eagle Medium (DMEM), α‐Minimum Essential Medium (αMEM), Protein Marker, lipofectamine 3000, insulin transferrin‑sodium selenite (ITS), TRIzol reagent, chromatographic grade methanol and acetonitrile, and phosphate‐buffered saline (PBS) were purchased from Thermo Fisher Scientific Inc. (Waltham, MA, USA). The oligonucleotide primers were obtained from Sangon Biotech Co., Ltd. (Shanghai, China). Real‐time quantitative polymerase chain reaction (RT‐qPCR) kits, reverse transcription kits, TaKaRa MiniBEST Universal Genomic DNA Extraction Kit, and DNA Extraction kits were purchased from Takara Biotech Co., Ltd. (Dalian, China). The restriction enzymes including *Xba*I, *Xho*I, *Not*I, and *Spe*I were purchased from New England Biolabs Biotech Co., Ltd (Beijing, China). The Dual‐Glo Luciferase Assay System was purchased from Promega Biotech Co., Ltd (Beijing, China). Proteinase K (ST533) and GR antagonist mifepristone (RU486) (ODR4395) were obtained from Kori Biotech Co., Ltd. (Wuhan, China). DNA purification kits were obtained from TIANGEN Biotech Co., Ltd. (Beijing, China). Hepatocyte growth factor (HGF) and epidermal growth factor (EGF) were obtained from Assaypro LLC. (Saint Charles, Missouri, USA). Nicotinamide adenine mononucleotide phosphate (NADPH), nifedipine (N7634, purity ≥ 98%), amlodipine (A5605, purity ≥ 98%), and nifedipine oxide (UC167, purity ≥ 95%) were acquired from Merck KGaA. (Darmstadt, Germany). Human colonic cell line LS174T (CL‐0145) was purchased from Procell Life Science Technology Co., Ltd. (Wuhan, China). GR siRNA and P300 siRNA were obtained from Gene Pharma Technology Co., Ltd. (Wuhan, China). Other reagents were of analytical grade.

### Animals Treatment

SPF Wistar rats (male: 280 ± 20 g; female: 220 ± 20 g) were acquired from Hubei Provincial Centers of Disease Control (certificate number: 42 000 600 014 526, license No. SCXK14016). The program was approved by the Animal Experiment Ethics Committee of Wuhan University Medical College (Approval No. 201 719). All animal experiments were conducted in compliance with the guidelines of Chinese Animal Welfare Committee. The animals were housed under controlled conditions at 25 ± 2 °C, with a relative humidity of 50, 10%, and a 12‐h light‐dark cycle. After a 7‐day acclimatization period, two females were mated with one male overnight (from 7 pm to 7 am). The presence of sperm in vagina was designated as gestational day (GD) 0. The pregnant rats were separately housed and treated according to reported protocol.^[^ ^98^ ^]^ The pregnant rats were randomly assigned to the control and the DEX group, each consisting of 20–24 rats. Given that rodent GR expression reaches its peak on GD9 (i.e., embryo day 10),^[^ ^99^ ^]^ the DEX group received subcutaneous injections of 0.2 or 0.8 mg kg^−1^ d^−1^ DEX daily from GD 9 to GD 20. The control group received an equivalent volume of normal saline. Two hours after the final treatment, half of the pregnant rats were euthanized under isoflurane inhalation anesthesia, and the fetal rats were delivered via cesarean section. Fetal blood and liver tissues were collected and stored at −80 °C. After weaning, three male rats from each litter were retained until PW6 and PW12. At each time point, one rat per litter was anesthetized and euthanized to collect serum and liver samples. In PW12, an additional male rat from each litter was sacrificed for liver microsome preparation. To confirm the effects of GR activation on CYP enzyme programming, another GR agonist, prednisone was administered to pregnant rats via oral gavage at doses of 0.125, 0.25, and 0.5 mg kg^−1^ d^−1^ from GD0 to GD20. Half of the offspring were sacrificed on GD20, while the remaining pups were raised until PW12. The experimental procedures followed were consistent with those used for the DEX‐treated group.

Heterozygous liver‐specific GR knockout mice [GR^flox/− Alb‐Cre/Alb‐Cre^, strain: B6.CG‐SPEer6‐PS1TG (Alb‐Cre) 21Mgn/J] were purchased from Suzhou Saiye Biotechnology Co., Ltd. (Laboratory animal license No.: SCXK [Hubei] 2019‐0004/SYXK [Hubei] 2019‐0013, protocol No. KOS140918BA1). The method used to generate the gene‐edited animals is detailed in Appendix Materials 1 and 2. After 7‐day acclimatization, two heterozygous females were mated with one heterozygous male overnight (from 7 pm to 7 am), and two wild‐type females were mated with one wild‐type male to produce male GR^flox/− Alb‐Cre/Alb‐Cre^ and wild‐type offspring. The detection of vaginal plugs was designated as GD 0. Pregnant mice were separated and randomly allocated to either the control or DEX group, with 4–6 pregnant mice in each group. Considering the dose conversion between rats and mice,^[^ ^100^, ^101^ ^]^ the DEX group mice received 0.1 mg kg^−1^ of DEX sodium phosphate from GD 9 to 18. Control mice were treated with the same volume of solvent. All pups were weaned at 3 weeks, and tail tips were collected for genotype identification (described in Section 2.3). Both wild‐type and GR^flox/− Alb‐Cre/Alb‐Cre^ mice were retained, and then sacrificed in PW12. Due to the essential role of GR in embryonic survival and the low efficiency of homozygote production by natural mating, this study utilized heterozygous liver‐specific GR knockout mice (GR^flox/− Alb‐Cre/Alb‐Cre^).

### Identification of Genotype

Mouse genomic DNA was extracted using the TaKaRa MiniBEST Universal Genomic DNA Extraction Kit (Ver.5.0_Code No. 9765) according to the manufacturer's instruction. A 2–5 mm section of the tail tip was excised and placed into a 1.5 mL microcentrifuge tube, then incubated with 180 µL of Gene Lysis (GL) buffer, 20 µL of proteinase K, and 10 µL of RNase A (10 mg mL^−1^) at 56 °C for at least 6 h. Subsequently, 200 µL Gene Balance (GB) Buffer and 200 µL 100% ethanol were added to the reaction mixture and transferred to the spin column. The DNA was washed twice by adding 500 and 700 µL of Washing A buffer, respectively, and centrifuged at 12000 rpm for 2 min each time. DNA was eluted using 50–200 µL of Elution Buffer, and centrifuged at 12000 rpm for 2 min. The concentration of extracted genomic DNA was measured using Nanodrop spectrophotometer and diluted to 100 ng µL^−1^. PCR amplification of the target gene was performed using Trans Taq DNA Polymerase High Fidelity (HIFI). The primer sequences for the target gene were as follows:

mice Nr3c1‐LoxP‐F: GCAGTCCGAGAATGGTGGCTTT,
mice Nr3c1‐LoxP‐R: CGTCAACACATGATCACCTTGCAG,
mice Nr3c1‐5′PCR‐F: CCCATGTAGAAATAGGACAGTAGAGG,
mice Nr3c1‐5′PCR‐R: GCTGACCGCTTCCTCGTGCTTTA,
mice Alb‐Cre‐F: GAAGCAGAAGCTTAGGAAGATGG,
mice Alb‐Cre‐R: TTGGCCCCTTACCATAACTG.

Gel electrophoresis was conducted on a 4% agarose gel; 10 µL of the PCR reaction DNA solution was mixed with 2 µL of 6 × loading buffer. The gel was visualized and photographed after electrophoresis at 140 V, and the genotypes of knockout mice were determined based on the band patterns obtained from different primers.

### Culture and Differentiation of Bone Marrow Mesenchymal Stem Cells (BMSCs)

Protocols for preparing BMSCs and the hepatoid‐differentiated cells (BHDCs) were based on our prior study.^[^ ^98^ ^]^ BMSCs were harvested from PW3 male rats and cultured at a density of 4 × 10^5^ cells per well in six‐well plates with growth medium (αMEM with 10% fetal bovine serum (FBS), 100 mg mL^−1^ streptomycin, and 100 U mL^−1^ penicillin). Upon reaching 80% confluence, the growth medium was replaced with hepatocyte differentiation medium (low‐glucose DMEM with 1% FBS, 100 mg mL^−1^ streptomycin, 100 U mL^−1^ penicillin, 20 ng mL^−1^ HGF, 2 ng mL^−1^ EGF, 0.1 × 10^−6^ m DEX, and 50 mg mL^−1^ ITS) for 14 days.^[^ ^102^ ^]^ Subsequently, cells were treated with varying concentrations of DEX (0, 20 × 10^−9^, 100 × 10^−9^, and 500 × 10^−9^ m) or corticosterone (0, 300, 600, and 1200 × 10^−9^ m) for 24 h, combined with 10 × 10^−6^ m GR antagonist RU486, 100 × 10^−9^ m GR siRNA, P300 siRNA, or 10 × 10^−9^ m OA, respectively. In Table S1 (Supporting Information), cycle threshold (Ct) values of liver‐specific genes Alb and Afp are presented before and after BMSC differentiation, reflecting the accomplishment of hepatoid differentiation.

### Total RNA Extraction and RT‐qPCR

Cells and liver samples were subjected to RNA isolation using a TRIZOL reagent. Process of RNA extraction and cDNA preparation was according to our prior work.^[^ ^103^ ^]^ qPCR was performed using the Cham Q TM Universal SYBR® qPCR Master Mix on the QuantStudio®5 Real‐Time PCR System (Thermo Scientific, Waltham, MA, USA) for detection. All primers are listed in Table S2 (Supporting Information). Target gene expression was standardized using housekeeping gene and computed using the 2^−ΔΔ^Ct methodology. For the gene expression levels assayed in each tissue of fetal mice, *n* indicates a litter (with each *n* ranging from 11‐12).

### Western Blotting

Total protein was extracted from the liver and cells using RIPA lysis buffer, and protein concentration was determined using a bicinchoninic acid (BCA) assay. Nuclear proteins were extracted using the Nuclear Protein Extraction Kit (no. P0028, Beyotime Institute of Biotechnology, Shanghai, China).^[^ ^104^ ^]^ To verify the purity of extracted cellular fractions, we examined the levels of cytoplasmic localization proteins (ACTB and GAPDH) and nuclear localization protein (H3) in cytoplasmic and nuclear samples, respectively. The results showed that ACTB and GAPDH were enriched in cytoplasmic samples, while H3 was predominantly detected in the nuclear fraction (Figure S2A, Supporting Information). Details of the western blot procedure have been described previously.^[^ ^105^ ^]^ The primary antibodies used were as follows: rabbit‐anti CYP3A antibody (1:250), CYP2B (1:5000), CAR (1:1000), GR (1:2000), Sirt1 (1:500), RXR (1:500), P300 (1:100), and CBP (1:500) incubated overnight at 4 °C. Subsequently, the HRP‐conjugated secondary antibody (1:5000) was incubated and visualized by chemiluminescence using an electro‐chemiluminescent (ECL) detection kit (Zhongshan Golden Bridge Biotechnology Co. Ltd., Beijing, China). Results were quantified using Image J Launcher.

### Culture and Treatment of Cell Lines

LS174T, derived from human colon cancer cells, is frequently used in CYP studies due to its robust CYP expression and induction ability.^[^ ^63^ ^]^ In this study, HepG2 (No. CL‐0103, RRID: CVCL_D5I8) and LS174T (No. CL‐0145, RRID: CVCL_1384) were both purchased from Wuhan Pricella Biotechnology Co., Ltd. (Wuhan, China). According to the Cellosaurus database (https://www.cellosaurus.org), both HepG2 and LS174T cell lines were confirmed to be contamination free. HepG2 and LS174T were cultured in DMEM or αMEM medium (pH 7.2–7.4) containing 10% FBS, 1% penicillin, and streptomycin at 37 °C and 5% CO_2_. Referring to our previous work,^[^ ^106^ ^]^ HepG2 and LS174T cells were treated with gradient concentrations of DEX (20, 100, and predominantly 500 × 10^−9^ m), corticosterone (300, 600, and predominantly 1200 × 10^−9^ m), 10 × 10^−6^ m RU486, 100 × 10^−9^ m GR siRNA, P300 siRNA, CAR siRNA, or 10 × 10^−9^ m OA. Cells were harvested for total RNA extraction (*n* = 6) or protein extraction (*n* = 3) for subsequent experiments. The specific sequences are shown in Table S3 (Supporting Information).

### Chromatin Immunoprecipitation (ChIP) Assay

Cells were cross‐linked with 1% formaldehyde at room temperature, and glycine (final concentration 125 × 10^−3^ m) was added to terminate the reaction.^[^ ^105^ ^]^ The suspensions were sonicated to obtain 200‐800 bp DNA fragments, and then were incubated with Agarose‐protein A/G beads and divided into multiple portions. Corresponding antibodies (H3K9ac, H3K14ac, H3K27ac, H3K4me2, H3K27me3, GR, and CAR) and IgG were added and shaken overnight at 4 °C for immunoprecipitation, followed by the addition of 200 µg mL^−1^ proteinase K at 65 °C overnight. DNA was isolated from each immunoprecipitation and subjected to RT‐qPCR analysis. The Jaspar database was consulted to query gene transcription binding sites.^[^ ^107^ ^]^ Primers for amplification of multiple regions of the CAR promoter were designed using the UCSC Genome Browser (http://genome.ucsc.edu/) and Primer 3 Input Browser (http://primer3.ut.ee/). The primer sequences are listed in Table S4 (Supporting Information). Data from RT‐qPCR were used to calculate the abundance of proteins enriched in the promoters of each gene using the following formula: IP/input = 100 × 2^Ct input DNA – Ct IP DNA^.

### Co‐immunoprecipitation (CoIP) Assay

Tissue and cell lysates were collected, and protein concentrations were quantified using a BCA protein quantification kit. Protein A agarose gel beads (20 to 40 µL) were washed 2 to 3 times with lysis buffer and centrifuged at 3000 rpm for 5 min each time. Between 500 and 1000 µL of homogenized lysis supernatant from tissues or cells were transferred to a clean EP tube, and 1–10 µL of specific primary antibody was added to incubate overnight at 4 °C. IgG from the same species served as a negative control. Subsequently, 20 µL of A/G agarose gel beads were added, and the mixture was incubated for 3‐4 h at 4 °C. After incubation, the sample was centrifuged at 1000 g for 5 min at 4 °C to collect the immunoprecipitated complexes; the mixture was then washed 3‐4 times with 1 mL ice‐cold IP lysate (without protease inhibitor) by centrifugation at 3000 g for 5 min. Following the final wash, 40 µL of 1 × SDS loading buffer containing mercaptoethanol was added, boiled for 10 min at 100 °C, centrifuged at 1000 g for 5 min at 4 °C, and 15–20 µL of supernatant was used for WB assay.

### Culture of Rat Primary Hepatocytes

Hepatocytes were isolated by a two‐step perfusion method:^[^ ^108^ ^]^ the liver was separated from newborn rats and placed in a beaker containing Collagenase I solution for fragmentation and digestion. The filtrate was centrifuged at 4 °C for 5 min at 50 g. The precipitate was washed with Williams E culture solution and centrifuged again, and the supernatant was discarded to obtain liver parenchymal cells. 25 mL Williams E culture medium was used to resuspend the hepatocytes and re‐inoculate them into 6‐well plates.

### Detection of Enzyme Kinetics of Liver Microsomes

Rat liver microsomes were prepared according to a previous study.^[^ ^109^ ^]^ The Methodology validation of HPLC was shown in Appendix Materials 3. The liver was homogenized in 0.05 M Tris‐HCl buffer (containing 1.15% KCl, pH = 7.4, w/v = 1 g per 4 mL). Then serial centrifugation was performed at 4 °C (200 *g* × 10 min and 9000 *g* × 20 min to obtain supernatant, 105 000 *g* × 60 min for precipitate). The precipitate was resuspended in 0.25 M sucrose solution, and protein concentration was determined using a BCA kit. The reaction system contained 0.125 mL reaction buffer (200 mmol L^−1^ K_2_HPO_4_/KH_2_PO_4_, pH 7.4), 0.025 mL liver microsomes (10 mg mL^−1^), 0.01 mL MgCl_2_ (100 mmol L^−1^), 0.025 mL nifedipine (40 mmol L^−1^), and 0.005 mL NADPH (50 mmol L^−1^). After 60 min incubation at 37 °C, 5 µL of 0.2 × 10^−3^ m internal standard amlodipine and 1.2 mL (tert‐butyl methyl ether: isooctane = 3:1) were added to terminate the reaction. Liquid chromatographic conditions were as described in previous studies:^[^ ^110^, ^111^ ^]^ mobile phase: A was methanol, B was 0.03% triethylamine water (pH 4.0), A: B = 65: 35; flow rate was 1 mL min^−1^; detection wavelengths were 238 nm and 360 nm for amlodipine and nifedipine oxide; column temperature was 25 °C. The limit of detection for nifedipine oxide was 0.5 µmol L^−1^.

### In Vivo Pharmacokinetics Study

Male rats in both control and PDE groups underwent jugular vein catheterization in PW12, followed by oral administration of nifedipine (10 mg kg^−1^, in 0.5 mL vehicle solution) via gavage on the subsequent day after 24‐h recovery period. Samples collection and preparation referred to reported study.^[^ ^112^ ^]^ About 0.2 mL of blood were collected from the vein at different time points (0, 15, 30, 45, 60, 120, 240, 480, 720 and 1440 min). The plasma samples were collected in heparinized centrifuge tubes and separated by immediately centrifugation at 13 000 rpm for 10 min. 25 µL of 100 ng mL^−1^ amlodipine and 200 µL acetonitrile were added in each sample. The mixture was vortex‐mixed and subsequently analyzed on a high‐performance liquid chromatography‐tandem mass spectrometry (HPLC‐MS) equipped with an electrospray ionization (ESI) source.^[^ ^113^ ^]^ The analytes were separated with a Hypersil Gold C18 column (50 mm×2.1 mm×3 µm). The process details and methodology validation was shown in Appendix Materials 3. Pharmacokinetic parameters were calculated by DAS version 2.0 with the non‐compartment model.

### Plasmids Construction and Dual‐Luciferase Assay

The dual‐luciferase reporter plasmid of pTol2‐cmv‐Fluc‐bGH polyA‐EF1α‐Rluc‐polyA was synthesized by Tsingke company and worked as a positive control. Overlapping PCR was performed to generate insert of the target gene promoter base on HepG2 genomic DNA template. Employing Hifi assembly kit, promoter fragments were inserted into SpeI/NtoI site of reporter vector, and coding sequences were inserted into XbaI/XhoI site of pTol2‐EGFP‐polyA plasmid. 48 h after transfection, cells were collected for dual‐luciferase assay according to Promega luciferase kit instructions. The primers for overlap PCR are listed in Table S5 (Supporting Information).

### Statistical Analysis

All experimental data were analyzed using IBM SPSS Statistics 20 (SPSS Science Inc, Chicago, IL, USA) and GraphPad Prism 10.1.1 (GraphPad Software, La Jolla, CA, USA). Measured parameters were reported as the mean value and the standard error of the mean (S.E.M.). Statistical studies compared the test article groups with the control group. Due to the relatively small sample size (*n* = 11‐12), all data were tested for normality using the Shapiro‐Wilk test and for homogeneity‐of‐variance using the Levene test, and then differences between the two groups were compared using the Student's *t*‐test, while multiple group comparisons were made by one‐way analysis of variance (ANOVA) followed by Dunnett's *t* or Bonferroni's post hoc tests. A logarithmic adjustment was performed before further testing if the data failed to meet the normal distribution or variance homogeneity criteria. If the data transformation did not work, the Kruskal‐Wallis H test, followed by the Mann‐Whitney U test (equivalent to Wilcoxon W), would be used for group‐to‐group comparison. *P* values less than 0.05 (two‐tailed) were deemed statistically significant.

## Author Contributions

X.S. and J.L. performed conceptualization, data curation, formal analysis, visualization, wrote the original draft, and reviewed and edited the writing. E.X., X.L., X.Y., and Y.W. performed data curation. F.L. performed supervision. H.K. and H.W. helped in supervision, project administration, and reviewed and edited the writing. Y.G. performed conceptualization, data curation, formal analysis, funding acquisition, visualization, and reviewed and edited the writing.

## Conflict of Interest

The authors declare no conflict of interest.

## Supporting information



Supporting Information

Supporting Information

Supporting Information

Supporting Information

Supporting Information

## Data Availability

The data that support the findings of this study are available from the corresponding author upon reasonable request.
