# Supplementary material for: The Pivotal Role of GR‐CAR Pathway in Fetal Programming of Hepatic Cytochrome P450 3A Alteration in Adulthood
Source: Adv Sci (Weinh). 2025 Nov 16;13(6):e15583. doi: 10.1002/advs.202515583 (PMC12866823; doi:10.1002/advs.202515583)
Supplement: Supplementary file 1 — Supporting Information [file ADVS-13-e15583-s002.docx]

**Supporting information**

**The pivotal role of GR-CAR pathway in fetal programming of hepatic cytochrome P450 3A alteration in adulthood**

Xiaoxiang Sun^1, 3, #^, Jie Liu^1, 6, #^, E Xiang^1^, Xia Li^1^, Xuerong Yan^1^, Yuxi Wang^1^, Feng Li^4^, Hao Kou^2, 5,*^, Hui Wang^1, 2,*^, Yu Guo^1, 2,*^

*^1^Department of Pharmacology, School of Basic Medical Sciences, Wuhan University, Wuhan 430071, China;*

*^2^Hubei Provincial Key Laboratory of Developmentally Originated Disease, Wuhan 430071, China;*

*^3^Department of Pharmacy, Union Hospital, Tongji Medical College, Huazhong University of Science and Technology, Wuhan 430022, China;*

*^4^Department of Medical Genetics, School of Basic Medical Sciences, Wuhan University, Wuhan 430071, China;*

*^5^Department of Pharmacy, Zhongnan Hospital of Wuhan University, Wuhan 430060, China;*

*^6^School of Medicine, Jingchu University of Technology, Jingmen, 448000, China.*

**Contents**

**Table S1.** Ct values of BMSCs before and after differentiation.

**Table S2.** Oligonucleotide primers and PCR conditions in quantitative real-time PCR.

**Table S3.** The siRNA sequences in various species.

**Table S4.** Primers for Chromatin immunoprecipitation–polymerase chain reaction.

**Table S5.** Primers for overlap PCR in plasmids construction.

**Table S6.** The main parameters of nifedipine in plasma after intragastrical administration to male rats of PDE and control group on PW12.

**Figure S1.** Effects of PDE on CYPs expression of offspring.

**Figure S2.** Identification results related to the construction of heterozygote of liver-specific GR knockout mice (genotype: GR^flox/- Alb-Cre/Alb-Cre^).

**Figure S3.** Effects of PDE on nuclear receptors expression of offspring.

**Figure S4.** GR induced the regulation of CYP3A expression by glucocorticoids was confirmed using HepG2 and LS174T.

**Figure S5.** High acetylation levels in multiple regions of the CAR promoter region.

**Figure S6.** P300/CBP is involved in the continuous epigenetic modification of CAR.

**Figure S7.** Glucocorticoids increase the acetylation level of CAR promoter region via P300/CBP in primary hepatocyte.

**Figure S8.** GR was found to be critical for P300/CBP-mediated epigenetic modification of the CAR promoter region in HepG2 and LS174T.

**Figure S9.** Confirmation of the important role of P300 and CAR for altered glucocorticoid programming CYP3A expression in HepG2.

**Figure S10.** Confirmation of the important role of P300 and CAR for altered glucocorticoid programming CYP3A expression in LS174T.

**Figure S11.** Endogenous glucocorticoid-hydrocortisone was confirmed to program expression of CYP3A through GR and CAR in HepG2 and LS174T.

**Figure S12.** DEX has an induced memory effect on CYP3A expression in adult offspring exposed to DEX during pregnancy.

**Appendix material 1.** Vector Report for mNr3c1_Conditional Knockout Project.

**Appendix material 2.** ES Cell Report for Conditional Knockout mNr3c1 Project.

**Appendix material 3.** Methodology validation of HPLC for Detection of enzyme kinetics of liver microsomes.

**Western blot original image.**

**Table S1. Ct values of BMSCs before and after differentiation.**

| Target gene | Ct values | |
| --- | --- | --- |
|  | Before differentiation | After differentiation |
| ALB | 37.671478271 | 27.010599136 |
| ALB | Undetermined | 26.765218734 |
| ALB | Undetermined | 26.882610321 |
| ALB | 36.073886871 | 27.439973831 |
| ALB | Undetermined | 27.552754872 |
| ALB | Undetermined | 27.596449851 |
| AFP | 37.468637466 | 26.743217468 |
| AFP | Undetermined | 26.891395568 |
| AFP | Undetermined | 26.987274169 |
| AFP | Undetermined | 27.128360748 |
| AFP | Undetermined | 26.968962864 |
| AFP | Undetermined | 26.551946640 |
| GAPDH | 17.782693749 | 17.456892343 |
| GAPDH | 17.279093207 | 18.138682394 |
| GAPDH | 18.034807093 | 17.978698263 |
| GAPDH | 17.578790725 | 18.278463209 |
| GAPDH | 17.568935845 | 17.899789298 |
| GAPDH | 18.268953268 | 17.478956386 |

Ct, cycle threshold; ALB, albumin; AFP, alpha fetoprotein; GAPDH, glyceraldehyde phosphate dehydrogenase.

**Table S2.** **Oligonucleotide primers and PCR conditions in quantitative real-time PCR**

| Genes | Forward primer | Reverse primer | Annealing |
| --- | --- | --- | --- |
| Rat-CYP3A1 | TCTAAGGATGAGGAGTGGAAGA | CTCCATACTGTTCGATGACAGG | 60°C,60 s |
| Rat-CYP2B1 | CACACAGAGTCACCAAAGAC | CCAGGAAGTGTTCAGGATTG | 60°C,60 s |
| Rat-CYP2C11 | GGACATCGGCCAATCAATAA | CATTGCATCCCATTGCTAAA | 60°C,60 s |
| Rat-CYP2D1 | ATTCCTTCAAAAGCCTGCAA | TGGCATGTTCTCAAAGTCCA | 60°C,60 s |
| Rat-CYP2D2 | ATTCCTTCAAAAGCCTGCAA | TGGCATGTTCTCAAAGTCCA | 60°C,60 s |
| Rat-CYP2E1 | GACCCCACATTTCTGATTG | TTCCAGGTAGGTATCGTAG | 60°C,60 s |
| Rat-CAR | GAGCCACGGGCTATCATTTC | CTCCCAGCAAACGGACAGAT | 63°C,60 s |
| Rat-PXR | CTTCGCCAAAGTCATATCCCA | TGTTTCCGTGTCGAACATCG | 63°C,60 s |
| Rat-HNF4α | CGACATGGACATGGCTGACT | GCTTGAGGCTCCGTAGTGTT | 60℃, 60 s |
| Rat-GR | GTGAAATGGGCAAAGGCG AT | ATCTCCAACCCAGGG CAAAT | 60℃, 60 s |
| Rat-C/EBPα | CCTCACTTGCAGTTCCAGAT | CGGTACTCGTTGCTGTTCTTA | 62℃, 62 s |
| Rat-GAPDH | GCAAGTTCAACGGCACAG | GCCAGTAGACTCCACGACA | 60℃, 60 s |
| Rat-HDAC1 | TGCTGGACTTACGAGACAGC | GGAAGGGCTGATGTGAAGC | 60℃, 60 s |
| Rat-HDAC2 | CTGTCAAAGGTCACGCTAAA | CAGTCTCATACGTCCAACATC | 60℃, 60 s |
| Rat-HDAC3 | AGGTGGTGGACTTCTATCAG | CACCAGGAGAGGGATATTGA | 60℃, 60 s |
| Rat-HDAC4 | CTACATCAGAGACCCAATGC | GTGACTGTCTCAGCTTCTTC | 60℃, 60 s |
| Rat-HDAC5 | CCGTGCTCTACATCTCTTTG | GCTGTCAGGTATTCCACATC | 60℃, 60 s |
| Rat-HDAC6 | AGGGAACTACACTGGATCTG | GGCATTTGAGGATGGAGAAG | 60℃, 60 s |
| Rat-HDAC7 | TACAGAACTCTTGAGCCCTT | CAGGGATTTCTTGGGTTTGT | 60℃, 60 s |
| Rat-HDAC8 | GGCAAGTGTCTGAAGTATGT | TGGGATCTCAGAGGATAGTG | 60℃, 60 s |
| Rat-HADC9 | CCCAGCATCCTGTACATTTC | GCTTCAAGGTACTCAACATCTC | 60℃, 60 s |
| Rat-HDAC10 | CTTCACAAGTCCCAGTTTCA | ATTCTCCTCTGACCTCTATGG | 60℃, 60 s |
| Rat-HDAC11 | CTTACTTCCTCCCTTCAGTCT | CCTTCTTCAGACCTCCAAATC | 60℃, 60 s |
| Rat-sirt 1 | AGGGAACCTCTGCCTCATCTAC | TTGGCATACTCGCCACCTAAC | 60℃, 60 s |
| Rat-sirt 2 | CTGCCAGCAAGGTTCTTACTAC | TTCCACACCCTGGACTACAT | 60℃, 60 s |
| Rat-sirt 6 | CCCAAGTGTAAGACGCAGTA | CAGTCCAGAATGGTGTCTCTC | 60℃, 60 s |
| Rat-Gcn5 | CCCTGACTACTACGAGGTTAT | GCGACAATATTCGCTGTCT | 60℃, 60 s |
| Rat-PCAF | ACCAGTGAAGCGAACAGAAG | GCTGTAAGTCCGCCATGAATA | 60℃, 60 s |
| Rat-SRC1 | GAAGCCTTCACTGGGTACTATG | GTGCAGCTGGAGGTCTATTT | 60℃, 60 s |
| Rat-P300 | CAGGCAATGGACAAGGGATAA | CATTTGGGTACTGCATGTTTGG | 60℃, 60 s |
| Rat-CBP | CCAATGACTCCAGGACCTTATAC | TCTTGATACACCGAAGCATCTC | 60℃, 60 s |
| Rat-ALB | AAGGCACCCCGATTACTCCG | TGCGAAGTCACCCATCACCG | 60℃, 60 s |
| Rat-AFP | GCTGAACCCAGAGTACTGAC | GACACGTCGTAGATGAACGTG | 60℃, 60 s |
| Rat-Fkbp5 | CGACAAGGAGGGAAGAGA | CTGTGGTTGGTTGGGAAG | 60℃, 60 s |
| Rat-PER3 | CCTGTTTCCACGTTCCCTTC | TGTGTGTCTCCCAGTTCTCC | 60℃, 60 s |
| Mouse-Tsc22d3 | CATGGAGGTGGCGGTCTATC | CACCTCCTCTCTCACAGCGT | 60℃, 60 s |
| Mouse-Sgk1 | GAGAAGGATGGGCCTGAACGAT | CGGACCCAGGTTGATTTGTTGA | 60℃, 60 s |
| Mouse-Tat | GTGGAATTCACAGAGCGG | GCCTATTTGTCACACTCC | 60℃, 60 s |
| Mouse-Mt1 | CTCCTGCAAGAAGAGCTGCTG | GCGCTGTTCGTCACATCAGG | 60℃, 60 s |
| Mouse-Alb | GAAGAAAGCCCACTGTCTTAGT | GTCTCAGCAACAGGGATACAG | 60℃, 60 s |
| Mouse-Hnf4α | GAAGGTGCCAACCTCAATTC | CCACACATTGTCGGCTAAAC | 60℃, 60 s |
| Mouse-Pcna | CACGTATATGCCGAGACCTTAG | GTTACCGCCTCCTCTTCTTTAT | 60℃, 60 s |
| Mouse-Ki67 | CAGCAGATGGAACTAGGCTTAC | AGTAGCGTGATGTTTGGAAGAG | 60℃, 60 s |
| Mouse-Caspase3 | TGGTTCATCCAGTCCCTTTG | TCGTGAGCATGGACACAATAC | 60℃, 60 s |
| Mouse-Pepck | AGCCTTTGGTCAACAACTGG | TGCCTTCGGGGTTAGTTATG | 60℃, 60 s |
| Mouse-G6pase | CCTCGTCTTCAAGTGGATTCTG | GGTGACAGGGAACTGCTTTAT | 60℃, 60 s |
| Mouse-Srebp1c | GGAGCCATGGATTGCACATT | CAGGAAGGCTTCCAGAGAGG | 60℃, 60 s |
| Mouse-Fasn | CGTGTGACCGCCATCTATAT | GGTTGCTGTCGTCTGTAGTC | 60℃, 60 s |
| Mouse-Cyp3a11 | ACAAACAAGCAGGGATGGAC | GGTAGAGGAGCACCAAGCTG | 60℃, 60 s |
| Mouse-Cyp2b10 | AAGGAGAAGTCCAACCAGCA | CTCTGCAACATGGGGGTACT | 60℃, 60 s |
| Mouse-Car | CTCAAGGAAAGCAGGGTCAG | AGTTCCTCGGCCCATATTCT | 60℃, 60 s |
| Mouse-Gr | CCTGCATGTATGACCAATGT | TGAGGAGAGAAGCAGTAAGG | 60℃, 60 s |
| Mouse-P300 | CAACATGACACCCTACTTACC | CAAATTCAGAGGGCAGCA | 60℃, 60 s |
| Mouse-Cbp | CGCAAGACTAATGGAGGATG | GGGCACAGGGCATTTATT | 60℃, 60 s |
| Mouse-Gapdh | TGTGTCCGTCGTGGATCTGA | TTGCTGTTGAAGTCGCAGGAG | 60℃, 60 s |
| Human-CYP3A4 | GCCTGGTGCTCCTCTATCTA | GGCTGTTGACCATCATAAAAG | 60°C,60 s |
| Human-CYP3A7 | AAGTCTGGGGTATTTATGACT | CGCTGGTGAATGTTGGAGAC | 60°C,60 s |
| Human-CYP2B6 | AAACCAGACGCCTTCAATC | GACAAATCCGCTTCCCTAAG | 60°C,60 s |
| Human-PXR | ACAGCTGGCTAGCATTCCTC | CTTGCCTCTCTGATGGTCCTG | 60°C,60 s |
| Human-CAR | CATTGCGGCGAGCAAAGCAG | TGGGCAGGGAACGGAAGACG | 60°C,60 s |
| Human-GR | TTTCTTATGGCATTTGCTCTGG | CAACAATCTTGGCGCTCAAAA | 60°C,60 s |
| Human-CBP | GGCCCTTACATTCACAGATAG | GCCAGATGGTGGTCTTATTT | 60°C,60 s |
| Human-P300 | GGCTGTATCAGAGCGTATTG | TCTTCCTCCTGTTCCAGTT | 60°C,60 s |
| Human-GAPDH | CCCATCACCATCTTCCAGGAG | GTTGTCATGGATGACCTTGGC | 60°C,60 s |

CYP, cytochrome P450; CAR, constitutive androstane receptor; PXR, pregnane X receptor; HNF4α, hepatic nuclear factor 4 alpha; GR, glucocorticoid receptor; C/EBPα, CAAT/enhancer binding protein alpha; GAPDH, glyceraldehyde phosphate dehydrogenase; HDAC, histone deacetylases; Sirt, sirtuins; FKBP5, FK506 binding protein 5; PER3, period circadian regulator 3; Tsc22d3, transforming growth factor β-inducible gene 22 domain family protein 3; Sgk1, serum-and glucocorticoid-inducible kinase 1; Tat, tyrosine aminotransferase; Mt1, melatonin receptor; Alb, albumin; Hnf4α, hepatocyte nuclear factor 4α; Pcna, proliferating cell nuclear antigen; Pepck, phosphoenolpyruvate carboxykinase; G6Pase, glucose-6-phosphatase; Srebp1c, sterol regulatory element binding protein 1c; Fasn, fatty acid synthetase; ALB, albumin; AFP, alpha fetoprotein.

**Table S3. The siRNA sequences in various species**

| **Target genes** | **Species** | **sense sequence** | **antisense sequence** |
| --- | --- | --- | --- |
| GR siNRA | Human | GAUGAACCUGAGGGAUGAUTT | AUCAUCCCUCAGGUUCAUCTT |
| Negative control | Human | UUCUCCGAACGUGUCACGUTT | ACGUGACACGUUCGGAGAATT |
| GR siNRA | Rat | CCUGACAGAUGGUAUCAUUTT | AAUGAUACCAUCUGUCAGGTT |
| Negative control | Rat | UUCUCCGAACGUGUCACGUTT | ACGUGACACGUUCGGAGAATT |
| P300 siRNA | Human | CCAUAAAGUCACCCAAUAUTT | AUAUUGGGUGACUUUAUGGTT |
| Negative control | Human | UGUCCCGAACGUGUCACCUTT | ACGAGGCACGUUCGGCUCATT |
| P300 siRNA | Rat | CCGCGAGGCGCUAUUTT | AAUAGCCAAUGCCUCGCGGTT |
| Negative control | Rat | GGGCCUCCAAGGAAACAAATT | UUUGUUUCCUUGGAGGCCCTT |
| CAR siRNA | Human | CCACAGGCUACCACUUUAATT | UUAAAGUGGUAGCCUGUGGTT |
| Negative control | Human | CGGCUUGCAAGGCCAAUGATT | UCAUUGGCCUUGCAAGCCGTT |
| CAR siRNA | Rat | GGAGCUAUUUGCUGAAGAATT | UUCUUCAGCAAAUAGCUCCTT |
| Negative control | Rat | GCAGCCGUCAGCCAAAUGUTT | ACAUUUGGCUGACGGCUGCTT |

GR, glucocorticoid receptor; CAR, constitutive androstane receptor.

**Table S4. Primers for Chromatin immunoprecipitation–polymerase chain reaction**

| **Genes** | **Forward primer** | **Reverse primer** | **Annealing** |
| --- | --- | --- | --- |
| Rat-CYP3A1 | GCTATTCTGGCCCAAGATGC | CTGCTCTCGTTGATCCTCCT | 62°C,60 s |
| Rat-CYP2B1 | TGCCATCTCACAACTGCCTA | AGGAGAGATGGCAGGCTTTT | 62°C,60 s |
| Rat-CAR | TCTGTAGTTTGGGGCTGGAG | ATTGAACTCGGGACCTCTGG | 62°C,60 s |
| Rat-PXR | TCTCAGTCTTCCCAGCCAGT | GGCTTTTCTTCCCTCAGACC | 62℃, 60 s |
| Rat-CAR (-1369bp～-1244bp ) | AACAAACCCTTTCCTCCCCA | CCTCTCTCTGACATGGCCAA | 62℃, 60 s |
| Rat-CAR (-239bp～-90bp ) | GGTGGTGGGCTACAAGAGAT | TCTTAACCGCTGAGCCATCT | 62℃, 60 s |
| Rat-CAR (1117bp～1235bp ) | ACTTCTTGGGCATGCTAGGT | CTCCAGGTAGTCTCAGTGCC | 62℃, 60 s |
| Rat-NFκB | TCTACCTCGGTCCCCTCTAC | CGTTTTCCTACTCGTGCAGG | 62℃, 60 s |
| Rat-AP1 | AGAAAGAAGGGCCCAACTGT | ATAGCCCATGATGTCACCCC | 62℃, 60 s |
| Human-CAR | GATGCCAATGAGCCACTGAG | GACCTCTTTGACCCCTGGAA | 62℃, 60 s |

CYP, cytochrome P450; CAR, constitutive androstane receptor; PXR, pregnane X receptor; NFκB, nuclear factor kappa B 1; AP-1 transcription factor subunit.

**Table S5. Primers for overlap PCR in plasmids construction**

| **Genes** | **Forward** | **Reverse** |
| --- | --- | --- |
| Promoter of CAR | GCCCTTGACTTCATCAACTCACAG | GTTTTATGTGGCCTCCAGTTGCTCTC |
| Promoter of CYP3A4 | TCACTGAGCCTCAGTTTCTACATC | TACTTTCCTTACTTATCTCTCTCCTCTG |
| GR overlap | CTTGGAGGTTCTGGAGCCTGGGCACAATCTAGAatggactccaaagaatcattaactcc | GGTCGACGGTATCGATAAGCTtcacttttgatgaaacagaagttttttg |
| CAR overlap | GGAGGTTCTGGAGCCTGGGCACAATCTAGAatggccagtagggaagatgagc | AATTAATTAAGGTACCGGGCCCCCCCTCGAGccagtgtatccagggtgttc |

CAR, constitutive androstane receptor; CYP, cytochrome P450; GR, glucocorticoid receptor.

**Table S6. The main parameters of nifedipine in plasma after intragastrical administration to male rats of PDE and control group on PW12**

| **Indexes** | **Control** | **PDE** |
| --- | --- | --- |
| ***AUC* 0-24 (mg/L*h)** | 1465 ± 755 | 241 ± 133^*^ |
| ***AUC* 0-∞ (mg/L*h)** | 1629 ± 775 | 438 ± 5^*^ |
| ***AUMC* 0-24** | 13131 ± 7787 | 1624 ± 659^*^ |
| ***AUMC* 0-∞** | 25933 ± 20307 | 1747 ± 584 |
| ***MRT* 0-24 (h)** | 8.8 ± 0.9 | 7.5 ± 2.1 |
| ***MRT* 0-∞ (h)** | 14.5 ± 4.4 | 4.0 ± 1.3^*^ |
| ***VRT* 0-24 (h^2^)** | 46 ± 19 | 27 ± 14 |
| ***VRT* 0-∞ (h^2^)** | 145 ± 32 | 32 ± 4^*^ |
| ***MAT* (h)** | 10 ± 4 | 14 ± 8 |
| ***T_max_* (h)** | 1.58 ± 0.72 | 1.00 ± 0.10 |
| ***C_max_* (mg/L)** | 144 ± 112 | 34 ± 18 |
| ***t_1/2α_* (h)** | 4.5 ± 5.0 | 1.2 ± 1.1 |
| ***t_1/2β_* (h)** | 69.30 ± 0.10 | 24.80 ± 38.50 |
| ***t_1/2ka_*** | 3.20 ± 3.20 | 0.32 ± 0.21 |
| ***K*** | 1678 ± 293 | 102 ± 32 |
| ***k_a_*** | 0.4 ± 0.3 | 20.7 ± 31.7 |

Pharmacokinetic parameters were calculated by DAS 2.0 software. *AUC* 0-24: area under the plasma concentration-time curve from 0 to 24 h, *AUC* 0-∞: the area under the plasma concentration-time curve extrapolated to infinity, *AUMC* 0-24: the area under the moment curve from 0 to 24 h, *AUMC* 0-∞: the area under the moment curve extrapolated to infinity, *MRT* 0-24: mean residence time from 0 to 24 h, *MRT* 0-∞: mean residence time extrapolated to infinity, *VRT* 0-24: variance of residence time from 0 to 24 h, *VRT* 0-∞: variance of residence time extrapolated to infinity, *MAT*: mean absorption time, *T_max_*: time to reach maximum concentration, *C_max_*: maximum concentration, *t_1/2α_*: absorption half-time, *t_1/2β_*: elimination half-time, *K*: elimination rate constant, *K_a_*: absorption rate constant.

**Figure S1. Effects of PDE on CYPs expression of offspring. (A)** The representative concentration-time curves of nifedipine in plasma after intragastrical administration to male rats of PDE and control group on PW12, *n* = 3, DEX: 0.2 mg/(kg∙d) dexamethasone in GD9-20; **(B)** the mRNA expression of CYP3A1 in female offspring on GD20, in PW6 and 12, *n* = 12; the mRNA expression of CYPs in **(C)** male and **(D)** female offspring on GD20, *n* = 12, DEX(L): 0.2 mg/(kg∙d) dexamethasone in GD9-20, DEX(H): 0.8 mg/(kg∙d) dexamethasone in GD9-20, N.D., none detected; the mRNA expression of CYPs in **(E)** male and **(F)** female offspring in PW12, *n* = 12, DEX: 0.2 mg/(kg∙d) dexamethasone in GD9-20; the mRNA expression of CYP3A1 and CYP2B1 **(G)** on GD20 and **(H)** in PW12 of male offspring, *n* = 12, PRL: 0.125 mg/(kg∙d) prednisone in GD0-20, PRM: 0.25 mg/(kg∙d) prednisone in GD0-20, PRH: 0.5 mg/(kg∙d) prednisone in GD0-20. The data are presented as mean ± S.E.M., ^*^*P*<0.05, ^**^*P*<0.01 vs. control. PDE, prenatal dexamethasone exposure; CYPs, cytochrome P450s; PW, postnatal week; DEX, dexamethasone; GD, gestational day; PRL: prednisone-low; PRM: prednisone-medium; PRH: prednisone-high; POR, Cytochrome P450 oxidoreductase.

**Figure S2. Identification results related to nucleas proteion extration, GR-target genes and the construction of heterozygote of liver-specific GR knockout mice (genotype: GR^flox/- Alb-Cre/Alb-Cre^). (A)** Purity validation of extracted cellular fraction by detecting protein levels of ACTB, GAPDH, and Histone 3; **(B)** mRNA expression of FKBP5 on GD20 and PW12; **(C)** Map of the final targeting vector; **(D)** site design of *Nr3c1* semi-knockout; **(E)** the protein expression of wild-type and GR^flox/-^ ^Alb-Cre/Alb-Cre^ mice in PW12 in multiple tissues; **(F)** the mRNA expression of the functional gene in heterozygote of liver-specific GR knockout mice in PW12, *n* = 3. The data are presented as mean ± S.E.M., ^*^*P*<0.05, ^**^*P*<0.01 vs. wild-type. GR, glucocorticoid receptor; GAPDH, glyceraldehyde-phosphate dehydrogenase; ACTB, β-actin; FKBP5, FK506 binding protein 5; GD, gestational day; PW, postnatal week; Tsc22d3, transforming growth factor β-inducible gene 22 domain family protein 3; Sgk1, serum and glucocorticoid inducible kinase 1; Tat, tyrosine aminotransferase; Mt1, melatonin receptor; Alb, albumin; Hnf4α, hepatocyte nuclear factor 4α; Pcna, proliferating cell nuclear antigen; Pepck, phosphoenolpyruvate carboxykinase; G6Pase, glucose-6-phosphatase; Srebp1c, sterol regulatory element binding protein 1c; Fasn, fatty acid synthetase.

**Figure S3. Effects of PDE on nuclear receptors expression of offspring. (A)** GR binding to the promoter of NFκB1 and AP1 in male offspring on GD20 and in PW12 by ChIP-PCR, *n* = 2-6, DEX: 0.2 mg/(kg∙d) dexamethasone in GD9-20; **(B)** GR binding to the promoter of FKBP5 in male offspring on GD20 and in PW12, *n* = 12; **(C)** the mRNA expression of transcriptional factors of male offspring on GD20 and PW12, *n* = 12, DEX(L): 0.2 mg/(kg∙d) dexamethasone in GD9-20, DEX(H): 0.8 mg/(kg∙d) dexamethasone in GD9-20; **(D)** the mRNA expression of nuclear receptors of male offspring on GD20 and in PW12, *n* = 12, PRL: 0.125 mg/(kg∙d) prednisone in GD0-20, PRM: 0.25 mg/(kg∙d) prednisone in GD0-20, PRH: 0.5 mg/(kg∙d) prednisone in GD0-20; **(E)** the correlation of hepatic GR and CAR expression in male offspring on GD20 and in PW12, *n* = 12; **(F)** the mRNA expression of transcriptional factors of female offspring on GD20 and in PW12, *n* = 12; **(G)** plasmids and experiment design; **(H)** fluorescence intensity with dexamethasone or RU486 treatment; **(I)** the mRNA expression of CAR and CYP3A4 in HepG2 cell line upon GR overexpression, *n* = 3; **(J)** dual-luciferase assay of GR mutant (A458T and I628A) binding to CAR promoter region; **(K)** the protein expression of RXR of male offspring on GD20 and in PW6 and PW12, *n* = 4; **(L)** Co-immunoprecipitation of RXR and CAR in PW12; **(M)** RXR binding to the promoter of CYP3A1 and CYP2B1 in male offspring on GD20 and in PW12 by ChIP-PCR, *n* = 6; the mRNA expression of **(N)** G6Pase and **(O)** PEPCK of male offspring on GD20 and in PW6 and PW12, *n* = 12. The data are presented as mean ± S.E.M., ^*^*P*<0.05, ^**^*P*<0.01 vs. control. PDE, prenatal dexamethasone exposure; GR, glucocorticoid receptor; NFκB1, Nuclear factor κB 1; AP1; AP-1 transcription factor subunit; GD, gestational day; PW, postnatal week; ChIP-PCR, chromatin immunoprecipitation assay with qPCR; DEX, dexamethasone; FKBP5, FK506 binding protein 5; PRL: prednisone-low; PRM: prednisone-medium; PRH: prednisone-high; CAR, constitutive androstane receptor; RXR, Retinol X receptor; PXR, pregnane X receptor; CYP3A1, cytochrome P450 3A1; G6Pase, glucose-6-phosphatase; PEPCK, phosphoenolpyruvate carboxykinase; HNF4α, hepatocyte nuclear factor 4 alpha; C/EBPα, CAAT/enhancer binding protein alpha.

**Figure S4. GR induced the regulation of CYP3A expression by glucocorticoids was confirmed using HepG2 and LS174T.** The **(A)** mRNA expression, **(B)** total protein expression of nuclear receptors and CYPs, and **(C)** nuclear protein expression of nuclear receptors in HepG2 after treatments of 500 nM DEX or/and 100 nM GR siRNA, *n* = 3-6; the **(D)** mRNA expression, **(E)** total protein expression of nuclear receptors and CYPs, and **(F)** nuclear protein expression of nuclear receptors in HepG2 after treatments of 500 nM DEX or/and 10 μΜ RU486, *n* = 3-6; the **(G)** mRNA expression, **(H)** total protein expression of nuclear receptors and CYPs, and **(I)** nuclear protein expression of nuclear receptors in LS174T after treatments of 500 nM DEX or/and 100 nM GR siRNA, *n* = 3-6; the **(J)** mRNA expression, **(K)** total protein expression of nuclear receptors and CYPs, and **(L)** nuclear protein expression of nuclear receptors in LS174T after treatments of 500 nM DEX or/and 10 μΜ RU486, *n* = 3-6. The data are presented as mean ± S.E.M., ^*^*P*<0.05, ^**^*P*<0.01 vs. control, ^#^*P*<0.05, ^##^*P*<0.01 vs. negative control. GR, glucocorticoid receptor; CYP3A, cytochrome P450 3A; DEX, dexamethasone; siRNA, small interference RNA; RU486, mifepristone.

**Figure S5. High acetylation levels in multiple regions of the CAR promoter region. (A-B)** Epigenetic modification of histone 3 of **(A)** CYP3A1 and **(B)** CYP2B1 on GD20 and in PW12, *n* = 12, DEX: 0.2 mg/(kg∙d) dexamethasone in GD9-20; H3K9, H3K14, and H3K27 acetylation modification in the region of -2000 bp to 2000 bp enriched around TSS of CAR **(C)** on GD20 and **(D)** in PW12, *n* = 3. The data are presented as mean ± S.E.M., ^*^*P*<0.05, ^**^*P*<0.01 vs. control. CAR, constitutive androstane receptor; CYP3A1, cytochrome P450 3A1; CYP2B1, cytochrome P450 2B1; GD, gestational day; PW, postnatal week; DEX, dexamethasone; TSS, transcription start site.

**Figure S6. P300/CBP is involved in the continuous epigenetic modification of CAR.** Screening for HDAC and HAT expression in male offspring **(A)** on GD20 and **(B)** in PW12, *n* = 10-12, DEX: 0.2 mg/(kg∙d) dexamethasone in GD9-20; **(C)** the expression of Sirt1 on GD20, *n* = 4; **(D)** predicted results of GR interaction with P300/CBP on “STRING: <https://cn.string-db.org>”. The data are presented as mean ± S.E.M., ^*^*P*<0.05, ^**^*P*<0.01 vs. control. CBP, cAMP response element-binding protein binding protein; CAR, constitutive androstane receptor; HDAC, histone deacetylase; HAT, histone acetyltransferase; GD, gestational day; PW, postnatal week; DEX, dexamethasone; Sirt1, sirtuin 1; GR, glucocorticoid receptor.

**Figure S7. Glucocorticoids increase the acetylation level of CAR promoter region via P300/CBP in primary hepatocyte.** The mRNA expression of **(A)** P300 and **(B)** CBP, **(C)** GR, P300/CBP binding to the promoter of CAR, and **(D)** epigenetic modification of CAR after treatments of 0, 20, 100, and 500 nM DEX in primary hepatocyte, *n* = 6; the mRNA expression of **(E)** P300 and **(F)** CBP, **(G)** GR, P300/CBP binding to the promoter of CAR, and **(H)** epigenetic modification of CAR after treatments of 0, 300, 600, and 1200 nM CORT in primary hepatocyte, *n* = 6; **(I)** the protein expression of P300/CBP after treatments of 0, 20, 100, and 500 nM DEX and 0, 300, 600, and 1200 nM CORT in primary hepatocyte, *n* = 3; **(J)** detection of co-immunoprecipitation of GR and P300/CBP after treatments of 500 nM DEX and 1200 nM CORT in primary hepatocyte. The data are presented as mean ± S.E.M., ^*^*P*<0.05, ^**^*P*<0.01 vs. control. CAR, constitutive androstane receptor; CBP, cAMP response element-binding protein binding protein; GR, glucocorticoid receptor; DEX, dexamethasone; CORT, corticosterone.

**Figure S8. GR was found to be critical for P300/CBP-mediated epigenetic modification of the CAR promoter region in HepG2 and LS174T.** The **(A)** mRNA expression of P300/CBP, **(B)** GR binding to the promoter of CAR, **(C)** epigenetic modification of CAR, and **(D)** detection of co-immunoprecipitation of GR and P300 in HepG2 after treatments of 500 nM DEX or/and 100 nM GR siRNA, *n* = 1-3; the **(E)** mRNA expression of P300/CBP, **(F)** GR binding to the promoter of CAR, **(G)** epigenetic modification of CAR, and **(H)** detection of co-immunoprecipitation of GR and P300 in HepG2 cell after treatments of 500 nM DEX or/and 10 μΜ, *n* = 1-3; the **(I)** mRNA expression of P300/CBP, **(J)** GR binding to the promoter of CAR, **(K)** epigenetic modification of CAR, and **(L)** detection of co-immunoprecipitation of GR and P300 in LS174T after treatments of 500 nM DEX or/and 100 nM GR siRNA, *n* = 1-3; the **(M)** mRNA expression of P300/CBP, **(N)** GR binding to the promoter of CAR, **(O)** epigenetic modification of CAR, and **(P)** detection of co-immunoprecipitation of GR and P300 in LS174T cell after treatments of 500 nM DEX or/and 10 μΜ RU486, *n* = 1-3. The data are presented as mean ± S.E.M., ^*^*P*<0.05, ^**^*P*<0.01 vs. control, ^#^*P*<0.05, ^##^*P*<0.01 vs. negative control. GR, glucocorticoid receptor; CBP, cAMP response element-binding protein binding protein; CAR, constitutive androstane receptor; RU486, mifepristone; siRNA, small interference RNA.

**Figure S9. Confirmation of the important role of P300 and CAR for altered glucocorticoid programming CYP3A expression in HepG2.** The **(A)** mRNA expression and **(B)** protein expression of P300, CAR, CYP3A4, and CYP2B6, **(C)** P300 binding to the promoter of CAR, **(D)** epigenetic modification of CAR, and **(E)** detection of co-immunoprecipitation of GR and P300 in HepG2 after treatments of 500 nM DEX or/and 100 nM P300 siRNA, *n* = 3-6; the **(F)** mRNA expression of CAR, CYP3A4, and CYP2B6, **(G)** total and **(H)** nuclear protein expression of CAR, CYP3A4, and CYP2B6 in HepG2 after treatments of 500 nM DEX or/and 10 nM OA, *n* = 3-6. The data are presented as mean ± S.E.M., ^*^*P*<0.05, ^**^*P*<0.01 vs. control, ^#^*P*<0.05, ^##^*P*<0.01 vs. negative control. CAR, constitutive androstane receptor; CYP3A, cytochrome P450 3A; DEX, dexamethasone; siRNA, small interference RNA; OA, okadaic acid.

**Figure S10. Confirmation of the important role of P300 and CAR for altered glucocorticoid programming CYP3A expression in LS174T.** The **(A)** mRNA expression and **(B)** protein expression of P300, CAR, CYP3A4, and CYP2B6, **(C)** P300 binding to the promoter of CAR, **(D)** epigenetic modification of CAR, and **(E)** detection of co-immunoprecipitation of GR and P300 in LS174T after treatments of 500 nM DEX or/and 100 nM P300 siRNA, *n* = 3-6; the **(F)** mRNA expression of CAR, CYP3A4, and CYP2B6, **(G)** total and **(H)** nuclear protein expression of CAR, CYP3A4, and CYP2B6 in LS174T after treatments of 500 nM DEX or/and 10 nM OA, *n* = 3-6. The data are presented as mean ± S.E.M., ^*^*P*<0.05, ^**^*P*<0.01 vs. control, ^#^*P*<0.05, ^##^*P*<0.01 vs. negative control. CAR, constitutive androstane receptor; CYP3A, cytochrome P450 3A; DEX, dexamethasone; siRNA, small interference RNA; OA, okadaic acid.

**Figure S11. Endogenous glucocorticoid-hydrocortisone was confirmed to program expression of CYP3A through GR and CAR in HepG2 and LS174T. (A1-6)** The **(A-1)** mRNA expression of the relative gene after treatments of 0, 300, 600, 1200 nM hydrocortisone in HepG2 cell, *n* = 6; **(A-2)** the total protein expression, **(A-3)** the nuclear protein expression of the relative gene, **(A-4)** GR, P300/CBP binding to the promoter of CAR, **(A-5)** epigenetic modification of CAR after treatments of 1200 nM hydrocortisone in HepG2 cell, *n* = 6, and **(A-6)** detection of co-immunoprecipitation of GR and P300/CBP after treatments of 1200 nM hydrocortisone in HepG2 cell; **(B1-6)** the **(B-1)** mRNA expression of the relative gene after treatments of 0, 300, 600, 1200 nM hydrocortisone in LS174T cell, *n* = 6; **(B-2)** the total protein expression, **(B-3)** the nuclear protein expression of the relative gene, **(B-4)** GR, P300/CBP binding to the promoter of CAR, **(B-5)** epigenetic modification of CAR after treatments of 1200 nM hydrocortisone in LS174T cell, *n* = 6, and **(A-6)** detection of co-immunoprecipitation of GR and P300/CBP after treatments of 1200 nM hydrocortisone in HepG2 cell. The data are presented as mean ± S.E.M., ^*^*P*<0.05, ^**^*P*<0.01 vs. control. CYP3A, cytochrome P450 3A; GR, glucocorticoid receptor; CAR, constitutive androstane receptor; CBP, cAMP response element-binding protein binding protein.

**Figure S12. DEX has an induced memory effect on CYP3A expression in adult offspring exposed to DEX during pregnancy.** The mRNA expression of **(A)** CAR, **(B)** CYP3A1, and **(C)** CYP2B1 after re-administration of DEX in male offspring in PW12, *n* = 6; **(D)** the nuclear protein expression of CAR after re-administration of DEX in male offspring in PW12, *n* = 3; the total protein expression of **(E)** CYP3A1 and **(F)** CYP2B1 after re-administration of DEX in male offspring in PW12, *n* = 3**.** The data are presented as mean ± S.E.M., ^*^*P*<0.05, ^**^*P*<0.01 vs. control, ^#^*P*<0.05, ^##^*P*<0.01 vs. negative control. DEX, dexamethasone; CYP3A, cytochrome P450; CAR, constitutive androstane receptor; PW, postnatal week.

**Appendix material 1.** Vector Report for mNr3c1_Conditional Knockout Project.

*See the* ***PDF*** *file in the zip file* ***Appendix Material 1****.*

**Appendix material 2.** ES Cell Report for Conditional Knockout mNr3c1 Project.

*See the* ***PDF*** *file in the zip file* ***Appendix Material 2****.*

**Appendix material 3.** Methodology validation of HPLC for Detection of enzyme kinetics of liver microsomes.

*See the* ***Word*** *file in the zip file* ***Appendix Material 3****.*

**Western blot original image.**

*See the* ***Word*** *file in the zip file* ***Western blot original image****.*
