# Supplementary material for: The Pivotal Role of GR‐CAR Pathway in Fetal Programming of Hepatic Cytochrome P450 3A Alteration in Adulthood
Source: Adv Sci (Weinh). 2025 Nov 16;13(6):e15583. doi: 10.1002/advs.202515583 (PMC12866823; doi:10.1002/advs.202515583)
Supplement: Supplementary file 2 — Supporting Information [file ADVS-13-e15583-s001.pdf]

Vector Report for

mNr3c1_Conditional Knockout Project

Table of Contents

Summary 1

Overview of the Targeting Strategy 2

The conditional KO allele will be obtained after Flp-mediated recombination; 3

Map of the Final Targeting Vector 4

Sequence of the Final Targeting Vector 5

Vector Construction 13

Methods 21

Summary

Project: mNr3c1_Conditional Knockout Project

Project Type: Conditional Knockout

Description: Targeting Vector Generation

Overview of the Targeting Strategy

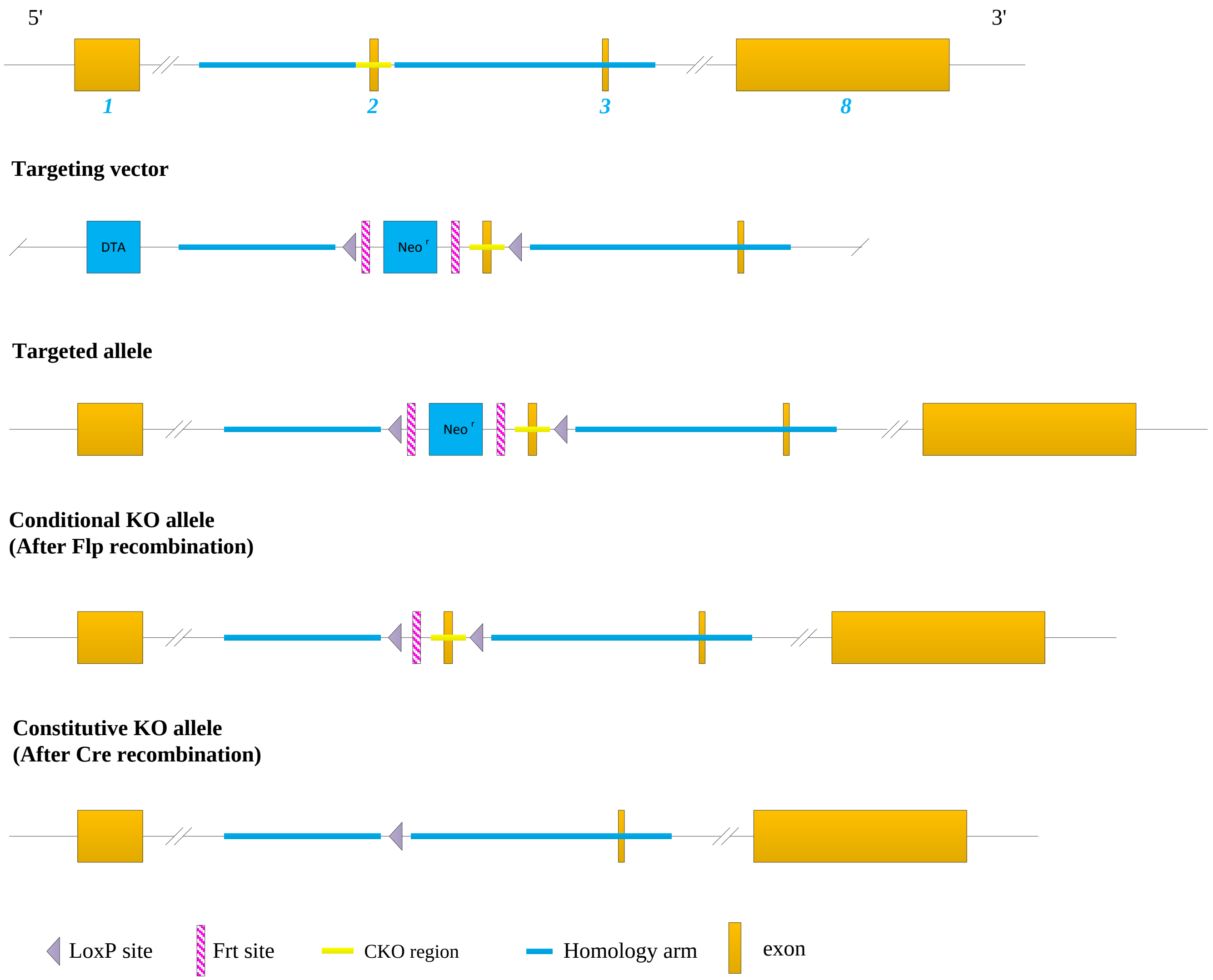

- The mNr3c1 gene (GenBank accession number: NM_008173.3, Ensembl: ENSMUSG00000024431) is located on mouse chromosome 18;
- Eight exons have been identified, with the ATG start codon in exon 1 and TGA stop codon in exon 8;
- Exon 2 will be selected as conditional knockout region. Deletion of exon 2 should result in the loss of function of the mNr3c1 gene;
- To engineer the targeting vector, homology arms and CKO region will be generated by PCR using BAC clone RP23-360K21 or RP23-30O7 from the C57BL/6J library as template;
- In the targeting vector, the Neo cassette will be flanked by Frt sites, and CKO region will be flanked by LoxP sites. DTA will be used for negative selection;
- The conditional KO allele will be obtained after Flp-mediated recombination;
- The constitutive KO allele will be obtained after Cre-mediated recombination;
- C57BL/6 ES cells will be used for gene targeting.

Map of the Final Targeting Vector

Linearization site: **NotI**

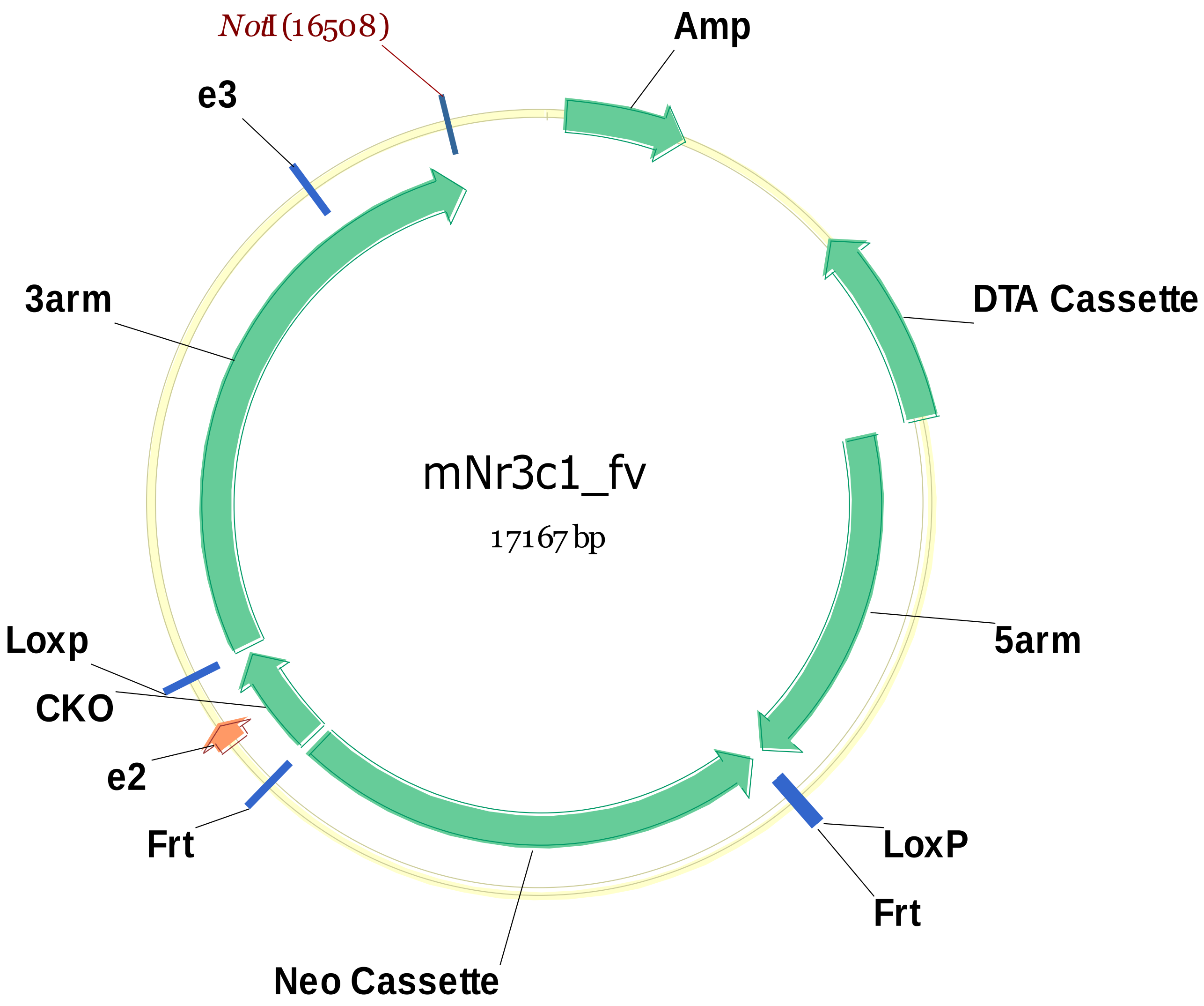

Sequence of the Final Targeting Vector

Homology arms are colored in green, CKO is colored in blue, Frt sites are marked in **bold violet**, LoxP sites are marked in **bold red**. The junctions between 3'arm/5'arm and backbone of mNr3c1 Final Vector which have been sequenced are highlighted in yellow. Exons underlined that have been sequenced are highlighted in yellow too.

```
   1 GTGGCA CTTTTC GGGGAA ATGTGC GCGGAA CCCCTA TTTGTT TATTTT TCTAAA TACATT
  61 CAAATA TGTATC CGCTCA TGAGAC AATAAC CCTGAT AAATGC TTCAAT AATATT GAAAAA
 121 GGAAGA GTATGA GTATTC AACATT TCCGTG TCGCCC TTATTC CCTTTT TTGCGG CATTTT
 181 GCCTTC CTGTTT TTGCTC ACCCAG AAACGC TGGTGA AAGTAA AAGATG CTGAAG ATCAGT
 241 TGGGTG CACGAG TGGGTT ACATCG AACTGG ATCTCA ACAGCG GTAAGA TCCTTG AGAGTT
 301 TTCGCC CCGAAG AACGTT TTCCAA TGATGA GCACTT TTAAAG TTCTGC TATGTG GCGCGG
 361 TATTAT CCCGTA TTGACG CCGGGC AAGAGC AACTCG GTCGCC GCATAC ACTATT CTCAGA
 421 ATGACT TGGTTG AGTACT CACCAG TCACAG AAAAGC ATCTTA CGGATG GCATGA CAGTAA
 481 GAGAAT TATGCA GTGCTG CCATAA CCATGA GTGATA ACACTG CGGCCA ACTTAC TTCTGA
 541 CAACGA TCGGAG GACCGA AGGAGC TAACCG CTTTTT TGCACA ACATGG GGGATC ATGTAA
 601 CTCGCC TTGATC GTTGGG AACCGG AGCTGA ATGAAG CCATAC CAAACG ACGAGC GTGACA
 661 CCACGA TGCCTG TAGCAA TGGCAA CAACGT TGCGCA AACTAT TAACTG GCGAAC TACTTA
 721 CTCTAG CTTCCC GGCAAC AATTAA TAGACT GGATGG AGGCGG ATAAAG TTGCAG GACCAC
 781 TTCTGC GCTCGG CCCTTC CGGCTG GCTGGT TTATTG CTGATA AATCTG GAGCCG GTGAGC
 841 GTGGGT CTCGCG GTATCA TTGCAG CACTGG GGCCAG ATGGTA AGCCCT CCCGTA TCGTAG
 901 TTATCT ACACGA CGGGGA GTCAGG CAACTA TGGATG AACGAA ATAGAC AGATCG CTGAGA
 961 TAGGTG CCTCAC TGATTA AGCATT GGTAAC TGTCAG ACCAAG TTTACT CATATA TACTTT
1021 AGATTG ATTTAA AACTTC ATTTTT AATTTA AAAGGA TCTAGG TGAAGA TCCTTT TTGATA
1081 ATCTCA TGACCA AAATCC CTTAAC GTGAGT TTTCGT TCCACT GAGCGT CAGACC CCGTAG
1141 AAAAGA TCAAAG GATCTT CTTGAG ATCCTT TTTTTC TGCGCG TAATCT GCTGCT TGCAAA
1201 CAAAAA AACCAC CGCTAC CAGCGG TGGTTT GTTTGC CGGATC AAGAGC TACCAA CTCTTT
1261 TTCCGA AGGTAA CTGGCT TCAGCA GAGCGC AGATAC CAAATA CTGTTC TTCTAG TGTAGC
1321 CGTAGT TAGGCC ACCACT TCAAGA ACTCTG TAGCAC CGCCTA CATACC TCGCTC TGCTAA
1381 TCCTGT TACCAG TGGCTG CTGCCA GTGGCG ATAAGT CGTGTC TTACCG GGTTGG ACTCAA
1441 GACGAT AGTTAC CGGATA AGGCGC AGCGGT CGGGCT GAACGG GGGGTT CGTGCA CACAGC
1501 CCAGCT TGGAGC GAACGA CCTACA CCGAAC TGAGAT ACCTAC AGCGTG AGCTAT GAGAAA
1561 GCGCCA CGCTTC CCGAAG GGAGAA AGGCGG ACAGGT ATCCGG TAAGCG GCAGGG TCGGAA
1621 CAGGAG AGCGCA CGAGGG AGCTTC CAGGGG GAAACG CCTGGT ATCTTT ATAGTC CTGTCG
1681 GGTTTC GCCACC TCTGAC TTGAGC GTCGAT TTTTGT GATGCT CGTCAG GGGGGC GGAGCC
1741 TATGGA AAAACG CCAGCA ACGCGG CCTTTT TACGGT TCCTGG CCTTTT GCTGGC CTTTTG
```

1801 CTCACA TGTTCT TTCCTG CGTTAT CCCCTG ATTCTG TGGATA ACCGTA TTACCG CCTTTG
1861 AGTGAG CTGATA CCGCTC GCCGCA GCCGAA CGACCG AGCGCA GCGAGT CAGTGA GCGAGG
1921 AAGCGG AAGAGC GCCCAA TACGCA AACCGC CTCTCC CCGCGC GTTGGC CGATTC ATTAAT
1981 GCAGCT GGCACG ACAGGT TTCCCG ACTGGA AAGCGG GCAGTG AGCGCA ACGCAA TTAATG
2041 TGAGTT AGCTCA CTCATT AGGCAC CCCAGG CTTTAC ACTTTA TGCTTC CGGCTC GTATGT
2101 TGTGTG GAATTG TGAGCG GATAAC AATTTC ACACAG GAAACA GCTATG ACCATG ATTACG
2161 CCAAGC TCGAAA TTAACC CTCACT AAAGGG AACAAA AGCTGG AGCTCC ACCGCC CGGGCT
2221 GGTTCT TTCCGC CTCAGA AGCCAT AGAGCC CACCGC ATCCCC AGCATG CCTGCT ATTGTC
2281 TTCCCA ATCCTC CCCCTT GCTGTC CTGCCC CACCCC ACCCCC CAGAAT AGAATG ACACCT
2341 ACTCAG ACAATG CGATGC AATTTC CTCATT TTATTA GGAAAG GACAGT GGGAGT GGCACC
2401 TTCCAG GGTCAA GGAAGG CACGGG GGAGGG GCAAAC AACAGA TGGCTG GCAACT AGAAGG
2461 CACAGT CGAGGC TGATCA GCGAGC TCTAGG ATCTGC ATTCCA CCACTG CTCCCA TTCATC
2521 AGTTCC ATAGGT TGGAAT CTAAAA TACACA AACAAT TAGAAT CAGTAG TTTAAC ACATTA
2581 TACACT TAAAAA TTTTAT ATTTAC CTTAGA GCTTTA AATCTC TGTAGG TAGTTT GTCCAA
2641 TTATGT CACACC ACAGAA GTAAGG TTCCTT CACAAA GAGATC GCCTGA CACGAT TTCCTG
2701 CACAGG CTTGAG CCATAT ACTCAT ACATCG CATCTT GGCCAC GTTTTC CACGGG TTTCAA
2761 AATTAA TCTCAA GTTCTA CGCTTA ACGCTT TCGCCT GTTCCC AGTTAT TAATAT ATTCAA
2821 CGCTAG AACTCC CCTCAG CGAAGG GAAGGC TGAGCA CTACAC GCGAAG CACCAT CACCGA
2881 ACCTTT TGATAA ACTCTT CCGTTC CGACTT GCTCCA TCAACG GTTCAG TGAGAC TTAAAC
2941 CTAACT CTTTCT TAATAG TTTCGG CATTAT CCACTT TTAGTG CGAGAA CCTTCG TCAGTC
3001 CTGGAT ACGTCA CTTTGA CCACGC CTCCAG CTTTTC CAGAGA GCGGGT TTTCAT TATCTA
3061 CAGAGT ATCCCG CAGCGT CGTATT TATTGT CGGTAC TATAAA ACCCTT TCCAAT CATCGT
3121 CATAAT TTCCTT GTGTAC CAGATT TTGGCT TTTGTA TACCTT TTTGAA TGGAAT CTACAT
3181 AACCAG GTTTAG TCCCGT GGTACG AAGAAA AGTTTT CCATCA CAAAAG ATTTAG AAGAAT
3241 CAACAA CATCAT CAGGAT CCATGG CACGCG CTTCTA CAAGGC GCTGGC CGAAGA GGTGCG
3301 GGAGTT TCACGC CACCAA GATCTG CGGCAC GCTGTT GACGCT GTTAAG CGGGTC GCTGCA
3361 GGGTCG CTCGGT GTTCGA GGCCAC ACGCGT CACCTT AATATG CGAAGT GGACCT GGGACC
3421 GCGCCG CCCCGA CTGCAT CTGCGT GTTCGA ATTCGC CAATGA CAAGAC GCTGGG CGGGGT
3481 TTGCTC GACATT GGGTGG AAACAT TCCAGG CCTGGG TGGAGA GGCTTT TTGCTT CCTCTT
3541 GCAAAA CCACAC TGCTCG ACATTG GGTGGA AACATT CCAGGC CTGGGT GGAGAG GCTTTT
3601 TGCTTC CTCTTG AAAACC ACACTG CTCGAT TTGTTA GCAGCC TCGAAT CAACCC GGGCGA
3661 TCCTAG GCGATG AGATCT AGCTGT CGCGAG GTCAGT ATAGCC CAATCA GTTCCA TTCTGT
3721 GTGACA AAAGCT GGTTTT TCCCAC TGATGT CTATGG CACACA GCCTTC CGTGCA GCCTCC
3781 CACATA CTGAGT TCTTAT CCGCTA CATCTA GATGGC TTCCTG AAGCCT GACTTG GACTCC
3841 AGATTC TTGCCT TTTTTA GTTCTA ACTCTG TTGCCA TCTACA TAGATC ACACCT CAGTGA
3901 CCTGAA CATAAG TTTCCA TATATC CTAATT TCTATG TGATCA TTTGGG GTCTAC TGTCCT
3961 AGATTT AAGACT TAAGAT TTGTTT CCCTTA AGAAAA TAGTGC TGGGCA GTGGTG GCTCAC
4021 ACCTTT AATCCC AGCACT TTGAAG GCAAAG GCAGGC GGATTT CTGAGT TTGAGC CCAGCC
4081 TGGTCT ACAGAG TGAGTC TACAGA GAAGCC CTGTCT TGAAAA AAAAAA AAAGAA AAGAAA
4141 AAAAGA AAGAAA ATAGTG AACTTG TATGTT TCAATT CTTTGC TGCAAG TACTAG ACATCT
4201 AAACAT GTTCAC AGGTGA CACTTC TCAGCT TCCTGA TAAAAA CTAGAG CTCATC AGAGCA
4261 GATGAT TCCCAA AAGCTA TAGGAC TGTCAA GTGGGA CTGCCC AGACCA AAACAA ATCAGG

4321 ATTCTC CCTGGT CAGCCA CGTGCT ACTGTT TACATG ACCTCA ACTAGG GATGGT CGCATC
4381 TTATCA CAAGGT TGTTCA GAGGAT AAATGA GATTCC CTTATG GAAGTG TTCTGA AACCAT
4441 GATGTA CCTAAA TACTGT TTAAAA GTCAAA TATATA CATATA TTTTAT ATATGT GAATAT
4501 TATGTT TAAAAC AGGCAA ACGAAA CAATAT AGAATT GACCTT AAAAAT CTTTAA ACAATG
4561 TGCTTA TTTTCC ATAAAC TAAATA TACAAG CAACTT GCTGAT CCCTAA AAATAG ATGTCA
4621 GATGAG AAGAGA TTGGCT GACCAG GAATCG GCGCGT CCTCAG AACAGA AGCTCT GGAGGC
4681 TTTGCT CCCCTA GAACAT TGCTAC TCTGTG TGCTCA CCCCAG GACCAT CGCACT TCAATG
4741 TGGAAC TCTGCC CCAAAG CAGCAG CTTTGC CTTACA AGCCTG CATTAA CCAGAG GCTGTG
4801 CCCGAC CGAAGC AGTCAT GTCTGG ACAGTC TCTTCC ATTAGA GGTTCA CACTTT GCACTC
4861 AGTCCT CTTGCC CAAACC CAGCAA GGAATC AATACC TTGTGA TATGGA AACAAG CAGACA
4921 GTCTAT TGACAG TGCTCA AAGAAC AATGTG AAACCT ATTCCC AGAACT AGATGG TGGGAG
4981 TGCTGC ACTATT GACTAA GCTACT GGATAA ACAGAC AGTTGG CAGCAT GCAGGA AGATTC
5041 ATTTGA GGATTA AATTTA GGGTGA ACCTTG GGTTTA ATGACT TACTTA ATAGCA TTCTAA
5101 CACATT TTTGTT TGTTTC TGTCTT TCTTGA GACATG GTTTCA CAGGGT TTCCCA GAACTG
5161 GCAGAT CCCAGA CCAGGC TCAATT TTATAG CAATCT TTCTGC TTCAGC CTCCTA AATGTT
5221 AGGATT AGAGGG ATAAGT AACAAC TCCCAG CTACAG TTACTA TTTTTT AATTTT TGTTCC
5281 TAAAGA AGCAAT TGAAAA ACATTG TTATTT CTAACT AGAGAA AGAACA ATTTTT AGTTGT
5341 GAATGT ATTAAT CTTTTT TTTTTT TTTTGC ATTTGA GGCTTA GCGCTT CGCTAG GCTGAA
5401 ATAAAA GCATGC ATAAAG GATGAA GAGACA AGCAAC ATCCAG TCTAGC TTATTC CTAAGT
5461 ACCATC TCTTAG CAAAGT TCATAG AAAGAG AGTGCT GATGTG TGTGAA CTTTCC TATATA
5521 AGTGAT AAACAA CATGAT TTACCA TGAGGA GTTCCA CACCCG ACGAGT TGATAC TATTCA
5581 TAGTTT TTAAAG TCTGTT TGAGAG GCTAGA ACAAGA CGAGGA ATTGGC AATATA ATCAGA
5641 AGCAAA AGGCTA GATTAT TTTTAG CAACAC ATATGC TTTTGT TCCAGA GTCAGC ATGTAT
5701 GGCAAA AGTTCC TTAGGA CAATTC AATATT GAAAAT CATTAT CACTTA ATATAA GATGTA
5761 ATAGCT AATTTA TCAATT TTTCAT AAACCT TCGTTG ATTCAG GTAAAG GATAAA TATCCA
5821 TTGTTC TTCCAA ATTCTC TGAAAA ATTAAA GACTTT GAATAA GAACTT TAAACT AATAAT
5881 ATATCT TGCTTA TTCCCG TGCTGG TAAGAA GTTTGC AGAATG AAAGTG AGCCCT GGTCAG
5941 AGCAGC AGACTC GGTTTT TGTTGT TTTCTC AGGATA TTCTGA TTGAGA GAGAGC AGGACG
6001 GTTGTA CTCAGA ATAGAA AACTAG AGGGTT TCCAAT ATACAT GGTAAT GTTTCT AAACTA
6061 ATCTTT CCCAAG TCACAA GAAACT ATCGTT TCAGCT AGGATC AAACTC TAAGGT GCTATG
6121 TGGTTT TTTTTC CCTATA TTAGAG AGCATG GATAGA ATGATA ATGAGA TTAATG TAAGAA
6181 CTTAAA AGGAAA AGAAGA TTGATG TTTAAG GGTTAG AAGGAA TTTAGG GGTGTG GTAGAG
6241 ATATCA TCCTTT CTTGGT TTTTAT GGTAAT TATGGG TCTAAT CCATTT TTTTAA TCTTGC
6301 CAATCA CATACT TAGAAA ATAATG CATTTT TAAAAA ATAGTA ATTATA TGTTAA ATGAAC
6361 CACAAC ATCATT CTGTTT AGAAGC CAGAGA ACTAAT TGGCTC TTGCAC TGTGTA TTTTAT
6421 TTACTG TTGTGT GTATGT TTCACA TGCATG GGAATA TGCAAA TATGTA GAGACC AGAGCA
6481 GCACAT AGGGCA TCTTCT ATAGCG CTCCGC CTTTCT GCCTTG AGACAG GCATGA ATGGTA
6541 ACCTCA CTGTCT GTTTGT GGTGCA CGGCGC GCCTAG TGGATC CACCTA **ATAACT TCGTAT**
6601 **AGCATA CATTAT ACGAAG TTAT**AT TATGTA CCTGAC TGAT**GA AGTTCC TATACT TTCTAG**
6661 **AGAATA GGAACT TC**GAAG GGTTCC GCAAGC TCTAGT CGAGCC CCAGCT GGTTCT TTCCGC
6721 CTCAGA AGCCAT AGAGCC CACCGC ATCCCC AGCATG CCTGCT ATTGTC TTCCCA ATCCTC
6781 CCCCTT GCTGTC CTGCCC CACCCC ACCCCC CAGAAT AGAATG ACACCT ACTCAG ACAATG

6841 CGATGC AATTTC CTCATT TTATTA GGAAAG GACAGT GGGAGT GGCACC TTCCAG GGTCAA
6901 GGAAGG CACGGG GGAGGG GCAAAC AACAGA TGGCTG GCAACT AGAAGG CACAGT CGAGGC
6961 TGATCA GCGAGC TCTAGA GAATTG ATCCCC TCAGAA GAACTC GTCAAG AAGGCG ATAGAA
7021 GGCGAT GCGCTG CGAATC GGGAGC GGCGAT ACCGTA AAGCAC GAGGAA GCGGTC AGCCCA
7081 TTCGCC GCCAAG CTCTTC AGCAAT ATCACG GGTAGC CAACGC TATGTC CTGATA GCGGTC
7141 CGCCAC ACCCAG CCGGCC ACAGTC GATGAA TCCAGA AAAGCG GCCATT TTCCAC CATGAT
7201 ATTCGG CAAGCA GGCATC GCCATG GGTCAC GACGAG ATCATC GCCGTC GGGCAT GCGCGC
7261 CTTGAG CCTGGC GAACAG TTCGGC TGGCGC GAGCCC CTGATG CTCTTC GTCCAG ATCATC
7321 CTGATC GACAAG ACCGGC TTCCAT CCGAGT ACGTGC TCGCTC GATGCG ATGTTT CGCTTG
7381 GTGGTC GAATGG GCAGGT AGCCGG ATCAAG CGTATG CAGCCG CCGCAT TGCATC AGCCAT
7441 GATGGA TACTTT CTCGGC AGGAGC AAGGTG AGATGA CAGGAG ATCCTG CCCCGG CACTTC
7501 GCCCAA TAGCAG CCAGTC CCTTCC CGCTTC AGTGAC AACGTC GAGCAC AGCTGC GCAAGG
7561 AACGCC CGTCGT GGCCAG CCACGA TAGCCG CGCTGC CTCGTC CTGCAG TTCATT CAGGGC
7621 ACCGGA CAGGTC GGTCTT GACAAA AAGAAC CGGGCG CCCCTG CGCTGA CAGCCG GAACAC
7681 GGCGGC ATCAGA GCAGCC GATTGT CTGTTG TGCCCA GTCATA GCCGAA TAGCCT CTCCAC
7741 CCAAGC GGCCGG AGAACC TGCGTG CAATCC ATCTTG TTCAAT GGCCGA TCCCAT GGTTTA
7801 GTTCCT CACCTT GTCGTA TTATAC TATGCC GATATA CTATGC CGATGA TTAATT GTCAAC
7861 AGGCTG CAGGTC GAAAGG CCCGGA GATGAG GAAGAG GAGAAC AGCGCG GCAGAC GTGCGC
7921 TTTTGA AGCGTG CAGAAT GCCGGG CCTCCG GAGGAC CTTCGG GCGCCC GCCCCG CCCCTG
7981 AGCCCG CCCCTG AGCCCG CCCCCG GACCCA CCCCTT CCCAGC CTCTGA GCCCAG AAAGCG
8041 AAGGAG CAAAGC TGCTAT TGGCCG CTGCCC CAAAGG CCTACC CGCTTC CATTGC TCAGCG
8101 GTGCTG TCCATC TGCACG AGACTA GTGAGA CGTGCT ACTTCC ATTTGT CACGTC CTGCAC
8161 GACGCG AGCTGC GGGGCG GGGGGG AACTTC CTGACT AGGGGA GGAGTA GAAGGT GGCGCG
8221 AAGGGG CCACCA AAGAAC GGAGCC GGTTGG CGCCTA CCGGTG GATGTG GAATGT GTGCGA
8281 GGCCAG AGGCCA CTTGTG TAGCGC CAAGTG CCCAGC GGGGCT GCTAAA GCGCAT GCTCCA
8341 GACTGC CTTGGG AAAAGC GCCTCC CCTACC CGGTAG AATTTC GACGAC CTGCAG CCAATC
8401 CGCCTC AGAAGC CATAGA GCCCAC CGCATC CCCAGC ATGCCT GCTATT GTCTTC CCAATC
8461 CTCCCC CTTGCT GTCCTG CCCCAC CCCACC CCCCAG AATAGA ATGACA CCTACT CAGACA
8521 ATGCGA TGCAAT TTCCTC ATTTTA TTAGGA AAGGAC AGTGGG AGTGGC ACCTTC CAGGGT
8581 CAAGGA AGGCAC GGGGGA GGGGCA AACAAC AGATGG CTGGCA ACTAGA AGGCAC AGTCGA
8641 GGCTGA TCAGCG TCAGAT CCGCCT GTTGAT GTAGCT GCTCAG GTAGTC CAGCAC CTCCTG
8701 GCTGAT GATGCC GTTCCA GGCGGG GTATCT GATGCT GCCCTC GGCGCT GCCCTT CAGCTG
8761 CTCGAT GTGCTG CCACTC CTCGAT GGGGTT GGTCTC GTCCTT CAGGGC GATCAT CTCCTT
8821 GCTGAT GGGGTC GTAGGC GTAGTA CCTGGA CACCAG GGCGAA GTAGTG GTCGGG GATGGC
8881 GGTGAT CTGGTG GGTGTA GGTGGT CCTGGC CACGGC GGAGGC CCTCTT GTCGCT CCAGTT
8941 GCCCAC CACGTT TGTCAG CTCGGT CAGGCC CTTCAT GCTCAG AAAGCT GGTCAT CAGGTG
9001 CCTGCC GATGTG GCTCTT AGGGCC GTTCTT GATAGC GAAGAT GGGGTA GGGGGC GTTCTT
9061 CTTCAG GGCCTT GTTGTA GCTGCG CACCAG GTTGTC CTTCAG CAGCTG GTACTC CTGCTT
9121 GTTGCT GCTGCT GTTGCC GGTCCT GTTCAC TCTCTT CAGCAC GGGCTC GCTGTT CCTCAG
9181 GAACTC GTCCAG GTACAC CAGGGG GTCGAT CCTGCC TCTGGC GCTGAA AAAGTA GATGTG
9241 CCTGGA CACGCT TGTCTT GGTCTC GGTCAC CAGGCA CTGAAT GATCAC GCCCAG GTACTT
9301 GTTCTG CACCAG CTTGAA GCTCTT GGGGTC CACGTT CTTGAT GTCGCT GAACCT GCCGCA

9361 GTTGAT GAATGT GGCCAG GAACAG GAACTG GTACAG GGTCTT GGTCTT GGTGAA CCTGCT
9421 GGTGTA CTCGAA GCTGTT CAGGAT CTTCTC GGTGAT CTCCCA GATGCT CTCGCC CTCGGA
9481 CAGCAG GGCCTT CAGCAT CTTCTT GCTGTG GCTGTT GCCCTT GTCGGC CTCCTC GCTGCT
9541 CTCGAA CTGCAG CTGCAG GCTGGA CACGAT GTCGGT GATGTC GCTCTG GTGCTT CTGGCC
9601 GTTGTA AGGGAT GATGGT GAACTC CCAGGC GGGGAT CAGCTT CTTCAG GCTGGC CTCCAG
9661 GATGGT GGCCTT CTGGGT CTTGTA CTTGAA CTGCAG GCTCTT GTTCAC GATGTC GAAGCT
9721 CAGGCT GTTGCT GATGAT GGTGTT GTAGCT CATGAA GGTGGC CCTCTT GATGGC GGTGCC
9781 GTTGTG GGTGAT CATCCA GCACAG GTAGGT CAGCTC GGCGGC ACAGCT GGCGAT CTTCTC
9841 GCCGCT GGGCCT CTCGAA TCTCTC CACGAA CTGCCG CACCAG CACCTT GGGGGG GGTCTT
9901 GCACAG GATGTC GAACTG GCTCAT CACCTT CCTCTT CTTCTT AGGAGC CATGGC CGCAGG
9961 AAAGCA GAGCCC TGAAGC TCCCAT CACCGG CCAATA AGAGCC AAGCCT GCAGTG TGACCT
10021 CATAGA GCAATG TGCCAG CCAGCC TGACCC CAAGGG CCCTCA GGCTTG GGCACA CTGTCT
10081 CTAGGA CCCTGA GAGAAA GACATA CCCATT TCTGCT TAGGGC CCTGAG GATGAG CCCAGG
10141 GGTGGC TTGGCA CTGAAG CAAAGG ACACTG GGGCTC AGCTGG CAGCAA AGTGAC CAGGAT
10201 GCTGAG GCTTTG ACCCAG AAGCCA GAGGCC AGAGGC CAGGAC TTCTCT TGGTCC CAGTCC
10261 ACCCTC ACTCAG AGCTTT ACCAAT GCCCTC TGGATA GTTGTC GGGTAA CGGTGG ACGCCA
10321 CTGATT CTCTGG CCAGCC TAGGAC TTCGCC ATTCCG CTGATT CTGCTC TTCCAG CCACTG
10381 GCTGAC CGGTTG GAAGTA CTCCAG CAGTGC CTTGGC ATCCAG GGCATC TGAGCC TACCAG
10441 GTCCTT CAGTAC CTCCTG CCAGGG CCTGGA GCAGCC AGCCTG CAACAC CTGCCT GCCAAG
10501 CAGAGT GACCAC TGTGGG CACAGG GGACAC AGGGTG GGGCCC ACAACA GCACCA TTGTCC
10561 ACTTGT CCCTCA CTAGTA AAAGAA CTCTAG GGTTGC GGGGGG TGGGGG AGGTCT CTGTGA
10621 GGCTGG TAAGGG ATATTT GCCTGG CCCATG GAGCTA GCTTGG CTGGAC GTAAAC TCCTCT
10681 TCAGAC CT**GAAG TTCCTA TACTTT CTAGAG AATAGG AACTTC** GGAATT CGATAT CAGCTC
10741 TTCATA TCCACC TGACTC TCTTCC TCAATG CTGGGA TTATAG GCATGC ACAATT ACGGCC
10801 TTCTTT TTAAGA AAGTGG TAAAGA TTTGAA CTTATA TCCTCA TGCCTG CTAGGC AAATGA
10861 TCTTAA CCACTG TACCAT CTTGTC CATTTA ATGCAT TTCATA TTTCAT ACAGAA AAATAG
10921 TATATT CCAATA TATTGA AATCAG GTATTG GTGCTT GTTAGC ACTTAA AAGACA GAATTC
10981 AGTGTG AACATG CTGACA TGGAAT GAGAAG GGGGAA GAAATA TATTCT GACATT CCTTTC
11041 ATAATC ATGCCA AGCATA ACCCTG TTTATG CTTCCT ATAGCC CTGGAA TGAGAC CAGATG
11101 TGAGTT CTCCTC CGTCCA GCTCCT CCACAG CAACGG GACCAC CTCCCA AACTCT GCCTGG
11161 TGTGCT CCGATG AAGCTT CGGGAT GCCATT ATGGGG TGCTGA CGTGTG GAAGCT GTAAAG
11221 TCTTCT TTAAAA GAGCAG TGGAAG GTAGTG TGTGTT TTGAAG AGTTTA ATTTTT GTTCCT
11281 ATTTCT CACAGT AAATGT TTAACT GTTCAT TGTATA CAAAGT CCACTA ATGATA GACATT
11341 AGTTCA GGTGCA AGCATG GTGGCT CTTAAA TCCTCC ATTGGG AAGGGA CAGAAG TAAGGA
11401 GAAGAA CTAGAA TAAGTA ATTAAG TTCAGC CTGAGA AAGAAC TAAAGC AAGCAT TGTAAG
11461 GAACGC TGCTGC ACGGAC TGGCAG TACGCT GAGTCT GGGCAG TCCGAG AATGGT GGCTTT
11521 TCATTC CCTGTA AGCAAG CTGGCT TCAAAG CTACCA GCTATA CCAGAC TGACAA AATCAG
11581 TGACCC TGGGAT CGATGC TCGTAC GCAGTA **ATAACT TCGTAT AGCATA CATTAT ACGAAG**
11641 **TTAT**AC GTGTCG ACGGTA CCGAGG AACAAT AAGGGA GGGACA GCATTC TTTGCT TTACTT
11701 TTTTCC TGTTAA ATAAAT AAAGAG GTCATG TTAAGT ACCACA ATGGCT GAGGCC AAATTA
11761 ACAAAG TACAGA GATTAT CAGATA GGTGAA GTCTAG AAATAT GGTGTT TGAAAG AAGATG
11821 ACTGCA AGGTGA TCATGT GTTGAC GGGTTC ACATTC CAGAGC AGCCGG TTAATA TTAGAG

11881 ATAGTT TATATA CCCTAC CCAAAA GTATCT TAATGG AATTAA ACCAGA AAATAA AGCTAC
11941 ATTGAT CTTTCA ATATAC TACAAT TCACAG CCCATT GTTCTT ATTTAA AATGAA CTCATT
12001 CCCTTA TTTTTA TGTAAA AATAAG TATTAG CTATGC TATTTA TAAAAT GTTTAC CATTTA
12061 GTTATT TCAGCA AAGCGT GGTGGC TAGATT ACATTA GTAAGG AAGCCT TTAGCT GTGTAC
12121 GGTGTT AATGAG TAAGCC TCTTGG CTCAGC TTCCTC CCTAAC TTGAGT TTCAGT GTTCTC
12181 AAAGGC ACTTTC CAGCTC TAACAT TCTGTA AACCCT TGATAC TCTAAT TCAAGG AAAAAA
12241 ATTTAT CTAGAC TTGTAA AATCCA TAACAT AGTTTG TAGCAT TTCATT TTTAAA ATTATT
12301 AAATTT TATTTG ATATTA CATACA TTACAT ATATAA CATAAC ATTTTA ACACAT ACTAGA
12361 AATTAT ACTTAA ACCCTG TATTGG CATTAT AAATAC CCAAAC ATAAAT CAGAGT TAAGTT
12421 TTACAC ATTTGT TATTTA TATAAG ACAAAT CTATTT TTTAAT CCAAGA TCCTAT GAGTTC
12481 TCCATG TCCTTA TGCCCA GCTGGT CCTTTT TACTTG TATTCA TTTTAC CTACAA GTATCT
12541 ATAACT GTATTG GTAGAC CATAAG TGGATA GTTACC AATGAC TCTTCA CTTGCC GTGTGT
12601 GAAGTA CATGCA TCCAAA ATCAAT TAGTGG CCCTGT GAACAC TTAGAT CCTGTT AGGATC
12661 TGTGTC TGCCAG ACTAGC AAACAG GTTCAG AAAGTT GAAGTA GCCAAG CTGGGA TTGCAA
12721 CAAAGT GGTCTC CAAGTA AGGTCT TATTCT CAACAA AGATCC TCAAAG ATGCAT CTGACA
12781 AGCCCT TGATAC CTGCTA GATTAT AATTCA GGTAGA TGCTGA AATAAA AGTAAT CACAAG
12841 AGATTT AACTAT TAGCTA CTATAT ACTGAG TAGTTA TGGTGT GTTGAG CACAGT ATCGTC
12901 TGCATA ATTACT TCCAAG CTTGGC AAATTT TCTAAC AGTTCA TAAGAG AACAAA CTCCAA
12961 AAAGTT AATGTT TCCAAG ATCAGT TTCTTG GCATGC CACTAT ACCACT GCTACT GAGAAC
13021 TGCTAT AGAAGC GTAAGC TGATAG TATTAG TTTATA CTTAAT CTCCAG GTACTC ACAGTA
13081 CAGTCT GTAGCC TGCATC TTTTAC ATGTGT TGTTTC CCATTT CCTATA CCATGT TGTTCT
13141 ATGCAA CATTTT ATTGGT CATAGG ATCCAG CTTTGG TATGAT TCTAGA AAGGAT TACACT
13201 ACACCT TATTAG GAGACA GATATC ACTGGC TATCCT GTAAGC TGGCTG CTACAC CAATCA
13261 TAAATA GCAAGG CACTGT CAAGTG CTAAAA GTAGAT TTAGCC AAAAGC ACCAAC AACCAA
13321 CAGCAT TAAAGA GGCTTT GGATGA GCTGAA GGCTCT TCCCTT GACATT AAGAAG TTAGAA
13381 AGGAAA TTTATC AGGTGG CAGAAA ACAAAG GAATTC CAGACG ACTGTC TTAAAA AGACAT
13441 GTAATT GGTAGT ACATGG CCATTC TCATTC TTTGTG AAATAA TGAACA CTAGCT CAAATC
13501 CCCTGT TTTGTT ACAAGG AATGAA TAGACT AGTGGA TCATTC TATTAT ACTAAC CTGAGC
13561 CCTATA GCTAAG ATTCTC AGTTAT ACTACA CACTGG ATAAAG AAATAA ATATCA GCATAA
13621 TACACA GTCAAT CTTTTA GTTAGA CATAAA TAAACA CTGAAT TTTTTC TACTCT AGAAGT
13681 AATTTT TCCCCA GTTTTT CCAGCA ACACTG ATATAA GGATTC TCTGAA AGACAA TGGATG
13741 TAGGGG TGTTTT TATGGG GTAAAA TTTTAG TGCTTT AACTGT GGATAC AATCTG TTGTTT
13801 CTCCTT TTGCTG CATGAT GGAAGT TTTTAA AAAGAA TTTATT TATCCT TATTTA TATGTA
13861 TAAATG TTTACC TGTATA AACACT GTGTGC CTGGTA CCTGCA GAGCTA AAGGGC ATCTGG
13921 TTCCCT AGAATT GGAGTT ATAGAC AGTTGT GACCTG CTGCCA TGTGGG TGCTAG GAACTG
13981 AACCAA AGTCCT TTGCAA GAGCAG CAAGTG TTCTTA ACTACT GAGCCA TCTCTC CAATCC
14041 CATATA ATAGGA TTTTAT TGAGTT AGTTCT TCTATT TTGTGT TTAAGA AAACAG GAGGCT
14101 TATAAA ATACAG AAAGTA TTTGAA AGAGCT AGCATA GATATT AGGCAT GGGAAA GTAACC
14161 CTGAAA TAAAGC CATTAT ATAACT TAACTA TATAGC TAAGTG TATATA TATATA TATATA
14221 TATATA TATATA TATATA TATATA TATATA TATATA TACACA CACATA CATACA TATGAC
14281 TATTAG TCAACT TCGTAA CTGTAT CTTCAC AAAAGA TTAGAC TAATAC AGTTAA CTATGT
14341 ACTCCA AACTCA TATTTA AACAAC ATCATG ATTTTA CATATC AGGAAA AACTAA ATGTTT

14401 TTTTTT TAATCC AAGTCT TAGATT GCCCTA ATAAAG AAAAAA GTCTCT TCTTAG AAATCA
14461 GGTCTA TATATG TTCCAA CTGGTG AACTGA CTGTTA CAATTC TTTCTC ATTTCT TTGAAT
14521 ATGATT TATTCT AATATA AGTAAC ACAGAT TGTTAA TTACTG GTGTAT CTATAT AGACAG
14581 TTAAAT CTTGTG TAGGAA GCTTCT ATACCC CACCCC AGTCAA ATAGAT CCTTCA GTTGTT
14641 TTCATC AACATT TAATAC TCAGAT TAAAGA AGACAG TGCATG TGGGAA ACAGAA ACACTT
14701 TTAGCC TTTTCC TTTTTA ACAGTC TTAGAA TTTTTT GCCAGG AGAGAA CTGAAG TTCATA
14761 ATATGT TCTTCA GTAGTG GATACC AGAAAA TGGAGC CACTAG CTAGCA TTAAGT CAGTAT
14821 TTGAGG CCAAAG GTGACT CCATGC ATCTTT GTATTC TAAAAG TATATT AAATAA TGATCT
14881 TTGTTA GTTTGT TTAGTT TGCCAC ATTTGT GATTTA CCTAAC CAGTGG CTAGAA GTTGGT
14941 AACATT TTACTT CATTTA AAATTG GGAAGA ACTTCT TTTACC TACACA TGATAA TGTTGA
15001 GTGATG AATTAA AGTCAT CTTTTT CCTTAG GGATTC CAAAAG AAAATT TAAGCC TGCTTC
15061 CTTTTA GTCACT TCAACA ATAGAT CAGAAT TCATAA TAAAAG TAATAA TTGCAA ACCCCA
15121 TTTATA ACTGTT AAATAA CTATAG AAAGCA GGTTTT AAAGTT TGATCT TAGAGT ATTCTT
15181 ATGATT TGAATA CATATA TATATG AAATAC TGTAGT TGGGTT GTTTTC ATTGAT TTTAAA
15241 TTCGTT AAGCAG AACAAG TCCTCT CAACAT TGTAAT TCATGC AGATAA ACAGAC TTAACA
15301 TCCTTA GTCAGT GACTTT GTGTCT TGATGA TAGTCT GCTCAA AACAAA AACAGA AAGGGG
15361 ATAGGC TTTTTC TTTTAA AATATC CTTACA GATCTG TGGAAT TTTAAC AATGCC CTTTTA
15421 ATCTCT TATAGG ACAGCA CAATTA CCTTTG TGCTGG AAGAAA TGATTG CATCAT TGATAA
15481 AATTCG AAGAAA AAACTG TCCAGC ATGCCG CTATCG AAAATG TCTTCA AGCTGG AATGAA
15541 CCTGGA AGGTAA TGGAAG CTTAAG GAACTG TCTGCC TGTCAC ATGAGA ACTGAT CCTTTA
15601 CTTTAT AAAAGC TAGCAA GTGAAC CATGTA ATGAAC TCTATA TATGCT TAATTA GCAAAA
15661 TGTTAT ATTAAA ATGGTT TTTTCC ACGATC ATATCA TTTTGT GTGTTA ATTCAA CTACTT
15721 TTGTTA TATACA TTGAAT TTGGGA CAGCCA TGGTGT ATATTA AGTAAA GAGATT ACATAA
15781 TATTGA AAATTA CATAAC TTGGAG CAATTT TGTTTT CATCCT TCTTAC TCCTTT TTTAAA
15841 ACTCAA AACCAT TTCCTA AGATTG GAAAAG TATTGT ACATGA GATCTA GTTCAT ATTCCT
15901 CTCTTA CTTTGC ACTTCA AAAACC ACCACA CCTTTT ACTTGA AAACAT ATGTTT TATTAC
15961 AACTCT GCCAAC AACATT GGATGG GTTGTT TTATGG TTTAAG TATAAA GGAAAA GTAAAA
16021 ACTACT TGTAAG TTATAA TTTGAT ATTCCT GCTTAT ATAAAA AGTTAT ATAATT AACACT
16081 TATTTA AAAACT TCCCTC TGGAAA CGTCAG GGTTTT GCTGTA GTGGAA ATAGCA AGCTCT
16141 GTGAAA GTAGCA TTCTTC CTAACT CATGTT GTTAGT ATTGAC TGTTAC TGTGGT ACATCA
16201 TAGCAA ACGCTC AGCAAA TATAAA GAGAAT ACAAAT TTAGTA CCTGAG TCTGAG CAGCTT
16261 ACTTTC TGTTTA TGCTAC AGTGTC AATCAC AAAATA GTATGG CACTCT ACAAAG GCAATA
16321 CTATTA CAGGAC CAAGAC GGAAGG CCATCT GAGCCT GTCCTG AAAGTC AGACTC ATCCTG
16381 GAAGAA GTAGCA CTGAGC TGCAAA TACAGA CCTTGA GGAGGA ATTAGA TAGCCA TGACAG
16441 AGACGT GCTCAT AGTTGT ACTTTA TAAAGA GCAGTC TTTGGG ATAGAC AAATGG ATCGGT
16501 GGCAGG CGGCCG CGTACC CAATTC GCCCTA TAGTGA GTCGTA TTACAA TTCACT GGCCGT
16561 CGTTTT ACAACG TCGTGA CTGGGA AAACCC TGGCGT TACCCA ACTTAA TCGCCT TGCAGC
16621 ACATCC CCCTTT CGCCAG CTGGCG TAATAG CGAAGA GGCCCG CACCGA TCGCCC TTCCCA
16681 ACAGTT GCGCAG CCTGAA TGGCGA ATGGGA CGCGCC CTGTAG CGGCGC ATTAAG CGCGGC
16741 GGGTGT GGTGGT TACGCG CAGCGT GACCGC TACACT TGCCAG CGCCCT AGCGCC CGCTCC
16801 TTTCGC TTTCTT CCCTTC CTTTCT CGCCAC GTTCGC CGGCTT TCCCCG TCAAGC TCTAAA
16861 TCGGGG GCTCCC TTTAGG GTTCCG ATTTAG TGCTTT ACGGCA CCTCGA CCCCAA AAAACT

16921 TGATTA GGGTGA TGGTTC ACGTAG TGGGCC ATCGCC CTGATA GACGGT TTTTCG CCCTTT
16981 GACGTT GGAGTC CACGTT CTTTAA TAGTGG ACTCTT GTTCCA AACTGG AACAAC ACTCAA
17041 CCCTAT CTCGGT CTATTC TTTTGA TTTATA AGGGAT TTTGCC GATTTC GGCCTA TTGGTT
17101 AAAAAA TGAGCT GATTTA ACAAAA ATTTAA CGCGAA TTTTAA CAAAAT ATTAAC GCTTAC
17161 AATTTA G

Vector Construction

1. Amplification of Homology Arms and cKO

1 2 3 4 M

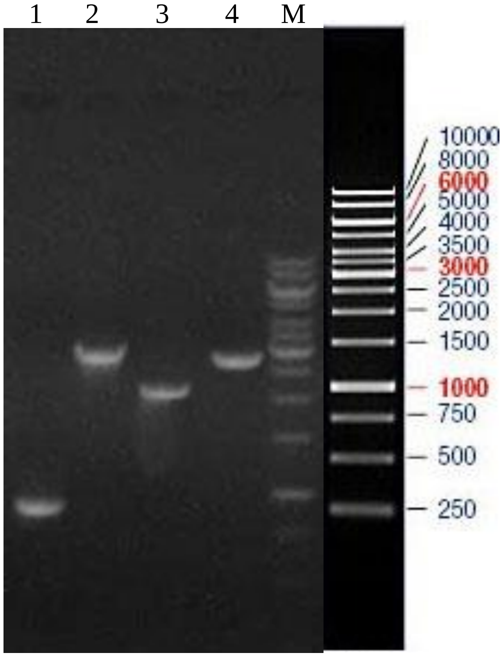

Lane 1: cKO PCR product : 899 bp;

Lane 2: 5’arm PCR product : 2922 bp;

Lane 3: 3’arm-1 PCR product : 2126 bp;

Lane 4: 3’arm-2 PCR product : 2796 Bp.

2. Cloning of cKO

1) Colony PCR

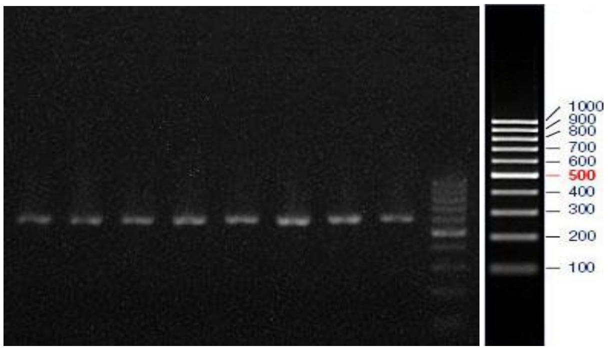

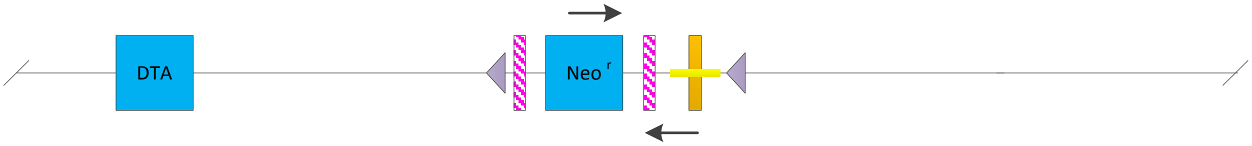

pBasic/cKO

- **PCR Primers:**

Neo-5F: CAGCACCATTGTCCACTTGTCC

CK258-cko-scr-R: AGGCAGAGTTTGGGAGGTGGT

Expected products size: 612 bp

2) Digestion

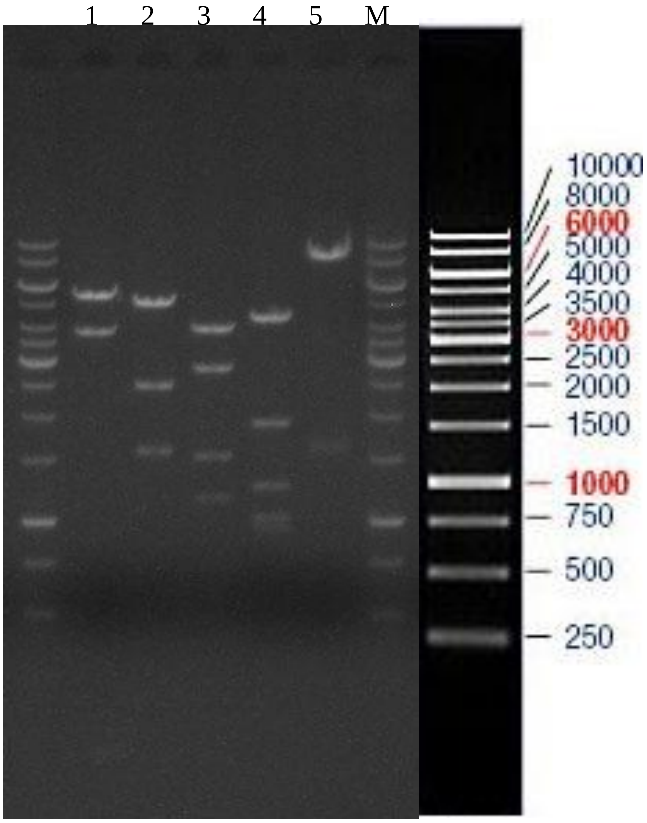

Lane 1: The expected bands with XbaI digestion: 5418 bp + 3731 bp + 317 bp.

Lane 2: The expected bands with AflIII digestion: 4946 bp + 2496 bp + 1581 bp + 443 bp.

Lane 3: The expected bands with FspI digestion: 3990 bp + 2775 bp + 1529 bp + 1172 bp.

Lane 4: The expected bands with BglI digestion: 4404 bp + 1840 bp + 1267 bp + 1018 bp + 937 bp.

Lane 5: The expected bands with DraIII digestion: 7886 bp + 1580 bp.

3. Cloning of 5' Homology Arm

1) Colony PCR

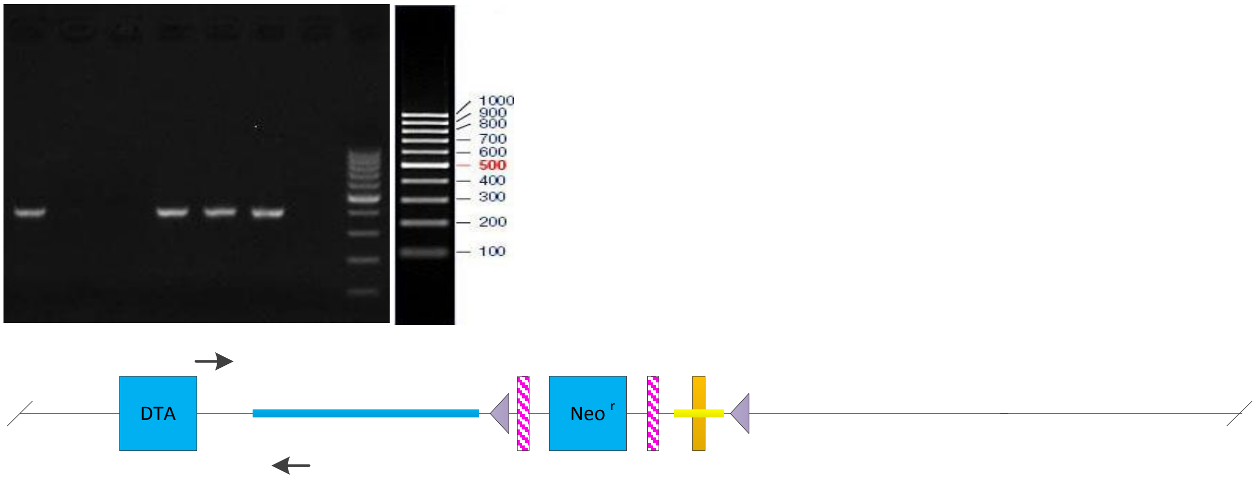

pBasic/cKO/5'HA

- **PCR Primers:**

  LFNeoFL-3r-F: GCATCTGCGTGTTCGAATTCG

  CK258-5arm-scr-R: GGCAAGAATCTGGAGTCCAAGTCA

  Expected product size: 417 bp

2) Digestion

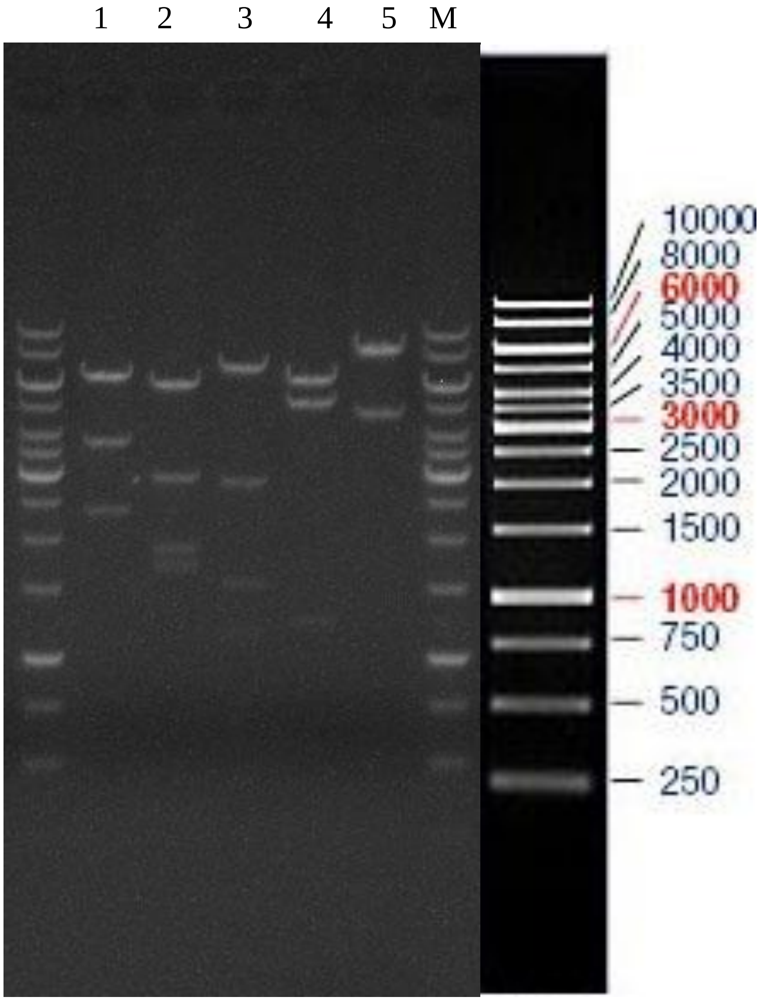

Lane 1: The expected bands with ScaI digestion: 6208 bp + 3754 bp + 2375 bp.

Lane 2: The expected bands with DrdI digestion: 5929 bp + 2906 bp + 1882 bp + 1620 bp.

Lane 3: The expected bands with FspI digestion: 6861 bp + 2775 bp + 1529 bp + 1172 bp.

Lane 4: The expected bands with ApaLI digestion: 6021 bp + 5070 bp + 1246 bp.

Lane 5: The expected bands with EcoRV digestion: 7847 bp + 4490 bp.

4. Cloning of 3’ Homology Arm

1) Colony PCR

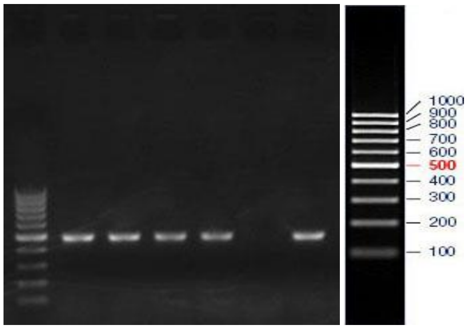

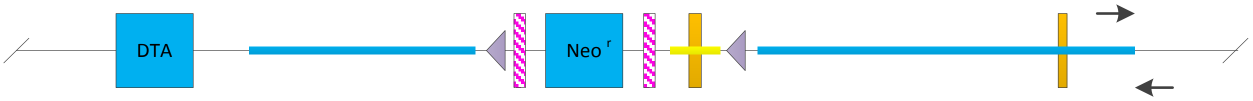

pBasic/CKO/5’HA/3’HA

- **PCR Primers:**

  CK258-3arm-scr-F: GTGGAAATAGCAAGCTCTGTGAAAG

  LFNeoFL_5f-R: GCGATTAAGTTGGGTAACGCC

  Expected product size: 490 bp

2) Digestion

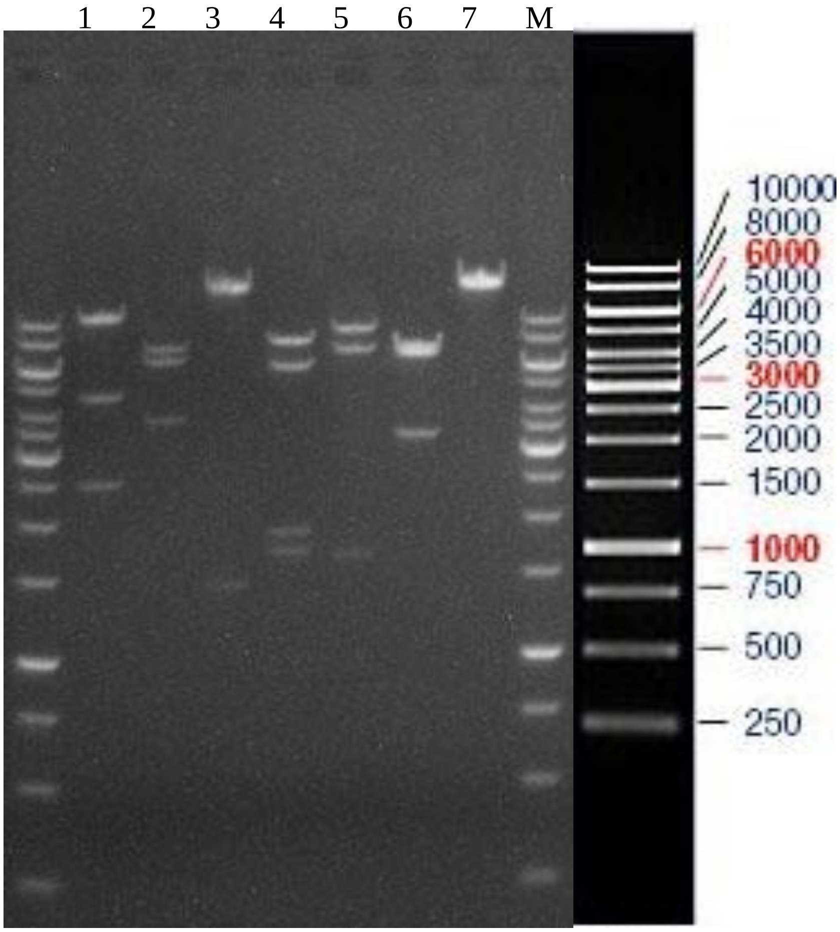

Lane 1: The expected bands with EcoRV digestion: 10188 bp + 4490 bp + 2489 bp.

Lane 2: The expected bands with ScaI digestion: 7205 bp + 6208 bp + 3754 bp.

Lane 3: The expected bands with AvaI digestion: 15728 bp + 1439 bp.

Lane 4: The expected bands with DrdI digestion: 7736 bp + 5929 bp + 1882 bp + 1620 bp.

Lane 5: The expected bands with NdeI digestion: 8603 bp + 6891 bp + 1673 bp.

Lane 6: The expected bands with BamHI digestion: 7259 bp + 6585 bp + 3323 bp.

Lane 7: The expected bands with NotI digestion: 17167 bp.

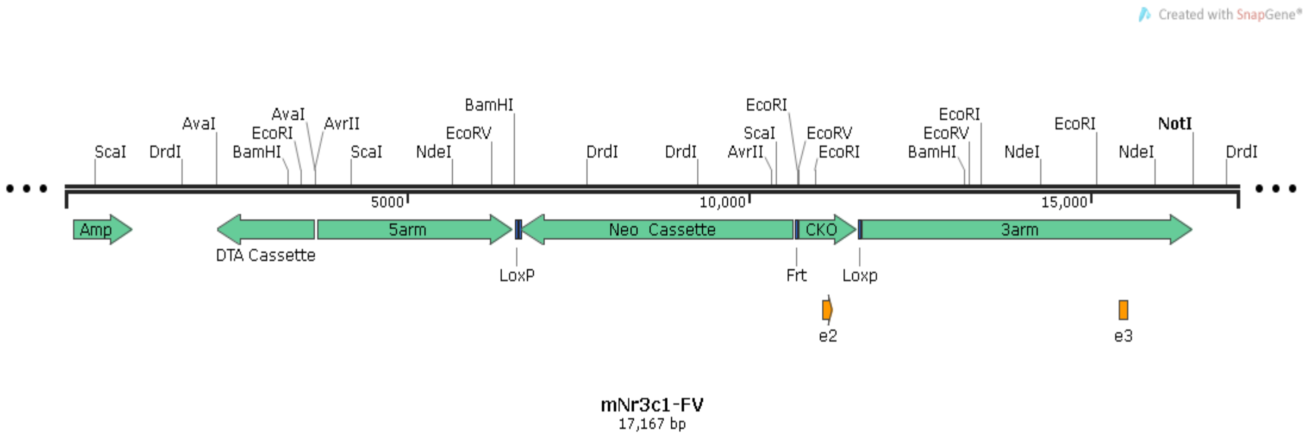

Note：EcoRI/AvrII can be used for southern blot analysis, **NotI** is for vector linearization

Methods

Vector Construction: Mouse genomic fragments were amplified from the BAC clone with high fidelity *Taq* DNA polymerase and were assembled into a targeting vector together with recombination sites and selection markers, as indicated on the vector map. The sequence of the final targeting vector is shown. Further information is available on request.
