# Supplementary material for: The Pivotal Role of GR‐CAR Pathway in Fetal Programming of Hepatic Cytochrome P450 3A Alteration in Adulthood
Source: Adv Sci (Weinh). 2025 Nov 16;13(6):e15583. doi: 10.1002/advs.202515583 (PMC12866823; doi:10.1002/advs.202515583)
Supplement: Supplementary file 3 — Supporting Information [file ADVS-13-e15583-s004.pdf]

ES Cell Report for

Conditional Knockout

mNr3c1 Project

Table of Contents

PCR Screening for mNr3c1 Project 1

PCR Screening Strategy 1

5'Arm PCR Screening 2

LoxP Site PCR Screening 4

Southern Blot Confirmation for mNr3c1 Project 5

Southern Blot Strategy 5

Southern Blot Analysis of the Targeted Clones 6

PCR Screening for mNr3c1 Project

The targeting construct was electroporated into C57BL/6 ES cells. 52 (one 96-well plate) G418-resistant clones were picked. The PCR screening strategy is shown below. The PCR assays were performed with primers: mNr3c1_5'PCR_F / mNr3c1_5'PCR_R and mNr3c1_LoxP_F / mNr3c1_LoxP_R.

PCR Screening Strategy

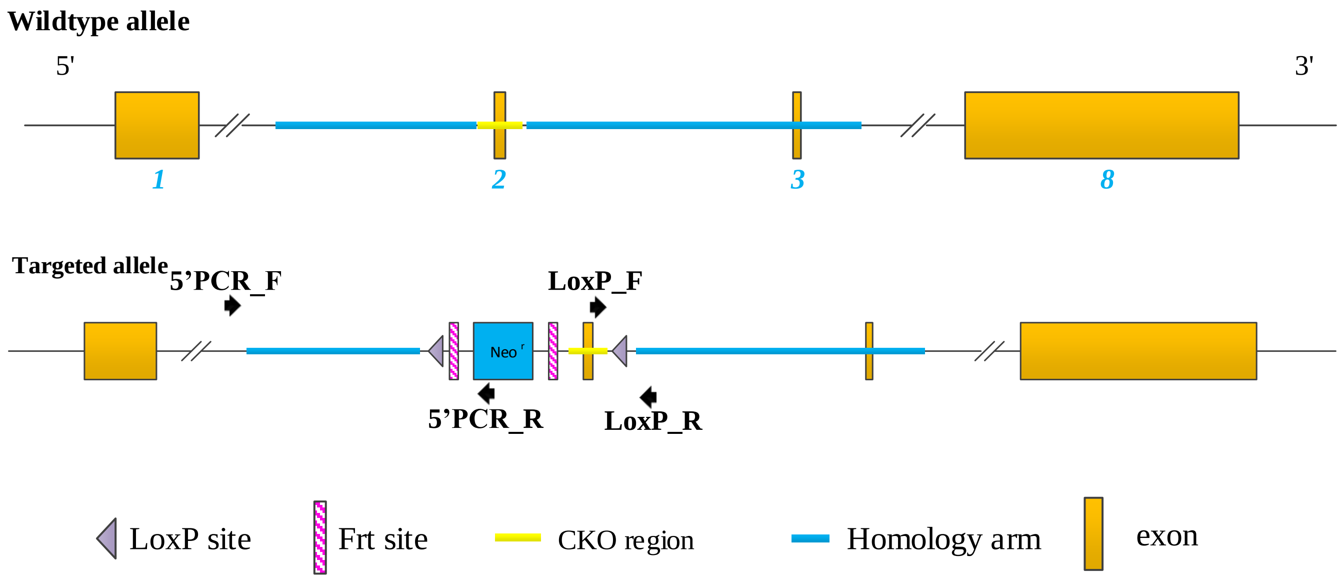

5’Arm PCR Screening

A

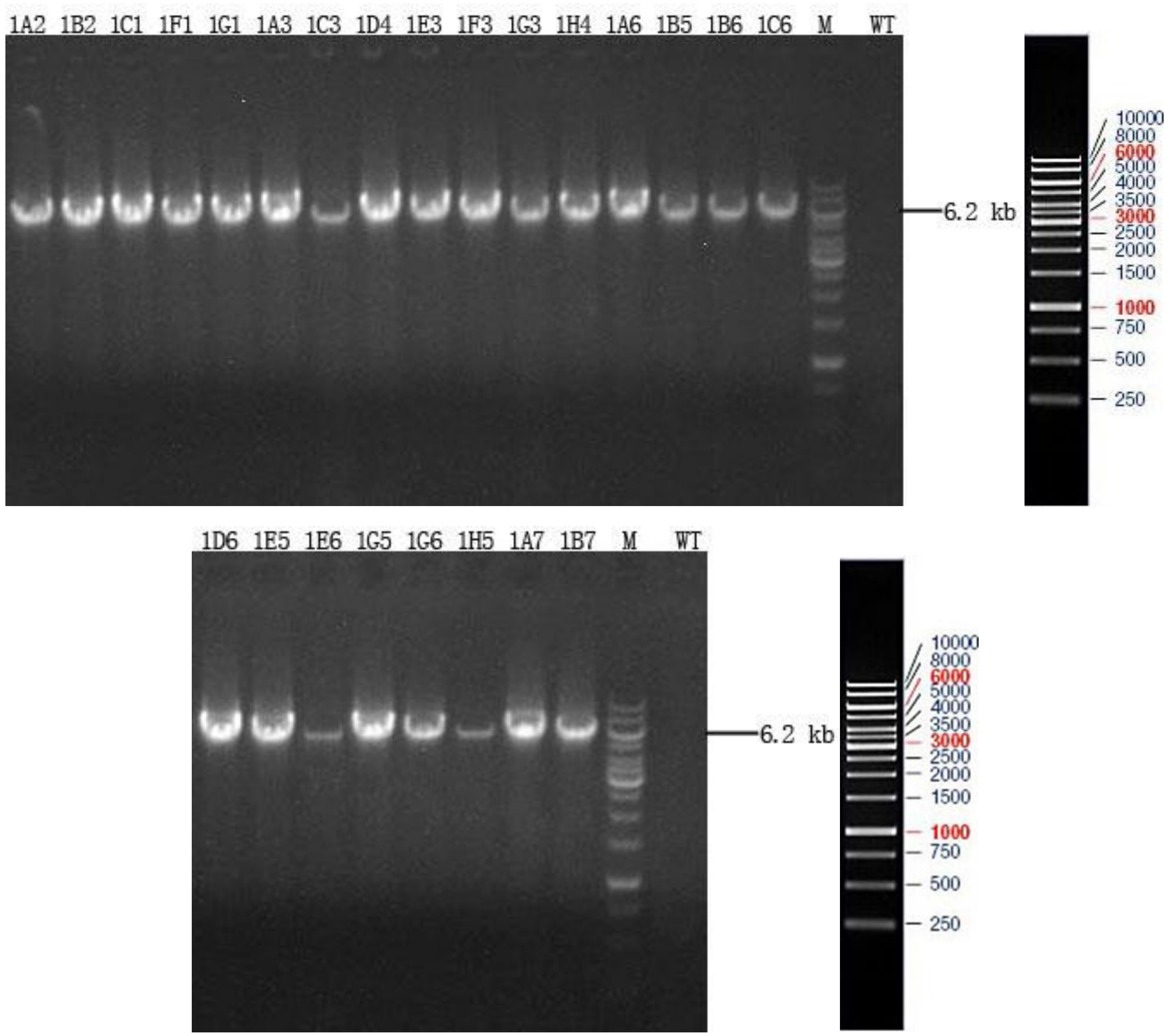

- The expected bands are marked with numbers.
- **A:** 52 clones were screened with mNr3c1_5’PCR_F/ mNr3c1_5’PCR_R primers for a 6.2 kb fragment. There were 24 positive clones: 1A2, 1B2, 1C1, 1F1, 1G1, 1A3, 1C3, 1D4, 1E3, 1F3, 1G3, 1H4, 1A6, 1B5, 1B6, 1C6, 1D6, 1E5, 1E6, 1G5, 1G6, 1H5, 1A7 and 1B7.

- **PCR Screening Primers:**

  mNr3c1_5’PCR_F: CCCATGTAGAAATAGGACAGTAGAGG

  mNr3c1_5’PCR_R: GCTGACCGCTTCCTCGTGCTTTA

LoxP Site PCR Screening

B

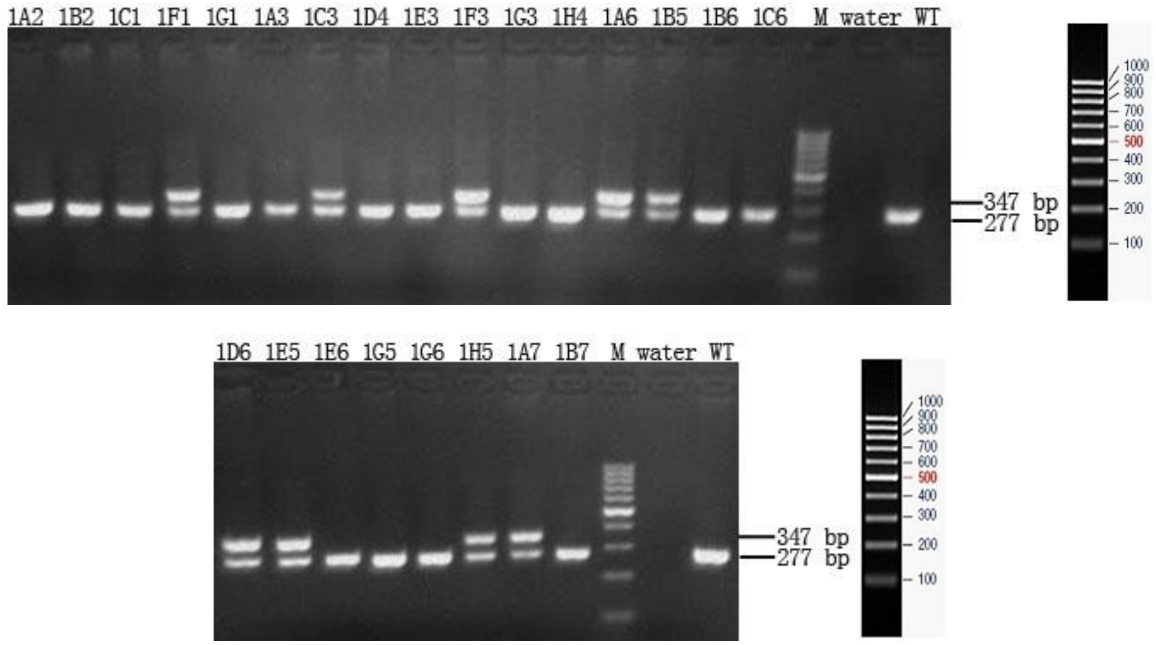

- The top bands represent the positive PCR products of the LoxP site. The bottom bands represent the negative PCR products of the LoxP site.
- **B:** Clones 1A2, 1B2, 1C1, 1F1, 1G1, 1A3, 1C3, 1D4, 1E3, 1F3, 1G3, 1H4, 1A6, 1B5, 1B6, 1C6, 1D6, 1E5, 1E6, 1G5, 1G6, 1H5, 1A7 and 1B7 were screened with mNr3c1_LoxP_F/ mNr3c1_LoxP_R primers for a 277 bp fragment from wildtype allele and a 347 bp fragment from recombinant allele. There were 9 positive clones: 1F1, 1C3, 1F3, 1A6, 1B5, 1D6, 1E5, 1H5 and 1A7.
- **PCR Screening Primers:**

  mNr3c1_LoxP_F: GCAGTCCGAGAATGGTGGCTTT

  mNr3c1_LoxP_R: CGTCAACACATGATCACCTTGCAG

Southern Blot Confirmation for mNr3c1 Project

Six (1A7, 1B5, 1C3, 1D6, 1F1 and 1H5) of nine potential targeted ES clones identified by PCR were expanded for southern blot confirmation. The strategy of southern blot analysis with Neo-probe is shown below. All of the six ES clones were confirmed correct by southern blot analysis.

Southern Blot Strategy

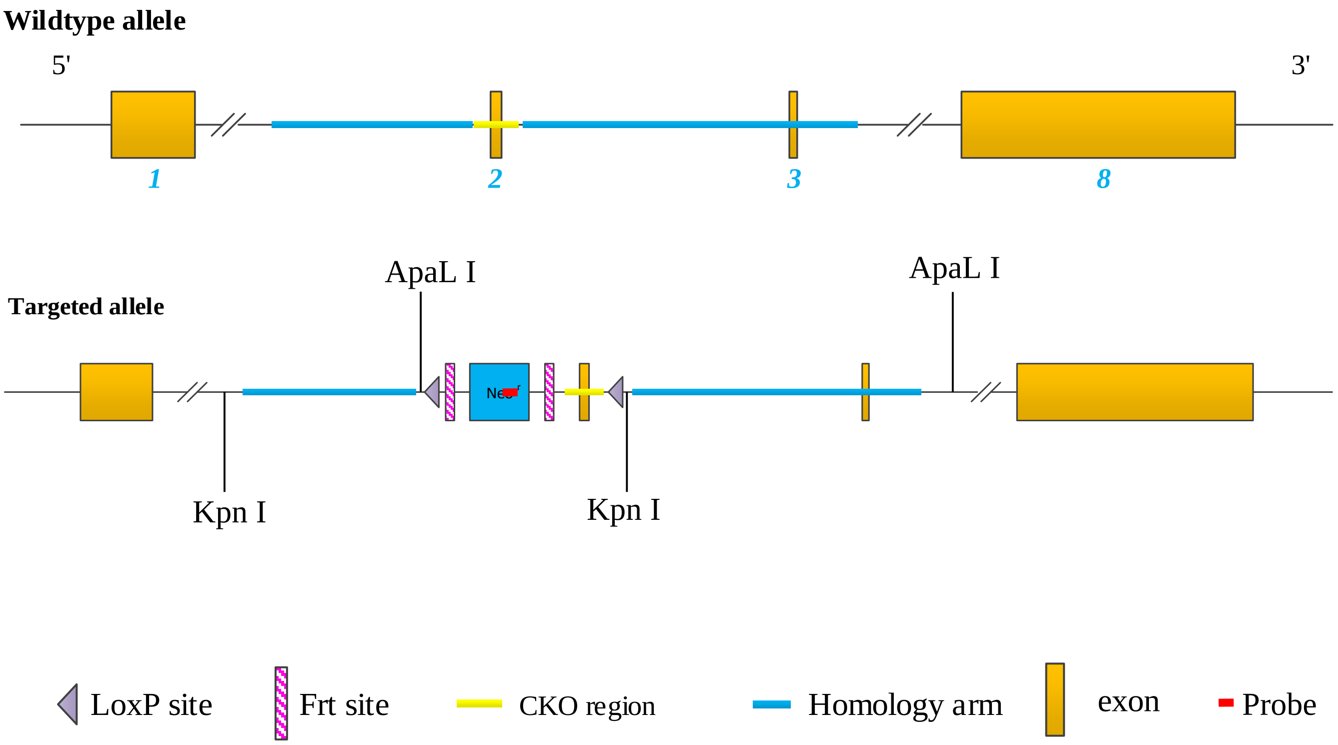

Southern Blot Analysis of the Targeted Clones

C

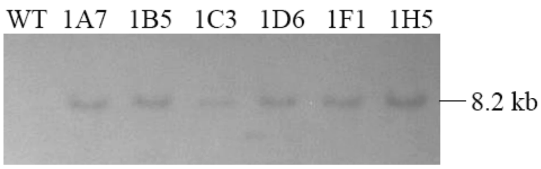

Neo(contain 5'arm): 8.2 kb(MT)

D

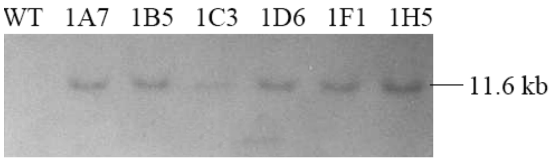

Neo(contain 3'arm): 11.6 kb(MT)

- **C: Neo Probe.** Kpn I: 8.2 kb- recombinant allele.
- **D: Neo Probe.** ApaL I: 11.6 kb- recombinant allele.

  The confirmation analysis showed that all of the six expanded clones (1A7, 1B5, 1C3, 1D6, 1F1 and 1H5) were correctly targeted.

- **Neo Probe Primers:**

  Neo Probe-F: TCATCTCACCTTGCTCCTGC

  Neo Probe-R: AAGGCGATAGAAGGCGATGC
