# Supplementary material for: The Pivotal Role of GR‐CAR Pathway in Fetal Programming of Hepatic Cytochrome P450 3A Alteration in Adulthood
Source: Adv Sci (Weinh). 2025 Nov 16;13(6):e15583. doi: 10.1002/advs.202515583 (PMC12866823; doi:10.1002/advs.202515583)
Supplement: Supplementary file 4 — Supporting Information [file ADVS-13-e15583-s005.docx]

**Appendix material 3: Methodology validation of HPLC for Detection of enzyme kinetics of liver microsomes**

The standard curves were determined using Nifedipine oxide with the concentration ranged from 0.5-60 µmol/L and could cover all sample concentrations. The peak area and concentration of Nifedipine oxide conducted regression analysis. A linear regression analysis was performed on the concentration of oxidized Nifedipine.

**Nifedipine oxide : Y = 48.042X -0.0425，r = 0.992**

Nifedipine oxide solutions of 8, 80, and 160 μM were prepared and then subjected to the experimental method for processing and measurement. Compare the measured concentrations with the prepared concentration to determine the recovery rate.

**Table 1. The recovery rate of oxidized Nifedipine (*n* = 3)**

| concentration | Mean ± SD | Accuracy | Precision |
| --- | --- | --- | --- |
| (µmol/L) | (µmol/L) | (RE, %) | (RSD, %) |
| 8.00 | 8.27±0.28 | 103.36 | 3.36 |
| 80.00 | 71.93±7.97 | 89.91 | 11.08 |
| 160.00 | 168.90±6.23 | 103.69 | 3.69 |

3 replicates for each sample were prepared and the concentration of samples were determined to demonstrate the precision and accuracy. The samples of precision and accuracy were required to be below 20 %.

**Table 2. Intra and inter-day precision and accuracy for oxidized Nifedipine (*n* = 3)**

| concentration | Intra-day variation | | | Inter-day variation | | |
| --- | --- | --- | --- | --- | --- | --- |
|  | Mean±SD | Precision | Accuracy | Mean±SD | Precision | Accuracy |
| (µmol/L) | (µmol/L) | (RSD, %) | (RE, %) | (µmol/L) | (RSD, %) | (RE, %) |
| 8.00 | 7.60±0.61 | 8.03 | 95.00 | 8.27±0.23 | 2.78 | 103.38 |
| 12.00 | 11.46±0.31 | 2.71 | 95.50 | 12.66±1.29 | 10.18 | 105.50 |
| 120.00 | 113.37±1.95 | 1.72 | 94.48 | 119.63±0.56 | 0.47 | 99.69 |

**Details of HPLC-MS method establishment**

The Phenomenex C18 column (4 mm× 2.0 mm, 3 µm) served as a guard column was equipped. A total flow rate was set as 0.3 mL/min with a 10 μL injection volume and column temperature maintained at 40°C.

Mobile phase: 10 mM ammonium formate (pH adjusted to 4.0 with formic acid) and acetonitrile with the following gradient program:

0–0.5 min, 30% acetonitrile;

0.5–3.6 min, linear increase from 30% to 90% acetonitrile;

3.6–4.3 min, maintained at 90% acetonitrile;

4.3–5.0 min, re-equilibrated to 30% acetonitrile.

**HPLC-MS Ion source condition**

An electrospray ionization (ESI) source was set to 5500 V, operating at a temperature of 500°C with positive ion detection. The gas parameters for the ion source were as follows: Ion Source Gas 1 (N2): 30 psi; Ion Source Gas 2 (N2): 30 psi; Curtain Gas (N2): 35 psi; Collision Gas Pressure (CAD Gas, N2): 7 psi. The scanning method employed was Multiple Reaction Monitoring (MRM), which was used for quantitative analysis of the following ionic reactions: Nifedipine (m/z 347.1 → m/z 254.1), Oxidized Nifedipine (m/z 345.2 → m/z 283.9), and Internal Standard Amlodipine (m/z 409.0 → m/z 238.0). The de-clustering potential (DP) was set at 60 V, while the collision energy (CE) was maintained at 25 V. The exit chamber voltage (CXP) was configured to be at +20 V, and the entrance chamber voltage (EP) was established at +10 V.

**Methodology validation of HPLC-MS**

500 μL of blank plasma was taken, and standard solution was accurately added in sequence. Nifedipine was prepared to create plasma samples corresponding to concentrations of 0.5, 1, 2.5, 5, 10, 20, 50, 100, 160, and 200 ng/mL. The samples were processed according to the aforementioned sample pretreatment method and analyzed using LC-MS/MS detection; results were recorded accordingly. A linear regression analysis was performed on the ion intensity ratio (Y) of the standard substance to the internal standard against the concentration of nifedipine (C in ng/mL). The regression equation is as follows: The minimum detectable concentration is established at 0.5 ng/mL

**Nifedipine :Y = 0.0662X + 0.399，r = 0.9967**

Nifedipine was prepared to create plasma samples corresponding to concentrations of 5, 50, and 500 ng/mL and then subjected to the experimental method for processing and measurement. Compare the measured concentrations with the prepared concentration to determine the recovery rate.

**Table 3. The recovery rate of Nifedipine (*n* = 3)**

| concentration | Mean±SD | Accuracy | Precision |
| --- | --- | --- | --- |
| (ng/mL) | (ng/mL) | (RE, %) | (RSD, %) |
| 5.00 | 5.07±0.51 | 101.13 | 3.36 |
| 50.00 | 49.60±2.06 | 99.22 | 1.55 |
| 500.00 | 503.75±17.80 | 100.70 | 1.61 |

Nifedipine was prepared to create plasma samples corresponding to 5.00, 50.00, and 500.00 ng/mL for intra-day test, while 2.50, 50.00, and 250.00 ng/mL for inter-day test. 3 replicates for each sample were prepared and the concentration of samples were determined to demonstrate the precision and accuracy. The samples of precision and accuracy were required to be below 20 %.

**Table 4. Intra-day precision and accuracy for Nifedipine (*n* = 3)**

| concentration  (ng/mL) | Mean±SD  (ng/mL) | Accuracy  (RE, %) | Precision  (RSD, %) |
| --- | --- | --- | --- |
| 5.00 | 5.07±0.51 | 101.40 | 6.36 |
| 50.00 | 49.60±2.06 | 99.23 | 4.55 |
| 500.00 | 503.75±17.80 | 100.75 | 1.61 |

**Table 5. The inter-day precision and accuracy for Nifedipine (*n* = 3)**

| concentration  (ng/mL) | Mean±SD  (ng/mL) | Accuracy  (RE, %) | Precision  (RSD, %) |
| --- | --- | --- | --- |
| 2.50 | 2.47±0.90 | 98.82 | 2.81 |
| 50.00 | 50.17±2.51 | 105.02 | 2.3 |
| 250.00 | 222.67±7.02 | 89.68 | 14.83 |
