# Supplementary material for: The Pivotal Role of GR‐CAR Pathway in Fetal Programming of Hepatic Cytochrome P450 3A Alteration in Adulthood
Source: Adv Sci (Weinh). 2025 Nov 16;13(6):e15583. doi: 10.1002/advs.202515583 (PMC12866823; doi:10.1002/advs.202515583)
Supplement: Supplementary file 5 — Supporting Information [file ADVS-13-e15583-s003.zip › Western blot original image.docx]

**Western blot original image**

**Figure 1B GD20-CYP3A**

**Figure 1B GD20-CYP2B**

**Figure 1B GD20-GAPDH**

**Figure 1B PW6-CYP3A**

**Figure 1B PW6-CYP2B**

**Figure 1B PW6-GAPDH**

**Figure 1B PW12-CYP3A**

**Figure 1B PW12-CYP2B**

**Figure 1C PW12-GAPDH**

**Figure 1H CYP3A**

**Figure 1H CYP2B**

**Figure 1H GAPDH**

**Figure 1I CYP3A**

**Figure 1I CYP2B**

**Figure 1I GAPDH**

**Figure 1J CYP3A**

**Figure 1J CYP2B**

**Figure 1J GAPDH**

**Figure 1K CYP3A**

**Figure 1K CYP2B**

**Figure 1K GAPDH**

**Figure 2B GD20-GR**

**Figure 2B GD20-GAPDH**

**Figure 2B PW6-GR**

**Figure 2B PW6-GAPDH**

**Figure 2B PW12-GR**

**Figure 2B PW12-GAPDH**

**Figure 2C GD20-GR**

**Figure 2C GD20-H3**

**Figure 2C PW6-GR**

**Figure 2C PW6-H3**

**Figure 2C PW12-GR**

**Figure 2C PW12-H3**

**Figure 2E GR**

**Figure 2E CYP3A11**

**Figure 2E CYP2B10**

**Figure 2E GAPDH**

**Figure 2F GR**

**Figure 2F H3**

**Figure 3E GR (GAPDH)**

**Figure 3E GAPDH**

**Figure 3E GR (H3)**

**Figure 3E H3**

**Figure 3F GR (GAPDH)**

**Figure 3F GAPDH**

**Figure 3F GR (H3)**

**Figure 3F H3**

**Figure 3G GR (GAPDH)**

**Figure 3G GAPDH**

**Figure 3G GR (H3)**

**Figure 3G H3**

**Figure 3H GR (GAPDH)**

**Figure 3H GAPDH**

**Figure 3H GR (H3)**

**Figure 3H H3**

**Figure 3J GR**

**Figure 3J CYP3A1**

**Figure 3J CYP2B1**

**Figure 3J GAPDH**

**Figure 3K GR**

**Figure 3K H3**

**Figure 3M GR**

**Figure 3M CYP3A1**

**Figure 3M CYP2B1**

**Figure 3M GAPDH**

**Figure 3N GR (H3)**

**Figure 3N H3**

**Figure 4D GD20-CAR**

**Figure 4D GD20-GAPDH**

**Figure 4D PW6-CAR**

**Figure 4D PW6-GAPDH**

**Figure 4D PW12-CAR**

**Figure 4D PW12-GAPDH**

**Figure 4E GD20-CAR**

**Figure 4E GD20-H3**

**Figure 4E PW6-CAR**

**Figure 4E PW6-H3**

**Figure 4E PW12-CAR**

**Figure 4E PW12-H3**

**Figure 5E CAR (GAPDH)**

**Figure 5E GAPDH**

**Figure 5E CAR (H3)**

**Figure 5E H3**

**Figure 5F CAR (GAPDH)**

**Figure 5F GAPDH**

**Figure 5F CAR (H3)**

**Figure 5F H3**

**Figure 5G CAR (GAPDH)**

**Figure 5G GAPDH**

**Figure 5G CAR (H3)**

**Figure 5G H3**

**Figure 5H CAR (GAPDH)**

**Figure 5H GAPDH**

**Figure 5H CAR (H3)**

**Figure 5H H3**

**Figure 5J CAR (GAPDH)**

**Figure 5J GAPDH**

**Figure 5J CAR (H3)**

**Figure 5J H3**

**Figure 5M CAR (GAPDH)**

**Figure 5M GAPDH**

**Figure 5M CAR (H3)**

**Figure 5M H3**

**Figure 5P CAR (GAPDH)**

**Figure 5P GAPDH**

**Figure 5P CAR (H3)**

**Figure 5P H3**

**Figure 6C GD20-P300**

**Figure 6C GD20-CBP**

**Figure 6C GD20-GAPDH**

**Figure 6C PW12-P300**

**Figure 6C PW12-CBP**

**Figure 6C PW12-GAPDH**

**Figure 6F Fetus Input (P300)**

**Figure 6F Fetus IP GR (IB P300)**

**Figure 6F Fetus GR**

**Figure 6F Fetus GAPDH**

**Figure 6F Fetus Input (CBP)**

**Figure 6F Fetus IP GR (IB CBP)**

**Figure 6F PW12 Input (P300)**

**Figure 6F Fetus IP GR (IB P300)**

**Figure 6F PW12 GR**

**Figure 6F PW12 GAPDH**

**Figure 6F PW12 Input (CBP)**

**Figure 6F PW12 IP GR (IB CBP)**

**Figure 6M (DEX) P300**

**Figure 6M (DEX) CBP**

**Figure 6M (DEX) GAPDH**

**Figure 6M (CORT) P300**

**Figure 6M (CORT) CBP**

**Figure 6M (CORT) GAPDH**

**Figure 6N (DEX) Input (P300)**

**Figure 6N (DEX) IP GR (IB P300)**

**Figure 6N (DEX) GR**

**Figure 6N (DEX) GAPDH**

**Figure 6N (DEX) Input (CBP)**

**Figure 6N (DEX) IP GR (IB CBP)**

**Figure 6N (CORT) Input (P300)**

**Figure 6N (CORT) IP GR (IB P300)**

**Figure 6N (CORT) GR**

**Figure 6N (CORT) GAPDH**

**Figure 6N (CORT) Input (CBP)**

**Figure 6N (CORT) IP GR (IB CBP)**

**Figure 7A-2 P300**

**Figure 7A-2 CBP**

**Figure 7A-2 GAPDH**

**Figure 7A-5 Input (P300)**

**Figure 7A-5 IP GR (IB P300)**

**Figure 7A-5 GR**

**Figure 7A-5 GAPDH**

**Figure 7A-5 Input (CBP)**

**Figure 7A-5 IP GR (IB CBP)**

**Figure 7B-2 P300**

**Figure 7B-2 CBP**

**Figure 7B-2 GAPDH**

**Figure 7B-5 Input (P300)**

**Figure 7B-5 IP GR (IB P300)**

**Figure 7B-5 GR**

**Figure 7B-5 GAPDH**

**Figure 7B-5 Input (CBP)**

**Figure 7B-5 IP GR (IB CBP)**

**Figure 7C-2 P300**

**Figure 7C-2 CBP**

**Figure 7C-2 GAPDH**

**Figure 7C-5 Input (P300)**

**Figure 7C-5 IP GR (IB P300)**

**Figure 7C-5 GR**

**Figure 7C-5 GAPDH**

**Figure 7C-5 Input (CBP)**

**Figure 7C-5 IP GR (IB CBP)**

**Figure 8B P300**

**Figure 8B CBP**

**Figure 8B CAR**

**Figure 8B CYP3A1**

**Figure 8B CYP2B1**

**Figure 8B GAPDH**

**Figure 8C CAR**

**Figure 8C H3**

**Figure 8F Input (P300)**

**Figure 8F IP GR (IB P300)**

**Figure 8F GR**

**Figure 8F GAPDH**

**Figure 8F Input (CBP)**

**Figure 8F IP GR (IB CBP)**

**Figure 8H CAR**

**Figure 8H CYP3A1**

**Figure 8H CYP2B1**

**Figure 8H GAPDH**

**Figure 8I CAR**

**Figure 8I H3**

**Figure 8J CAR**

**Figure 8J CYP3A**

**Figure 8J CYP2B**

**Figure 8J GAPDH**

**Figure 8K CAR**

**Figure 8K H3**

**Figure S2A ACTB**

**Figure S2A GAPDH**

**Figure S2A H3**

**Figure S2E GR**

**Figure S2E GAPDH**

**Figure S3K-GD20-RXR**

**Figure S3K-GD20-GAPDH**

**Figure S3K-PW6-RXR**

**Figure S3K-PW6-GAPDH**

**Figure S3K-PW12-RXR**

**Figure S3K-PW12-GAPDH**

**Figure S3L Input**

**Figure S3L IB**

**Figure S3L RXRa**

**Figure S3L GAPDH**

**Figure S4B-GR**

**Figure S4B-CAR**

**Figure S4B-CYP3A4**

**Figure S4B-CYP2B6**

**Figure S4B-GAPDH**

**Figure S4C-GR**

**Figure S4C-CAR**

**Figure S4C-H3**

**Figure S4E-GR**

**Figure S4E-CAR**

**Figure S4E-CYP3A4**

**Figure S4E-CYP2B6**

**Figure S4E-GAPDH**

**Figure S4F-GR**

**Figure S4F-CAR**

**Figure S4F-H3**

**Figure S4H-GR**

**Figure S4H-CAR**

**Figure S4H-CYP3A4**

**Figure S4H-CYP2B6**

**Figure S4H-GAPDH**

**Figure S4I-GR**

**Figure S4I-CAR**

**Figure S4I-H3**

**Figure S4K-GR**

**Figure S4K-CAR**

**Figure S4K-CYP3A4**

**Figure S4K-CYP2B6**

**Figure S4K-GAPDH**

**Figure S4L-GR**

**Figure S4L-CAR**

**Figure S4L-H3**

**Figure S6C-Sirt**

**Figure S6C-GAPDH**

**Figure S7I-(DEX)-P300**

**Figure S7I-(DEX)-CBP**

**Figure S7I-(DEX)-GAPDH**

**Figure S7I-(CORT)-P300**

**Figure S7I-(CORT)-CBP**

**Figure S7I-(CORT)-GAPDH**

**Figure S7J-(DEX)-Input (P300)**

**Figure S7J-(DEX)-IP GR (IB-P300)**

**Figure S7J-(DEX)-GR**

**Figure S7J-(DEX)-GAPDH**

**Figure S7J-(DEX)-Input (CBP)**

**Figure S7J-(DEX)-IP GR (IB-CBP)**

**Figure S7J-(CORT)-Input (P300)**

**Figure S7J-(CORT)-IP GR (IB-P300)**

**Figure S7J-(CORT)-GR**

**Figure S7J-(CORT)-GAPDH**

**Figure S7J-(CORT)-Input (CBP)**

**Figure S7J-(CORT)-IP GR (IB-CBP)**

**Figure S8D-Input (P300)**

**Figure S8D-IB P300**

**Figure S8D-GR**

**Figure S8D-GAPDH**

**Figure S8H-Input (P300)**

**Figure S8H-IB P300**

**Figure S8H-GR**

**Figure S8H-GAPDH**

**Figure S8L-Input (P300)**

**Figure S8L-IB P300**

**Figure S8L-GR**

**Figure S8L-GAPDH**

**Figure S8P-Input (P300)**

**Figure S8P-IB P300**

**Figure S8P-GR**

**Figure S8P-GAPDH**

**Figure S9B-P300**

**Figure S9B-CAR**

**Figure S9B-CYP3A4**

**Figure S9B-CYP2B6**

**Figure S9B-GAPDH**

**Figure S9E-Input**

**Figure S9E-IB (P300)**

**Figure S9E-GR**

**Figure S9E-GAPDH**

**Figure S9G-CAR**

**Figure S9G-CYP3A4**

**Figure S9G-CYP2B6**

**Figure S9G-GAPDH**

**Figure S9G-CAR (H3)**

**Figure S9G-H3**

**Figure S10B-P300**

**Figure S10B-CAR**

**Figure S10B-CYP3A4**

**Figure S10B-CYP2B6**

**Figure S10B-GAPDH**

**Figure S10E-Input**

**Figure S10E-IB P300**

**Figure S10E-GR**

**Figure S10E-GAPDH**

**Figure S10G-CAR**

**Figure S10G-CYP3A4**

**Figure S10G-CYP2B6**

**Figure S10G-GAPDH**

**Figure S10G-CAR (H3)**

**Figure S10G-H3**

**Figure S11A-2-GR**

**Figure S11A-2-CAR**

**Figure S11A-2-P300**

**Figure S11A-2-CBP**

**Figure S11A-2-CYP3A4**

**Figure S11A-2-CYP2B6**

**Figure S11A-2-GAPDH**

**Figure S11A-3-GR**

**Figure S11A-3-CAR**

**Figure S11A-3-H3**

**Figure S11A-6 Input (P300)**

**Figure S11A-6 IP GR (IB P300)**

**Figure S11A-6 GR**

**Figure S11A-6 GAPDH**

**Figure S11A-6 Input (CBP)**

**Figure S11A-6 IP GR (IB CBP)**

**Figure S11B-2 GR**

**Figure S11B-2 CAR**

**Figure S11B-2 P300**

**Figure S11B-2 CBP**

**Figure S11B-2 CYP3A4**

**Figure S11B-2 CYP2B6**

**Figure S11B-2 GAPDH**

**Figure S11B-3 GR**

**Figure S11B-3 CAR**

**Figure S11B-3 H3**

**Figure S11B-6 Input (P300)**

**Figure S11B-6 IP GR (IB P300)**

**Figure S11B-6 GR**

**Figure S11B-6 GAPDH**

**Figure S11B-6 Input (CBP)**

**Figure S11B-6 IP GR (IB CBP)**

**Figure S12D CAR**

**Figure S12D H3**

**Figure S12E CYP3A1**

**Figure S12E GAPDH**

**Figure S12F CYP2B1**

**Figure S12F GAPDH**
